# Light‐Induced SO Extrusion from Tribenzothiepine *S*‐oxides: A Precursor Approach to the Triphenylene Core

**DOI:** 10.1002/chem.202502655

**Published:** 2025-11-07

**Authors:** Pablo Simón Marqués, Aissam Okba, Nicolas Bréfuel, So Ueno, Nathalie Saffon‐Merceron, Nicolas Ratel‐Ramond, Kyohei Matsuo, Gwénaël Rapenne, Naoki Aratani, Claire Kammerer, Hiroko Yamada

**Affiliations:** ^1^ CEMES, Université de Toulouse, CNRS 29 rue Marvig Toulouse 31055 France; ^2^ Division of Materials Science Nara Institute of Science and Technology, NAIST 8916‐5 Takayama‐cho Ikoma Nara 630‐0192 Japan; ^3^ Institute for Chemical Research Kyoto University Gokasho, Uji Kyoto 611‐0011 Japan; ^4^ Université de Toulouse, Institut de Chimie de Toulouse, ICT UAR 2599 118 route de Narbonne Toulouse 31062 France; ^5^ Université de Toulouse, LPCNO, INSA‐UPS‐CNRS 135 avenue de Rangueil Toulouse 31077 France

**Keywords:** photochemistry, polycyclic aromatic hydrocarbon, precursor approach, sulfoxide, sulfur heterocycles

## Abstract

The precursor approach is a powerful strategy for accessing insoluble or unstable polycyclic aromatic hydrocarbons (PAHs) through the synthesis and solution‐processing of molecular precursors, ultimately converted in situ into functional materials in response to specific stimuli. Developments have mostly focused on retrocycloaddition and decarbonylation as key processes, but the wide structural diversity of PAHs cannot be reached with these sole transformations. It thus appears crucial to develop new reactivity schemes for the precursor approach. Here we investigated the tribenzo[*b*,*d*,*f*]thiepine *S*‐oxide pattern as a photoactivatable precursor for the triphenylene core upon SO extrusion. Synthesis of the tribenzothiepine *S*‐oxide model system was achieved chemo‐ and stereoselectively, exploiting *m*CPBA and a boron‐based Lewis acid to give rise to unprecedented *exo* vs *endo* stable isomers. Spectroscopic analyses complemented by DFT‐based mechanistic studies revealed that these isomers display stereodependent photochemical behavior. Upon UV light irradiation, the *exo* tribenzothiepine *S*‐oxide is converted into triphenylene via a cheletropic photoreaction in yields up to 90% in solution, while the *endo* isomer undergoes first a photoisomerization into the more stable *exo* counterpart. Eventually, light‐induced SO extrusion from *exo*‐tribenzothiepine *S*‐oxide was performed in the solid state, thus highlighting the synthetic potential of cyclic sulfoxides for post‐processing photoconversion as a green route toward triphenylene‐based materials.

## Graphical abstract

Synthesis of tribenzothiepine *S*‐oxide was achieved chemo‐ and stereoselectively to give rise to stable *exo* vs. *endo* sulfoxide isomers displaying stereodependent photochemical behavior. Their light‐induced ring contraction, coupled to a concomitant extrusion of SO, was investigated in solution and in the solid state, en route to post‐processing elaboration of triphenylene‐based materials.

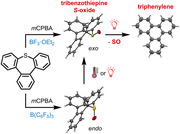

## 1 Introduction

**Scheme 1:**
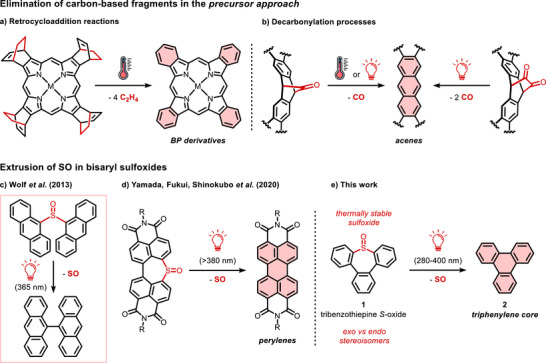
Precursor approach toward insoluble and unstable polycyclic aromatic hydrocarbons, relying on the elimination of carbon‐based fragments (top) and of sulfur monoxide (bottom). a) Synthesis of benzoporphyrins (BPs) by thermally induced retrocycloadditions from soluble tetrabicyclo[2.2.2]octadiene precursors. b) Synthesis of higher acenes by thermally‐ or photoinduced decarbonylation from monoketone or α‐diketone soluble precursors. c) Pioneer work by Wolf et al. on the photoinduced SO extrusion in dianthracenyl sulfoxide, leading to the formation of a new C‐C bond.^[^ ^44^ ^]^ d) Precursor approach strategy for the synthesis of perylene derivatives from soluble dinaphthothiepine *S*‐oxides via SO‐photoextrusion.^[^ ^50^ ^]^ e) Precursor approach strategy for the synthesis of triphenylene pattern via SO‐photoextrusion, exploiting tribenzo[*b*,*d*,*f*]thiepine *S*‐oxide as a soluble precursor.

Over the past decades, the precursor approach has gained significant attention for the preparation of insoluble and unstable polycyclic aromatic hydrocarbons (PAHs). This strategy involves the synthesis of soluble molecular precursors, which can be processed and ultimately transformed into the desired products under specific assemblies, such as thin films.^[^ ^1^ ^]^ Consequently, the precursor approach has been employed in the preparation of organic semiconductors with highly planar structures, which pose challenges for solution processing and are typically restricted to less cost‐efficient vacuum sublimation techniques. A prominent example relates to the benzoporphyrin (BP) derivatives, a family of p‐type semiconductors that have demonstrated excellent performance in organic field‐effect transistors (OFETs),^[^ ^2^ ^]^ organic photovoltaics (OPVs)^[^ ^3^ ^]^ and organic light‐emitting diodes (OLEDs).^[^ ^4^ ^]^ To address the poor solubility of these macrocycles in common processing solvents, their tetrabicyclo[2.2.2]octadiene precursors are synthesized, solution processed, and thereafter quantitatively transformed into the desired BPs by thermal annealing of the thin films at temperatures ranging from 150 to 200 °C (Scheme 1a).^[^ ^5^ ^]^ Beyond BPs, the precursor approach has proven a key tool in the preparation of higher acenes,^[^ ^6^ ^]^ a class of 1D PAHs whose instability is inherent to their structure. Methods involving retrocycloaddition reactions^[^ ^7^, ^8^, ^9^ ^]^ and decarbonylation of monoketone‐^[^ ^10^ ^]^ and α‐diketone‐bridged^[^ ^11^ ^]^ compounds were developed, initially focusing on pentacene (Scheme 1b).^[^ ^12^ ^]^ They were subsequently applied to generate longer acenes in matrices or in bulk phase,^[^ ^13^, ^14^, ^15^, ^16^, ^17^, ^18^, ^19^, ^20^, ^21^ ^]^ as well as further complex structures, including azaacenes, starphenes, and other polyacenes.^[^ ^22^, ^23^, ^24^ ^]^ Lately, the precursor approach has also been extended to metallic surfaces under ultrahigh vacuum conditions,^[^ ^25^, ^26^, ^27^ ^]^ including original synthetic strategies such as the deoxygenation of epoxyacenes^[^ ^28^ ^]^ or the dehydrogenation of hydroacenes.^[^ ^29^, ^30^ ^]^

Strikingly, most of the functional groups involved in the precursor approach are carbon‐based scaffolds that undergo retrocycloaddition, decarbonylation, or oxidation processes, while non‐carbon‐based moieties have been more rarely explored.^[^ ^31^ ^]^ Furthermore, thermally activated strategies have been extensively studied,^[^ ^5^, ^7^, ^8^, ^9^, ^10^, ^15^, ^16^, ^21^, ^32^ ^]^ while precursor conversions triggered by light irradiation remain less exploited.^[^ ^10^, ^11^, ^17^, ^18^, ^19^, ^20^, ^26^ ^]^ In this context, sulfoxides emerge as an interesting functional group with unique photochemical properties that can be tailored by modifying their chemical environment. For instance, asymmetric alkyl‐aryl sulfoxides can undergo photochemical inversion of their configuration, a process involving either the *α*‐cleavage of the sulfinyl moiety or the sp^2^ hybridization of the sulfur atom.^[^ ^33^, ^34^, ^35^, ^36^, ^37^, ^38^ ^]^ This phenomenon has been exploited in various approaches to enrich racemic mixtures with the desired enantiomer.^[^ ^39^, ^40^ ^]^ Additionally, dibenzothiophene *S*‐oxides release atomic oxygen under UV light irradiation, thereby producing the reduced dibenzothiophene counterpart.^[^ ^41^ ^]^ Such photoreactivity has been exploited in solution for intra‐ and intermolecular oxygen transfers,^[^ ^42^ ^]^ and recently transposed onto surfaces.^[^ ^43^ ^]^ Finally, with regard to the precursor approach, bisaryl sulfoxides triggered our attention, owing to their propensity to undergo sulfur monoxide (SO) extrusion with the concomitant creation of a new carbon‐carbon (C‐C) bond, using light as the sole stimulus. Pioneer works on this topic encompass the studies on the oxidation state‐dependent photochemistry of sulfur‐bridged anthracenes by Wolf et al. (Scheme 1c),^[^ ^44^ ^]^ and the use of the photoelimination of SO in the preparation of endohedral fullerene derivatives.^[^ ^45^ ^]^ The late‐stage extrusion of SO has also been exploited for the preparation of organic materials, such as dithienonaphthalene conjugated polymers or *seco*‐hexa‐*peri*‐benzocoronenes.^[^ ^46^, ^47^, ^48^ ^]^ However, in such cases, the elimination of the chalcogen fragment from the heteropine precursors was only promoted by thermal activation or spontaneous decay due to the instability of the seven‐membered ring. Finally, the synthesis of BODIPY dimers^[^ ^49^ ^]^ or perylene diimide (PDI) semiconductors^[^ ^50^, ^51^ ^]^ was successfully achieved with a precursor approach relying on photoinduced SO extrusion. In the latter case, soluble molecular precursors containing a dinaphthothiepine *S*‐oxide pattern underwent ring contraction upon SO elimination to yield the corresponding PDIs in response to several stimuli, including thermal annealing and photoirradiation (Scheme 1d).

Interestingly, the ring contraction occurring upon SO extrusion from cyclic sulfoxides allows to generate structural patterns such as the perylene core, which cannot be obtained by retrocycloaddition or decarbonylation processes. To expand the synthetic utility of this precursor approach beyond the perylene backbone, we devised further PAH scaffolds that could stem from cyclic bisaryl *S*‐oxides upon extrusion of SO. Herein, we report our efforts toward triphenylene‐based materials, focusing on light as an activation method for the post‐processing solid‐state aromatization step, as it represents a greener alternative to thermally induced eliminations. Before tackling more complex structures within this family, such as starphenes,^[^ ^23^, ^52^ ^]^ in this work we investigate bare triphenylene (**2**) as a model system and show that such a scaffold can be selectively obtained from tribenzo[*b,d,f*]thiepine *S*‐oxide (**1**) upon SO photoextrusion (Scheme 1e). We thus developed synthetic routes for the preparation of the sulfoxide soluble precursor and successfully isolated two stable stereoisomers differing in the orientation of the sulfinyl moiety. Alongside their optical characterization, isomerization studies were conducted in order to understand the interconversion pathways between these unique isomers. Eventually, we investigated the cheletropic photoreaction in solution and thin films and performed density functional theory (DFT) calculations to provide insights into the energetics and the mechanistic pathways behind the whole process.

## 2 Results and Discussion

### 2.1 Synthesis of Tribenzothiepine *S*‐oxide (**1**)

**Scheme 2:**
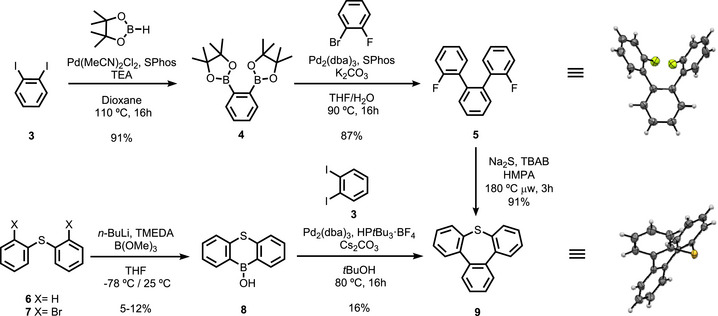
Synthetic routes for the preparation of tribenzothiepine **9** and ORTEP view (50% probability) of the molecular structure of **5** and **9** obtained by single‐crystal X‐ray diffraction.

Our synthetic approach toward tribenzothiepine *S*‐oxide **1** relied on a sulfur‐selective oxidation as the ultimate step, using tribenzothiepine **9** as a key intermediate (Scheme 2). The latter was prepared in three steps from 1,2‐diiodobenzene **3**, starting with a palladium‐catalyzed borylation to afford the corresponding diboronic acid bispinacol ester **4** in excellent yield. The subsequent double Suzuki‐Miyaura coupling with 1‐bromo‐2‐fluorobenzene gave in 87% yield the terphenyl intermediate **5**, incorporating two fluorinated positions prone to undergo nucleophilic aromatic substitution. As reported by Amsharov and coworkers,^[^ ^53^ ^]^ ring‐closure was next performed by means of Na_2_S, using tetrabutylammonium bromide as an additive, in hot HMPA under microwave activation. Tribenzothiepine **9** was obtained in 91% yield, and its structure was unambiguously assigned thanks to single‐crystal X‐ray diffraction analysis, which clearly highlights the boat‐shaped conformation of the 7‐membered thiepine ring.

In this synthetic sequence, the use of the toxic HMPA in the thiepine‐forming step remains a severe limitation, especially regarding upscaling processes. Since substitution of HMPA by other solvents was unsuccessful, an alternative synthetic approach toward tribenzothiepine was explored (Scheme 2, bottom). Following the strategy of Chatani's group for the preparation of oxepines via annulative Suzuki–Miyaura couplings,^[^ ^54^ ^]^ we pursued the synthesis of the diarylborinic acid **8**, which was obtained from diphenylsulfane (**6**) or its dihalogenated analogue **7**.^[^ ^55^, ^56^ ^]^ The twofold cross‐coupling between borinic acid **8** and 1,2‐diiodobenzene **3** indeed furnished the target thiepine **9**, but in poor yield in comparison with the reported oxepine derivatives, presumably due to the deleterious interaction of the sulfide moiety with the catalytic species.

With the tribenzothiepine **9** in hand, first attempts to synthesize the target sulfoxide **1** were carried out following classical conditions for the selective mono‐oxidation of the sulfide moiety. Using potassium peroxymonosulfate (Oxone) as an oxidizing agent, most of the starting material was recovered, and only a poor yield of 27% was obtained after the addition of alumina^[^ ^57^ ^]^ to the reaction mixture (Table 1, Entries 1 and 2). In contrast, by means of *meta*‐chloroperoxybenzoic acid (*m*CPBA), the sulfone **10** was isolated as the major product, and only small amounts of sulfoxide were detected, even at low temperatures (‐80 °C) (Entry 3). To avoid the formation of the sulfone by an overoxidation of the target sulfoxide, we tested the addition of BF_3_·OEt_2_ as previously described for the synthesis of benzothiophene *S*‐oxides upon selective oxidation of the thiophene precursor.^[^ ^58^ ^]^ Tribenzothiepine **9** was thus reacted with *m*CPBA (1.05 equiv.) in the presence of BF_3_·OEt_2_ (8 equiv.) in dichloromethane. Gratifyingly, after 1 h at ‐80 °C, only traces of the sulfone were detected, and the tribenzothiepine *S*‐oxide moiety was formed in 94% yield (Entry 4). Inhibition of overoxidation also took place at ‐10 °C, with a comparable efficiency in sulfoxide formation (Entry 5). In both experiments, purification by column chromatography yielded two distinct fractions corresponding to sulfoxide isomers, as evidenced by mass spectrometry and further spectroscopic analyses. The chemical structures of the latter were finally resolved by single‐crystal X‐ray diffraction (Table 1), and thus confirmed the surprising spatial isomerism of the tribenzothiepine *S*‐oxide moiety, owing to the stereogenic character of the sulfinyl group combined with the locked boat‐shaped conformation of the seven‐membered ring. The first eluted fraction corresponded to the *exo* isomer (*exo*‐**1**), in which the oxygen atom of the pyramidal sulfinyl group is pseudo‐equatorial and points in the same direction as the heterocycle mean plane (Figure S40). In contrast, the second and more polar fraction contained the *endo* isomer *endo*‐**1**, with the sulfinyl moiety standing in a pseudo‐axial position (Figure S42). Both isomers exhibit planar symmetry and are thus achiral. The *exo* isomer was obtained as the major product in the *m*CPBA/BF_3_·OEt_2_ oxidation process, with an *exo*‐**1**/*endo*‐**1**/sulfone **10** ratio of 70:29:1 when the reaction was carried out at ‐10 °C (Entry 5). Density functional theory (DFT) calculations showed that the major *exo* isomer is thermodynamically more stable (ΔG = 6.6 kcal/mol) than its *endo* counterpart (Figure S32), which suffers higher hindrance between the pseudo‐axial oxygen and the lower aromatic ring. Both stereoisomers showed high stability at room temperature, and no interconversion was detected after several weeks, even in solution. However, the derivative *endo*‐**1** was quantitatively converted within a few hours at approx. 110 °C into the more stable *exo*‐**1**, as demonstrated by variable temperature NMR experiments (Figures S22‐S23). Conversely, the same spectrum was recovered after heating a solution of the isomer *exo*‐**1** to temperatures up to 150 °C (Figure S24), thus demonstrating the high thermal stability of the *exo*‐tribenzothiepine *S*‐oxide. Theoretical calculations showed that the less energetic isomerization path from the *endo*‐ to the *exo*‐isomer (ΔG^‡^ = 22.8 kcal/mol) occurs via simultaneous rotation of the two biaryl single bonds incorporated in the tribenzothiepine scaffold (C6‐C7 and C12‐C13, see Table S3 page S29), while the direct epimerization of the sulfinyl group through a change of hybridization at the sulfur atom is highly unfavorable (ΔG^‡^ = 40.7 kcal/mol) (Figure S32). Diastereoisomerism between *meso*‐compounds *exo*‐**1** and *endo*‐**1** is thus promoted by three stereogenic elements, with the two atropoisomeric mirror image biaryl axes endowing the sulfoxide with a stereogenic character.

**Table 1:** Optimization of the tribenzothiepine oxidation conditions to give sulfoxides *exo*‐**1** and *endo*‐**1** and ORTEP view (50% probability) of the molecular structure of *exo*‐**1**, *endo*‐**1** and **10** obtained by single‐crystal X‐ray diffraction.

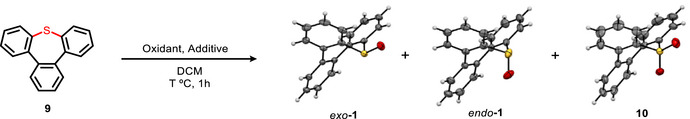					
Entry	Oxidant [equiv.]	Additive [equiv.]	T [°C]	yield [%]^[^ ^a^ ^]^	ratio [%] ***exo***‐1:*endo‐*1:10
1	Oxone® (20)	/	0	0	0:0:0
2	Oxone® (2)	Al_2_O_3_ (10)	25	27	99:0:1
3	*m*CPBA (1.05)	/	−80	52	14:0:86
4	*m*CPBA (1.05)	BF_3_·OEt_2_ (8)	−80	94	66:33:1
5	*m*CPBA (1.05)	BF_3_·OEt_2_ (8)	−10	96	70:29:1
6	*m*CPBA (1.05)	AgTFA (8)	−10	64	56:35:9
7	*m*CPBA (1.05)	HFIP^[^ ^b^ ^]^ (8)	−10	98	43:48:9
8	*m*CPBA (1.05)	BCF^[^ ^c^ ^]^ (8)	−10	95	0:100:0

^[a]^ Combined yield of *exo*‐**1**, *endo*‐**1** and **10**.

^[b]^ HFIP = 1,1,1,3,3,3‐hexafluoroisopropanol.

^[c]^ BCF = tris(2,3,4,5,6‐pentafluorophenyl)borane

From a mechanistic point of view, the selective mono‐oxidation of the tribenzothiepine **9** promoted by the *m*CPBA/BF_3_·OEt_2_ system involves an initial coordination of the sulfur atom to the Lewis acid, present in large excess. As a result, the sulfide is partially shielded, and oxidation by *m*CPBA takes place on the remaining pseudo‐equatorial or pseudo‐axial free position to yield the *exo*‐ and *endo*‐isomers, respectively. We thus reasoned that the nature of the additive should impact the *exo*/*endo* ratio. Indeed, when BF_3_ was substituted with AgTFA as a Lewis acid (Entry 6) or with hexafluoroisopropanol (HFIP) as a hydrogen bond donor (Entry 7), a modification of the proportion of *exo*‐ and *endo*‐sulfoxides was observed. In both cases, it resulted in a lower stereocontrol, with an *exo*/*endo* ratio close to 1:1 in the case of HFIP, presumably due to the higher degree of freedom in the thiepine‐additive adduct in comparison with boron‐based Lewis acids. With the aim to promote a strong sulfur‐to‐boron coordination while controlling the geometry of the resulting adduct, the bulky tris(pentafluorophenyl)borane (BCF) was eventually employed (Entry 8). Oxidation proceeded with complete chemo‐ and stereoselectivity, giving exclusively the *endo*‐tribenzothiepine *S*‐oxide *endo‐* **1** in 95% isolated yield. With this bulky Lewis acid, coordination of sulfur is presumably unfavorable in the pseudo‐axial position due to steric hindrance with the lower aromatic ring in the thiepine. The resulting pseudo‐equatorial location of BCF thus shields the approach of *m*CPBA from this side of the heterocycle, and sulfur oxidation selectively releases the *endo*‐isomer.

The combination of *m*CPBA as an oxidant and a boron‐based Lewis acid as an adjustable additive thus allows the chemoselective oxidation of tribenzothiepine to the corresponding sulfoxide, with a selective access to either diastereoisomer. Using the bulky BCF, the *endo*‐tribenzothiepine *S*‐oxide *endo*‐**1** is directly prepared from the sulfide **9** in 95% yield, while isolation of the *exo*‐counterpart *exo*‐**1** requires a final quantitative thermal isomerization step. The target tribenzothiepine *S*‐oxides were therefore prepared in 4 (*endo*‐**1**) or 5 (*exo*‐**1**) steps from 1,2‐diiodobenzene with an overall yield of 68%.

### 2.2 Structural Comparison of Tribenzothiepine Derivatives **9**, *exo*‐**1**, *endo*‐**1** and **10**

Having in hand four distinct tribenzo[*b*,*d*,*f*]thiepine derivatives along with their single‐crystal X‐ray diffraction analysis data, a systematic comparison of the structural properties of compounds **9**, *exo*‐**1**, *endo* **‐1**, and **10** was undertaken (see Supporting Information, Section 8). Independent of the oxidation state at sulfur, the seven‐membered cycle of all the molecules adopts a boat conformation with higher flagpole strain than those observed in dibenzo[*b*,*f*]‐ and bare thiepine derivatives.^[^ ^59^, ^60^ ^]^ Indeed, the angles between the plane comprised by the S and the two vicinal carbon atoms and the plane comprising the phenyl ring facing the sulfoxide moiety are all in the 83–88° range (Figure S50), while compounds lacking the fused benzene ring opposite to the sulfur atom display values between 95° and 100°. In addition, the angle between the two planes comprising the phenyl rings adjacent to the sulfur atom decreases from dibenzo‐annulated species (ca. 118°) to the synthesized tribenzo[*b,d,f*]thiepines (103–110°), presumably due to the geometric constraints of both C6‐C7 and C12‐C13 biaryl axes (Figure S49a‐d). This hypothesis can be corroborated by the distortion of the dihedral angles within these biphenyl moieties, displaying values in the 45–49° range (Figure S49e‐h).

The bonds’ length within the thiepine rings does not seem to be strongly affected by the functionalization of the sulfur atom, regardless of the disposition or number of oxygens (Table S3). Nonetheless, the *exo*‐stereoisomer of **1** shows an intramolecular short contact between the pseudo‐equatorial oxygen and both hydrogens located in *ortho*‐positions, with distances of approx. 2.50 Å that could be considered as hydrogen bonds (Figure S40c),^[^ ^61^ ^]^ increasing the molecular strain as observed in Figure S49. Presumably, these interactions play an essential role in the stabilization of the *exo*‐thiepine *S*‐oxide (*exo‐* **1**) and hamper the possible interconversion to the *endo*‐analogue, as already discussed in the previous section.

**Figure 1:**
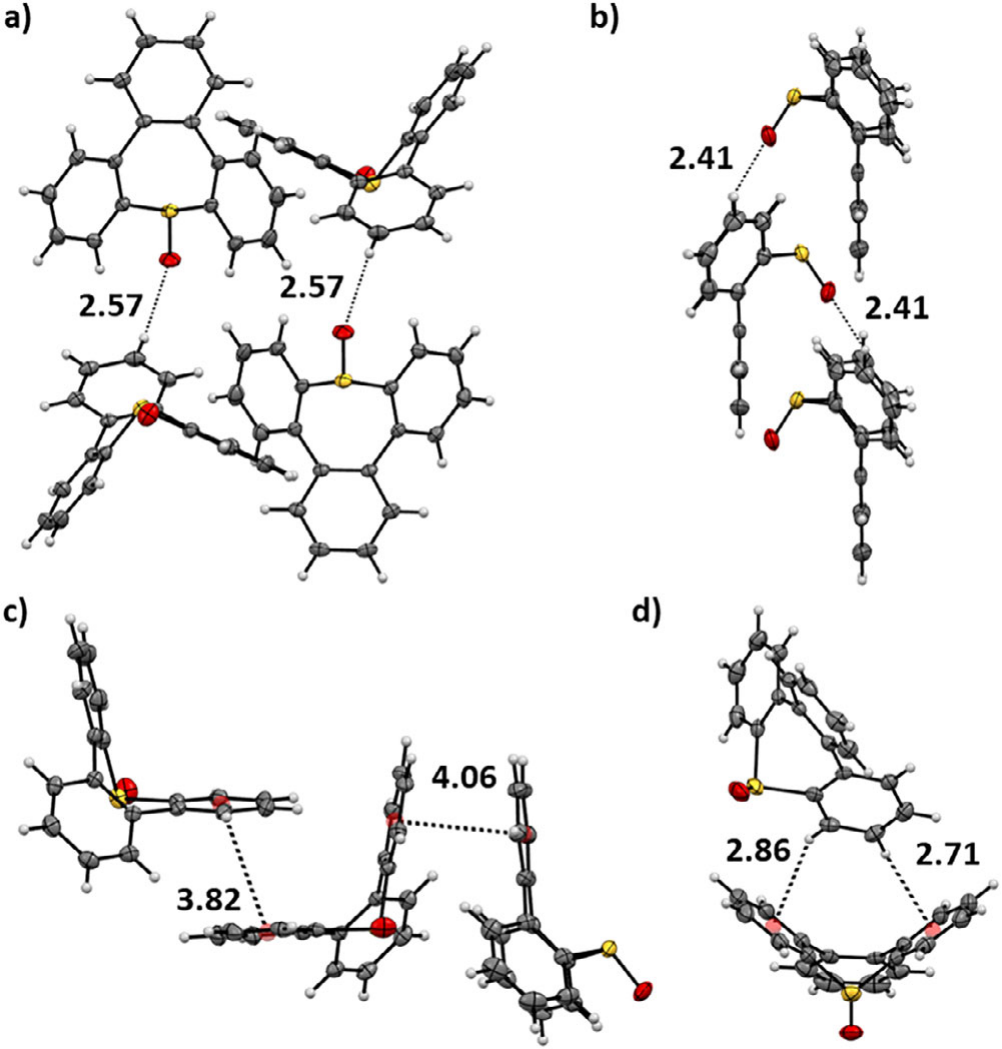
Selected short‐contact interactions in tribenzothiepine *S*‐oxides *exo*‐**1** (a, c) and *endo*‐**1** (b, d) as observed by single‐crystal X‐ray diffraction analysis. ORTEP views are represented at 50% probability, and the centroids involved in short‐contact interactions are represented by orange balls.

The careful examination of the neighboring molecules highlighted diverse intermolecular short‐contact interactions within the unit cells of tribenzothiepine derivatives (Figure 1, Figures S40, S42, S46 and S48, and Tables S4‐S7). On the one hand, in the absence of an oxygen atom, sulfide **9** displays only weak interactions such as C‐H–π and π–π stacking between the phenyl rings, despite the nonplanar character of the core. Upon oxidation of the sulfur atom, intermolecular interactions increase thanks to the presence of moderate hydrogen bonding. The sulfoxide *exo*‐**1** displays O─H bonds of approx. 2.60 Å, while the *endo*‐configuration leads to shorter O─H distances (ca. 2.41 Å), as the oxygen becomes more accessible for intermolecular interactions, resulting in an organized lamellar packing. This motif is also evident in compound **10**, wherein a higher degree of organization is observed owing to the bidirectional H‐bond interactions of the sulfone moiety.

### 2.3 Optical Characterization

**Figure 2:**
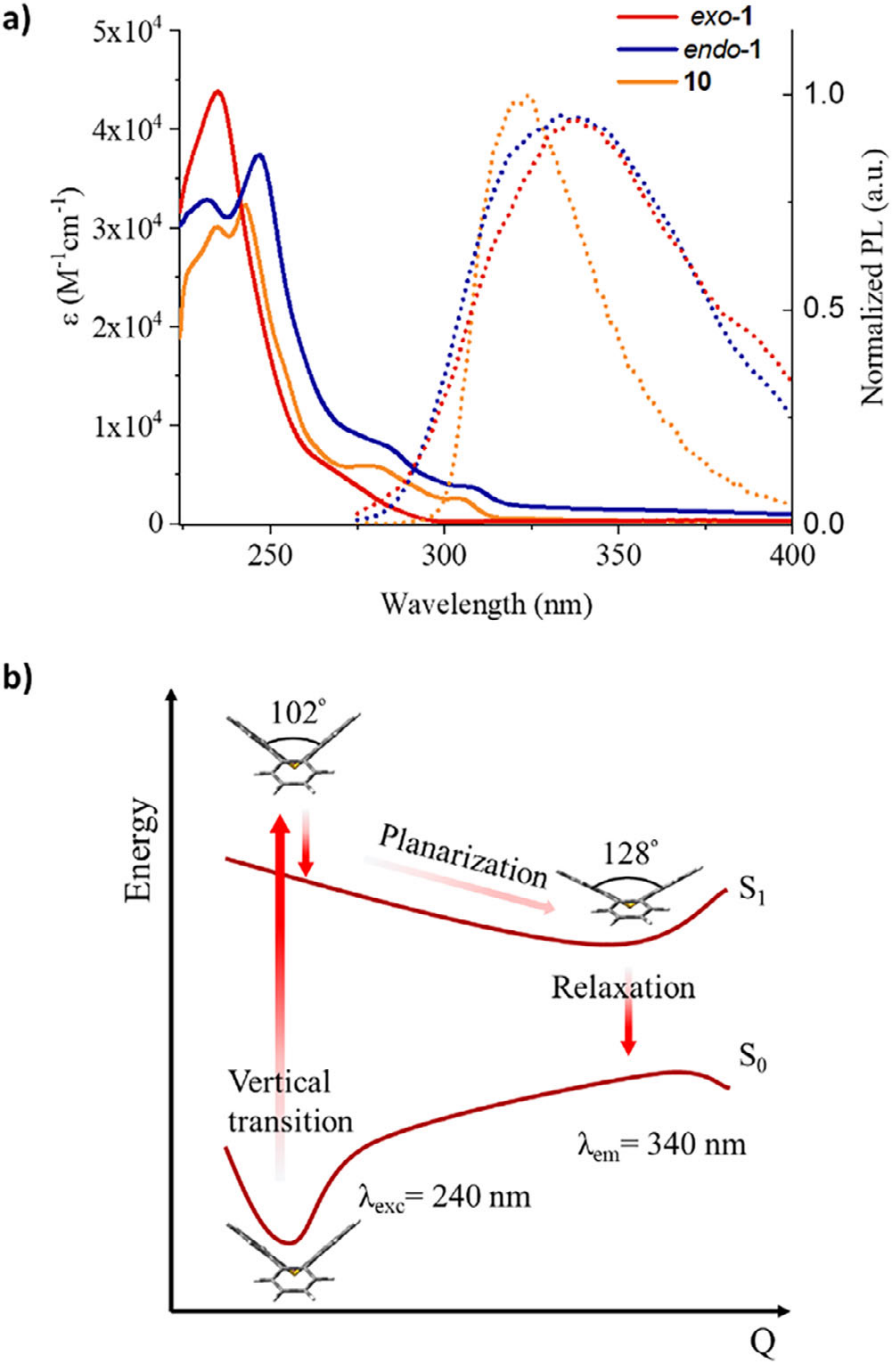
a) UV‐vis absorption (solid lines) and photoluminescence (dotted lines, λ_ex_ = 240 nm) spectra of sulfoxides *exo*‐**1** and *endo*‐**1** and sulfone **10** in CH_2_Cl_2_. b) DFT and TD‐DFT geometry optimization of *exo*‐**1** displaying the vertical transition and relaxation after excitation from the ground state. The oxygen appears hidden behind the sulfur atom in the chosen perspective view.

Prior to investigating the photochemistry of tribenzothiepine *S*‐oxide, the optical properties of the new sulfoxides (*exo*‐**1** and *endo*‐**1**) and sulfone (**10**) were studied by UV‐vis spectroscopy in CH_2_Cl_2_ solutions (Figure 2a). All compounds showed absorption bands between 200 and 350 nm, with the maxima located in the 225–250 nm region of the spectrum. In agreement with time‐dependent (TD)‐DFT calculations (Figure S33), the sulfoxide *exo‐* **1** is characterized by one absorption maximum at 234 nm, while both the analogue *endo‐* **1** and the sulfone **10** exhibit two absorption peaks located at approx. 230 nm and 245 nm. Molar extinction coefficients for the sulfoxides have values between 32 000 and 44 000 M^−1^cm^−1^, similar to the non‐cyclized *o*‐terphenyl derivatives.^[^ ^62^ ^]^ In the less energetic regime (250‐350 nm), the three compounds display redshifted shoulders, which suggests that the S_0_‐S_1_ transitions are not the most favorable. This hypothesis was corroborated by further analysis of the theoretical calculations, showing that the transitions to the first excited state are located between 270 nm and 320 nm for compounds *exo*‐**1**, *endo*‐**1**, and **10**, with oscillator strengths for these HOMO‐LUMO transitions one and two orders of magnitude weaker than the maxima (Figure S33).

When excited at 240 nm, the sulfoxides *exo‐* **1** and *endo‐* **1** present broad emission bands with maxima at approx. 340 nm and low photoluminescence intensities (Figure 2a). Both the weak emission and the strong Stokes shifts suggest a conformational reorganization in the excited states, opening the possibility of a nonradiative decay. Indeed, Ottosson et al. demonstrated through theoretical studies that heterocycles with eight π‐electrons, such as dibenzo[*b,f*]oxepines and dibenzo[*b,f*]thiepines, present a strong planarization driving force in the excited state.^[^ ^59^, ^63^ ^]^ TD‐DFT geometry optimization of the *exo* isomer of tribenzo[*b,d,f*]thiepine *S*‐oxide confirmed the planarization of the seven‐membered heterocycle in the first excited state (Figure 2b), with an angle between the two lateral phenyl groups widening from 102° in the ground state to 128° in the S_1_ excited state (Figure S34). In contrast with the reported dibenzo[*b,f*]thiepine analogues, a fully coplanar conformation is not reached for *exo*‐**1**, which is presumably due to the steric constraints at each biaryl pattern, with a torsion angle reaching 32° as a minimum (Figure S34). In contrast, sulfone **10** exhibits a more intense emission and a hypsochromic shift of approx. 20 nm with respect to the sulfoxide analogues.

### 2.4 Photochemical Reactivity

**Figure 3:**
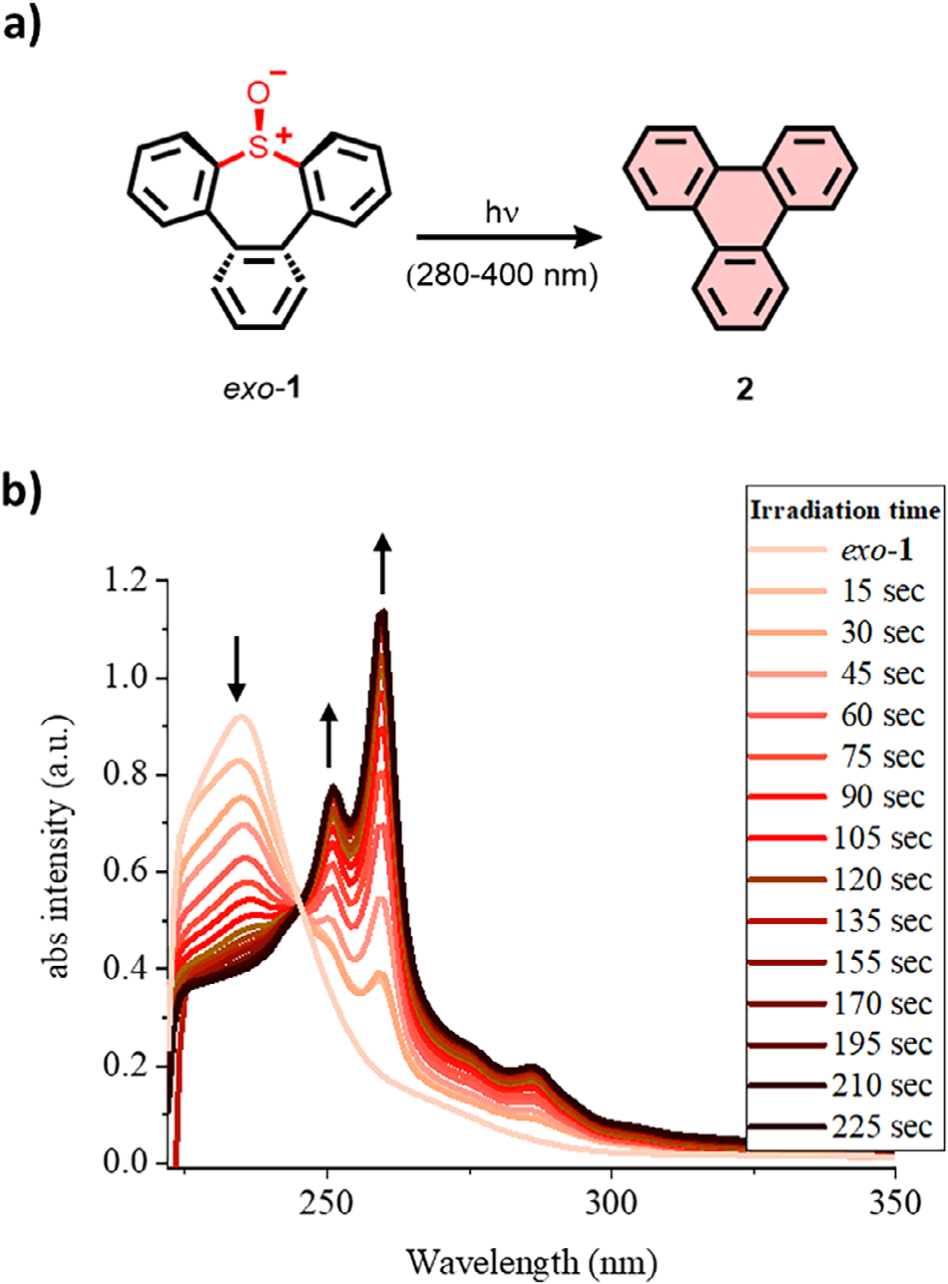
a) Photoreaction of the *exo* isomer of thiepine *S*‐oxide *exo*‐**1** to give triphenylene upon SO extrusion. b) Photoinduced extrusion of SO in CH_2_Cl_2_ solutions of *exo*‐**1** (ca. 10^−5 ^M) upon UV light irradiation (280‐400 nm), monitored by UV‐vis absorption.

First attempts to assess the photochemistry of the novel tribenzothiepine *S*‐oxides were performed by irradiating CH_2_Cl_2_ solutions of the *exo*‐isomer under UV light (280‐400 nm; see “Materials and Methods” section in the ESI). To our delight, after a few seconds of illuminating a quartz cuvette containing a diluted sample of *exo*‐**1** (10^−5 ^M), significant changes were observed in monitoring the UV‐vis absorption (Figure 3). The characteristic maximum located at 234 nm decreased rapidly in favor of two new peaks at 251 nm and 260 nm, with a clear isosbestic point at 245 nm. After approx. 3 minutes, both peaks became stable structured bands, characteristic of flat aromatic hydrocarbons. Additionally, with the naked eye we could detect the initially colorless solution turn yellow upon photoirradiation, presumably owing to the formation of elemental sulfur species. To confirm the photoinduced SO extrusion from tribenzothiepine *S*‐oxide, the final spectrum (after 225 s) was compared with the one of freshly synthesized triphenylene (**2**) (Figure S27). The perfect match of the absorption spectra corroborated the SO elimination and the concomitant ring contraction, which was subsequently characterized on a larger scale by NMR spectroscopy (Figure S25). When a sample of *exo*‐**1** in CD_2_Cl_2_ was irradiated in a quartz NMR tube, conversion was monitored on ^1^H‐NMR spectra, with the progressive disappearance of the tribenzothiepine *S*‐oxide signals while the two characteristic multiplets of **2** at 8.69 and 7.66 ppm increased. After 36 h of irradiation, a yield of 90% was obtained. However, it is important to mention that the photoinduced process is much slower in these conditions, presumably due to the limited radiant flux of our light source for a higher concentration of the reaction medium, as well as the screen effect of the generated triphenylene.

**Figure 4:**
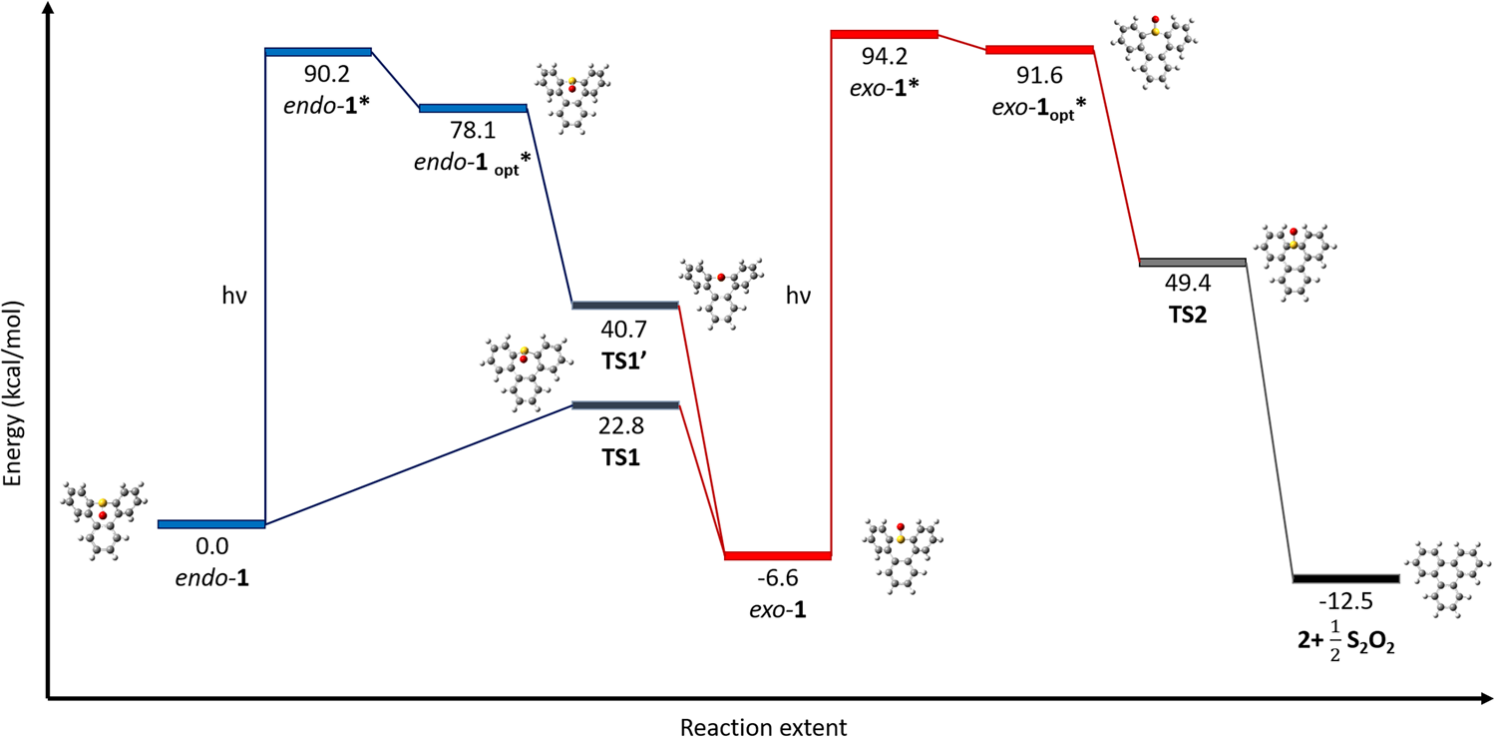
DFT and TD‐DFT mechanistic studies for the isomerization and photoinduced extrusion of SO in tribenzothiepine *S*‐oxide **1** at the PBE1PBE/6–311 + g(d,p) level of theory. In the reaction extent, the recombination of SO into S_2_O_2_ is taken into account, given the well‐known instability of the SO molecule.

To shed light on the mechanism of the photochemical reaction, DFT calculations were carried out in order to elucidate the possible coordinates. The proposed pathway starts with the light‐induced vertical transition of *exo*‐**1** from the ground state to the excited state, as is expected for a common photochemical reaction (Figure 4, red pathway). As previously mentioned, the molecule undergoes a planarization toward the fully relaxed first excited state (*exo*‐1_opt_*), which prompts, through a nonradiative relaxation to the ground state, the SO elimination and ring contraction releasing triphenylene **2**. To get better insight into the singlet or triplet nature of the relaxed first excited state, further theoretical and experimental investigations were carried out. As shown in Figure S34, the geometry optimization of *exo‐* **1** in its S_1_ and T_1_ states shows a flatter conformation for the singlet excited state, which, as previously mentioned, is essential for the photochemical reaction. The S_1_ and T_1_ states display, respectively, angles of 128° and 114° between both phenyl planes, in comparison with the 102° of the ground state S_0_. Experimentally, no significant improvement of the reaction rate was detected in the absence of oxygen, known as a triplet state scavenger. In addition, when the photochemical reaction was carried out in the presence of [Ru(bpy)_3_]^2+^ or benzophenone as triplet sensitizers,^[^ ^44^ ^]^ selective excitation under an inert atmosphere above 400 nm or at 365 nm, respectively (i.e., at wavelengths where *exo*‐**1** does not absorb), resulted in no extrusion reaction or isomerization. Therefore, the involvement of a triplet T_1_ excited state appears highly unlikely, and we hypothesize that the fully relaxed first excited state (*exo*‐1_opt_*) is singlet in nature. A cheletropic reaction then leads to the elimination of SO with the concomitant formation of triphenylene **2** upon ring closure. Eventually, it has been proposed that the extruded SO fragments are transformed by dismutation to SO_2_ and elemental sulfur,^[^ ^64^ ^]^ which could be the reason for the yellowish final solution.

**Figure 5:**
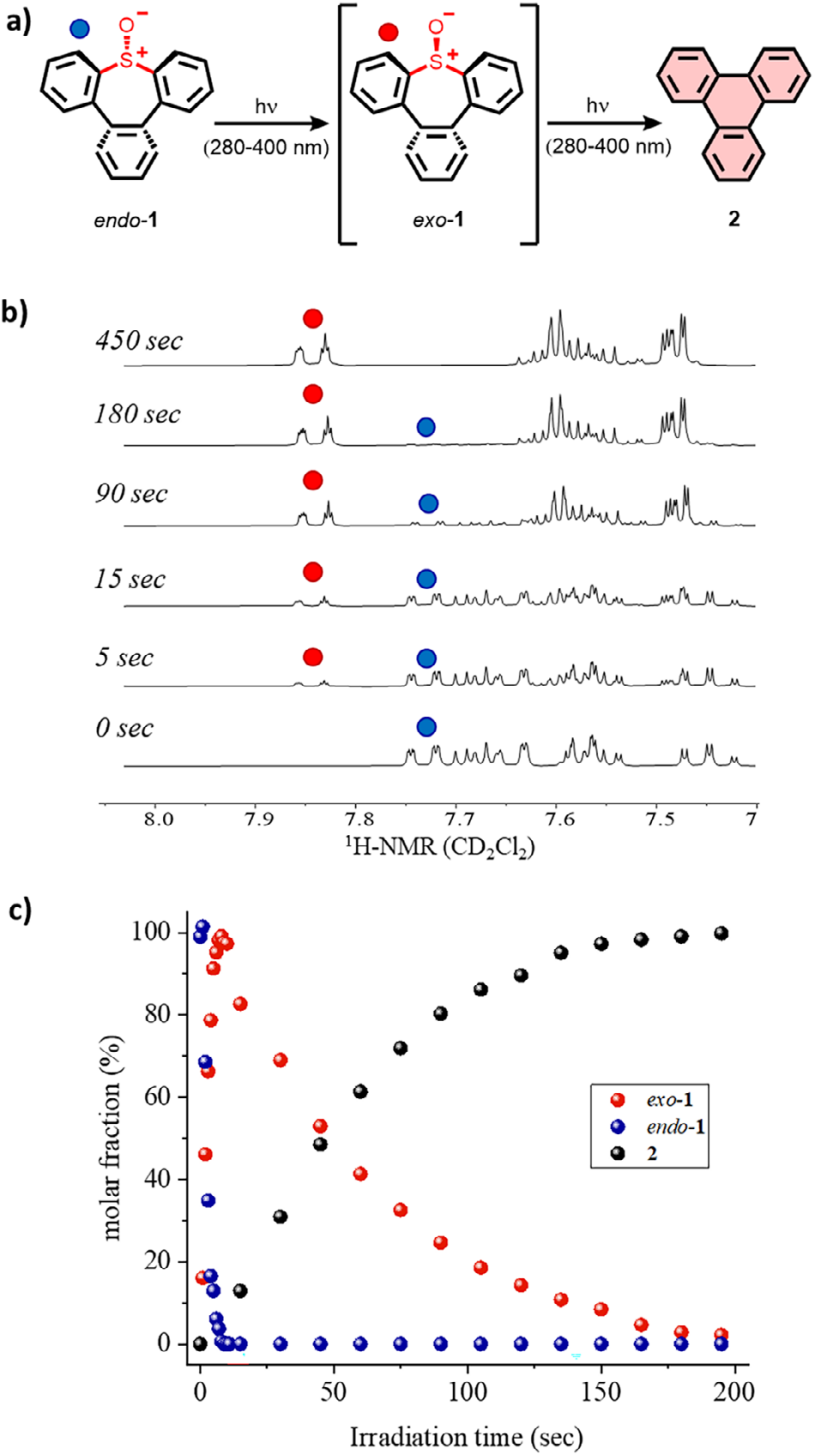
a) Photoreaction of the *endo* isomer of thiepine *S*‐oxide *endo*‐**1** to give triphenylene via a first *endo*‐*exo* photoisomerization followed by a final SO extrusion. b) Isomerization reaction of *endo*‐**1** in CD_2_Cl_2_ upon UV light irradiation (280‐400 nm), monitored by ^1^H‐NMR spectroscopy (300 MHz). The characteristic doublet of doublet of *endo*‐**1** is highlighted by a blue dot, while a red dot is used for the *exo*‐**1** stereoisomer. c) Kinetic profile for the reaction of sulfoxide *endo*‐**1** (blue dots, initial concentration of approx. 10^−5 ^M in CH_2_Cl_2_) upon photoirradiation (280‐400 nm), involving the isomerization into the *exo*‐**1** intermediate (red dots) and subsequent SO extrusion releasing triphenylene **2** (black dots).

A similar photochemical study was next carried out on the *endo*‐isomer *endo*‐**1**. Strikingly, it does not undergo a direct SO extrusion under UV light irradiation (280‐400 nm), in contrast with the *exo*‐analogue. This compound first undergoes a photoisomerization to give the more stable *exo*‐**1**, which is in turn converted into triphenylene **2**. This phenomenon was corroborated by ^1^H‐NMR and absorption spectroscopy studies (Figure 5 and Figure S28), where a quantitative isomerization of the *endo*‐derivative was observed prior to the ring contraction. From a mechanistic point of view, a thermally induced direct isomerization of *endo*‐**1** into *exo*‐**1** via the simultaneous epimerization of both biphenyl moieties (see above and Figure S32) was ruled out, since these experiments were carried out at room temperature. As mentioned above, the photochemical inversion of sulfoxide configuration has been previously reported,^[^ ^33^, ^34^, ^35^, ^36^, ^37^, ^38^, ^39^, ^40^ ^]^ involving either an *α*‐cleavage of the sulfinyl moiety or a direct epimerization via nonhomolytic sp^2^‐hybridization of the sulfur atom. In the latter case, the presence of a catalyst such as [Ru(bpy)_3_]^2+^, 2,4,6‐triphenylpyrylium tetrafluoroborate, or benzophenone was required, to allow for the formation of a radical cation through photochemical oxidation or the activation of the triplet state by intermolecular energy transfer.

In the case of tribenzothiepine *S*‐oxide *endo*‐**1**, theoretical calculations point toward an isomerization into *exo*‐**1** through a photoinduced stereoinversion of the sulfoxide moiety, involving an intermediate sp^2^‐like hybridization in the absence of carbon‐sulfur homolysis. Since this photoisomerization occurs in the absence of a photocatalyst, whether a triplet's energy transfer or a radical cation state is involved appears challenging to decipher. The geometry of the tribenzothiepine *S*‐oxide sulfur atom was evaluated at the first singlet and triplet states, and the geometry of the corresponding radical cation was also optimized (Table S1). Upon excitation to the S_1_ state, an increase of the sulfur sp^2^ character was observed as the average angle within the pyramidal moiety rises from 105° to 112°. Additionally, the dihedral angle defined as Φ is reduced from 64 to 55° en route to a higher planarization of the sulfoxide group. Conversely, both the T_1_ state and the radical cation geometry displayed the opposite trend, with an increase of the pyramidal character at the sulfur atom. Therefore, the relaxed S_1_ state seems to be essential for the photoinduced isomerization, but the full mechanistic pathway is still unclear.

Regarding the reaction kinetics, a logarithmic regression is obtained for both the photoinduced isomerization and the SO extrusion, which can thus be considered of second order or pseudo‐first order, in line with most photochemical reactions (Figure S29).^[^ ^65^ ^]^ Assuming a reaction of order two, the reaction rate coefficient of the isomerization of *endo*‐**1** into *exo*‐**1** displays a value more than five times higher than the SO extrusion, and thus, the photoconversion of the isomer *endo‐* **1** into triphenylene **2** can be considered a stepwise process. This is also in agreement with the absence of other stable intermediates, which can be confirmed by the reactant and product curves intersecting at a molar fraction of 50% (Figure 5c).

Finally, it is worthy to mention that neither tribenzothiepine **9** nor its sulfone analogue **10** presented a photochemical response in solution under light irradiation, and the photoinduced extrusion of SO was only observed on the tribenzothiepine *S*‐oxide scaffold. Thus, the photoconversion into triphenylene **2** was attempted in solid state, in anticipation of more technology‐relevant applications.

### 2.5 Solid‐state Studies

As foreseen from the thermal stability in solution of the *exo*‐sulfoxide *exo*‐**1**, no thermally induced extrusion was detected by heating samples in the solid state (powder and thin films), allowing for the deposition of intact *exo*‐**1** by vacuum sublimation (100 °C and 10^−5^ mbar). Thermal gravimetric analysis (TGA) indeed showed that the compound powder is stable up to approx. 180 °C, followed by a gradual weight loss reaching 97% at approx. 320 °C (Figure S51). Therefore, contrary to previously reported thiepine structures, this tribenzothiepine *S*‐oxide presents good thermal stability, while the late‐stage extrusion reaction can be selectively triggered in the solid state by light irradiation.

**Figure 6:**
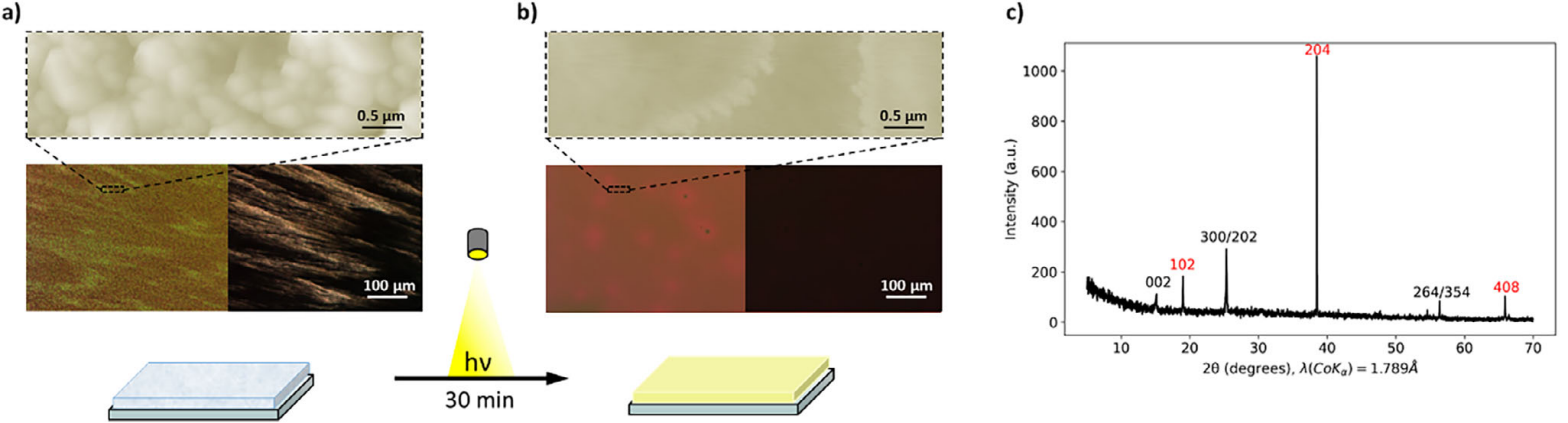
a, b) AFM (top image), optical (bottom left image), and polarized microscopy (bottom right image) micrographs of *exo*‐**1** thin films before (a) and after (b) photoirradiation. c) Out‐of‐plane X‐ray diffraction 2θ/ω pattern of an *exo*‐**1** polycrystalline thin film before irradiation. Preferential planes with direction (102) appear in red.

Thin films of the sulfoxide *exo*‐**1** were prepared by drop‐casting onto different substrates (silicon wafers, glass, and quartz; see Supporting Information, Methods Section). After evaporation of the solvent, the thin films deposited onto silicon substrates were analyzed by optical (OM) and polarized optical microscopy (POM) techniques, showing a polycrystalline distribution with a lamellar pattern indicating an anisotropic directional growth during deposition (Figure 6a). The polycrystalline character was confirmed by X‐ray diffraction of the thin film that rendered a 2θ/ω pattern characteristic of a textured film (Figure 6c). Indeed, a preferential out‐of‐plane orientation was found for the direction (102) (Figure S53), in addition to other less intense reflection planes. Upon UV light irradiation, the films suffered a color change from a shiny white to a yellowish tone, as previously observed in solution. Subsequently, under the microscope, we observed the loss of the crystalline arrangement, giving as a result an amorphous distribution characterized by a highly homogeneous thin film and the absence of response under the POM (Figure 6b). Analyzed by atomic force microscopy (AFM), initial *exo*‐**1** thin‐film micrographs displayed well‐defined grain domains characteristic of polycrystalline distributions. Conversely, after irradiation, the AFM images showed a smooth homogeneous surface in agreement with the optical microscopy. This transition was also assessed by the two states' roughness, as the root mean square (RMS) is smoothened from 111 nm for the neat sample to 12 nm after light exposure. When this phenomenon was monitored by UV‐vis absorption, we could, however, only observe small modifications of the initial spectrum landscape that did not match the triphenylene thin‐film signature (Figure S30). Increasing the light source power or the exposure time ended up in the destruction of the films.

With this in hand, we hypothesized that the photoinduced extrusion of SO takes place only on the thin‐film surface, while the thiepine derivatives of the inner layers stay unreacted due to the low penetration of UV light and the shielding effect of the upper newly formed triphenylene. To prove this, a powder sample of *exo*‐**1** was irradiated under the UV lamp until a visible white‐to‐yellow color change appeared. After dissolution of this powder in CDCl_3_, the ^1^H‐NMR spectroscopy confirmed that most of the starting material remained stable in the solid state, whereas only 2% of the powder was converted to triphenylene **2** upon photoirradiation (Figure S26). Next, to reach smaller material thickness, we assessed the photochemical elimination of SO on a water suspension of *exo*‐**1** nanoparticles, fabricated by the precipitation method.^[^ ^66^ ^]^ Monitored by UV‐visible absorption, a total conversion of the nanoparticles was confirmed by the disappearance of the thiepine characteristic band and the rise of the redshifted triphenylene structured fingerprint (Figure S31). Therefore, photochemical SO extrusion can indeed be conducted on tribenzothiepine *S*‐oxides in the solid state to give rise to the triphenylene core, provided that incident light can reach the inner molecular layers. This proof of concept opens doors to the exploitation of tribenzothiepine *S*‐oxide derivatives as soluble precursors of more complex target PAHs incorporating a triphenylene core, provided that the sulfoxide precursor presents an absorption spectrum shifted toward the visible range and/or complementary absorption bands to the generated PAH, so that light penetration is enhanced and photoconversion is improved in organic thin films.

## 3 Conclusions

This study provides a comprehensive investigation of tribenzo[*b*,*d*,*f*]thiepine *S*‐oxide and its photoconversion as a model system toward the elaboration of molecular materials structurally derived from triphenylene. Tribenzo[*b*,*d*,*f*]thiepine *S*‐oxide was prepared in 68% overall yield over 4 steps via a robust synthetic route relying on the parent thiepine as a key intermediate. After careful optimization, oxidation was achieved under fully chemoselective conditions to give the target sulfoxide, with selective access to either the *exo* or *endo* stereoisomer controlled by the nature of the boron‐based additive. Structural analysis highlighted the influence of the sulfinyl moiety's orientation on the relative stability of these compounds, while experimental and theoretical investigations revealed the thermal and photochemical pathways for isomerization from the *endo* to the most stable *exo* counterpart.

The light‐induced SO extrusion process was first studied in solution. Detailed spectroscopic investigations complemented by theoretical studies deciphered the photochemical behavior of tribenzo[*b*,*d*,*f*]thiepine *S*‐oxide as a function of its geometry, demonstrating that the *exo* isomer undergoes efficient SO elimination under UV light to give triphenylene in yields up to 90%, in contrast with the *endo* isomer, which first requires a photoisomerization to its *exo* counterpart. Finally, the photoconversion of tribenzothiepine *S*‐oxide was successfully achieved in the solid state. Indeed, nanoparticles of the *exo* isomer were quantitatively transformed into triphenylene, but the extent of the SO extrusion process in powder and thin‐film samples remains hampered by low light penetration. To optimize this parameter, work is currently underway to rationally design tribenzothiepine *S*‐oxide derivatives with optimal molecular absorption properties.

Overall, this work opens doors to the exploitation of extended tribenzothiepine *S*‐oxide derivatives as soluble precursors of complex target PAHs incorporating a triphenylene core, thus offering a greener and selective route to triphenylene‐based materials with promising applications.^[^ ^67^, ^68^ ^]^

## Supporting Information

The data that support the findings of this study are available in the Electronic Supplementary Information (ESI), including experimental procedures, characterization of compounds, reactivity studies, theoretical calculations, and solid‐state characterization. Crystallographic data for compounds *exo*‐**1**, *endo*‐**1**, **5**, **9** and **10** have been deposited at The Cambridge Crystallographic Data Centre.^[^ ^69^ ^]^ Additional references cited within the Supporting Information.^[^ ^70^, ^71^, ^72^, ^73^, ^74^, ^75^, ^76^, ^77^, ^78^, ^79^, ^80^, ^81^, ^82^, ^83^, ^84^ ^]^

## Conflict of Interest

The authors declare no conflicts of interest.

## Supporting information



Supporting Information

Supporting Information

Supporting Information

## Data Availability

The data that support the findings of this study are available in the supplementary material of this article.

## References

[1] H. Yamada , D. Kuzuhara , M. Suzuki , H. Hayashi , N. Aratani , Bull. Chem. Soc. Jpn. 2020, 93, 1234.

[2] E. Jeong , T. Ito , K. Takahashi , T. Koganezawa , H. Hayashi , N. Aratani , M. Suzuki , H. Yamada , ACS Appl. Mater. Interfaces 2022, 14, 32319. 35816704 10.1021/acsami.2c07313 PMC9307050

[3] R. R. Zope , M. Olguin , T. Baruah , J. Chem. Phys. 2012, 137, 084317. 22938243 10.1063/1.4739272

[4] P. W. Zach , S. A. Freunberger , I. Klimant , S. M. Borisov , ACS Appl. Mater. Interfaces 2017, 9, 38008. 29016109 10.1021/acsami.7b10669

[5] T. Okujima , Y. Hashimoto , G. Jin , H. Yamada , H. Uno , N. Ono , Tetrahedron 2008, 64, 2405.

[6] H. Hayashi , H. Yamada , Chem. Sci. 2025, 16, 11204. 40474956 10.1039/d5sc02422f PMC12135676

[7] A. R. Brown , A. Pomp , D. M. De Leeuw , D. B. M. Klaassen , E. E. Havinga , P. Herwig , K. Müllen , J. Appl. Phys. 1996, 79, 2136.

[8] P. T. Herwig , K. Müllen , Adv. Mater. 1999, 11, 480.

[9] N. Vets , M. Smet , W. Dehaen , Tetrahedron Lett. 2004, 45, 7287.

[10] T.‐H. Chuang , H.‐H. Hsieh , C.‐K. Chen , C.‐C. Wu , C.‐C. Lin , P.‐T. Chou , T.‐H. Chao , T. J. Chow , Org. Lett. 2008, 10, 2869. 18510329 10.1021/ol8010419

[11] H. Yamada , Y. Yamashita , M. Kikuchi , H. Watanabe , T. Okujima , H. Uno , T. Ogawa , K. Ohara , N. Ono , Chem. Eur. J. 2005, 11, 6212. 16075447 10.1002/chem.200500564

[12] H. Yamada , T. Okujima , N. Ono , Chem. Commun. 2008, 26, 2957. 10.1039/b719964c 18688321

[13] L. Lerena , R. Zuzak , S. Godlewski , A. M. Echavarren , Chem. Eur. J. 2024, 30, e202402122. 39077888 10.1002/chem.202402122

[14] A. Jančařík , J. Holec , Y. Nagata , M. Šámal , A. Gourdon , Nat. Commun. 2022, 13, 223. 35017480 10.1038/s41467-021-27809-0 PMC8752783

[15] M. Watanabe , Y. J. Chang , S.‐W. Liu , T.‐H. Chao , K. Goto , M. M. Islam , C.‐H. Yuan , Y.‐T. Tao , T. Shinmyozu , T. J. Chow , Nat. Chem. 2012, 4, 574. 22717444 10.1038/nchem.1381

[16] A. Jancarik , G. Levet , A. Gourdon , Chem. Eur. J. 2019, 25, 2366. 30508267 10.1002/chem.201805975

[17] R. Mondal , R. M. Adhikari , B. K. Shah , D. C. Neckers , Org. Lett. 2007, 9, 2505. 17516652 10.1021/ol0709376

[18] R. Mondal , C. Tönshoff , D. Khon , D. C. Neckers , H. F. Bettinger , J. Am. Chem. Soc. 2009, 131, 14281. 19757812 10.1021/ja901841c

[19] C. Tönshoff , H. F. Bettinger , Angew. Chem. Int. Ed. 2010, 49, 4125. 10.1002/anie.200906355 20432492

[20] B. Shen , J. Tatchen , E. Sanchez‐Garcia , H. F. Bettinger , Angew. Chem. Int. Ed. 2018, 57, 10506. 10.1002/anie.201802197 29737606

[21] T. Miyazaki , M. Watanabe , T. Matsushima , C. Chien , C. Adachi , S. Sun , H. Furuta , T. J. Chow , Chem. Eur. J. 2021, 27, 10677. 33904186 10.1002/chem.202100936

[22] K. Eimre , J. I. Urgel , H. Hayashi , M. Di Giovannantonio , P. Ruffieux , S. Sato , S. Otomo , Y. S. Chan , N. Aratani , D. Passerone , O. Gröning , H. Yamada , R. Fasel , C. A. Pignedoli , Nat. Commun. 2022, 13, 511. 35082284 10.1038/s41467-022-27961-1 PMC8791976

[23] J. Besteiro‐Sáez , L. M. Mateo , S. Salaverría , T. Wang , P. Angulo‐Portugal , J. P. Calupitan , J. Rodríguez‐Fernández , A. García‐Fuente , J. Ferrer , D. Pérez , M. Corso , D. G. De Oteyza , D. Peña , Angew. Chem. Int. Ed. 2024, 63, e202411861. 10.1002/anie.202411861 39110601

[24] T. Kitao , T. Miura , R. Nakayama , Y. Tsutsui , Y. S. Chan , H. Hayashi , H. Yamada , S. Seki , T. Hitosugi , T. Uemura , Nat. Synth. 2023, 2, 848.

[25] J. I. Urgel , H. Hayashi , M. Di Giovannantonio , C. A. Pignedoli , S. Mishra , O. Deniz , M. Yamashita , T. Dienel , P. Ruffieux , H. Yamada , R. Fasel , J. Am. Chem. Soc. 2017, 139, 11658. 28780869 10.1021/jacs.7b05192

[26] J. I. Urgel , S. Mishra , H. Hayashi , J. Wilhelm , C. A. Pignedoli , M. Di Giovannantonio , R. Widmer , M. Yamashita , N. Hieda , P. Ruffieux , H. Yamada , R. Fasel , Nat. Commun. 2019, 10, 861. 30787280 10.1038/s41467-019-08650-y PMC6382834

[27] Z. Ruan , J. Schramm , J. B. Bauer , T. Naumann , H. F. Bettinger , R. Tonner‐Zech , J. M. Gottfried , J. Am. Chem. Soc. 2024, 146, 3700. 38216144 10.1021/jacs.3c09392 PMC10870776

[28] J. Krüger , F. García , F. Eisenhut , D. Skidin , J. M. Alonso , E. Guitián , D. Pérez , G. Cuniberti , F. Moresco , D. Peña , Angew. Chem. Int. Ed. 2017, 56, 11945. 10.1002/anie.201706156 28771920

[29] J. Han , X. Liu , Y. Li , Z. Lou , M. Yi , H. Kong , J. Luo , Org. Chem. Front. 2019, 6, 2839.

[30] R. Zuzak , M. Kumar , O. Stoica , D. Soler‐Polo , J. Brabec , K. Pernal , L. Veis , R. Blieck , A. M. Echavarren , P. Jelinek , S. Godlewski , Angew. Chem. Int. Ed. 2024, 63, e202317091. 10.1002/anie.202317091 38192200

[31] A. Okba , P. Simón Marqués , K. Matsuo , N. Aratani , H. Yamada , G. Rapenne , C. Kammerer , Beilstein J. Org. Chem. 2024, 20, 287. 38379731 10.3762/bjoc.20.30 PMC10877077

[32] A. Jančařík , D. Mildner , Y. Nagata , M. Banasiewicz , J. Olas , B. Kozankiewicz , J. Holec , A. Gourdon , Chem. Eur. J. 2021, 27, 12388. 34101270 10.1002/chem.202101577

[33] C. Aurisicchio , E. Baciocchi , M. F. Gerini , O. Lanzalunga , Org. Lett. 2007, 9, 1939. 17439134 10.1021/ol070500y

[34] K. Makino , K. Tozawa , Y. Tanaka , A. Inagaki , H. Tabata , T. Oshitari , H. Natsugari , H. Takahashi , J. Org. Chem. 2021, 86, 17249. 34806388 10.1021/acs.joc.1c02320 PMC8650104

[35] B. W. Vos , W. S. Jenks , J. Am. Chem. Soc. 2002, 124, 2544. 11890804 10.1021/ja017228m

[36] W. S. Jenks , Spectrum 2001, 14, 1.

[37] K. Mislow , M. Axelrod , D. R. Rayner , H. Gotthardt , L. M. Coyne , G. S. Hammond , J. Am. Chem. Soc. 1965, 87, 4958.

[38] G. S. Hammond , R. S. Cooke , J. Am. Chem. Soc. 1969, 86, 2739.

[39] K. Tozawa , K. Makino , Y. Tanaka , K. Nakamura , A. Inagaki , H. Tabata , T. Oshitari , H. Natsugari , N. Kuroda , K. Kanemaru , Y. Oda , H. Takahashi , J. Org. Chem. 2023, 88, 6955. 37155937 10.1021/acs.joc.3c00265 PMC10242752

[40] L. Wimberger , T. Kratz , T. Bach , Synthesis 2019, 51, 4417.

[41] S. M. Omlid , S. A. Dergunov , A. Isor , K. L. Sulkowski , J. T. Petroff , E. Pinkhassik , R. D. McCulla , Chem. Commun. 2019, 55, 1706. 10.1039/c8cc06715e 30556067

[42] M. Nag , W. S. Jenks , J. Org. Chem. 2005, 70, 3458. 15844978 10.1021/jo047995e

[43] M. Hankache , V. Magné , E. Geagea , P. Simón Marqués , S. Clair , L. Giovanelli , C. Loppacher , E. Fodeke , S. Mallet‐Ladeira , E. Maerten , C. Kammerer , D. Madec , L. Nony , Nat. Commun. 2025, 16, 4841. 40413173 10.1038/s41467-025-60075-y PMC12103517

[44] P. R. Christensen , B. O. Patrick , É. Caron , M. O. Wolf , Angew. Chem. Int. Ed. 2013, 52, 12946. 10.1002/anie.201306236 24281886

[45] M. Murata , S. Maeda , Y. Morinaka , Y. Murata , K. Komatsu , J. Am. Chem. Soc. 2008, 130, 15800. 18959401 10.1021/ja8076846

[46] C. Song , T. M. Swager , J. Org. Chem. 2010, 75, 999. 19911841 10.1021/jo902079j

[47] B. Ming , C. Yan , S. Xie , S. Liu , Y. Ren , H. Zong , W. Chen , G. Zhou , Chin. J. Chem. 2023, 41, 13.

[48] R. Li , B. Ma , S. Li , C. Lu , P. An , Chem. Sci. 2023, 14, 8905. 37621425 10.1039/d3sc02595k PMC10445433

[49] Q. Gong , X. Zhang , W. Li , X. Guo , Q. Wu , C. Yu , L. Jiao , Y. Xiao , E. Hao , J. Am. Chem. Soc. 2022, 48, 21992. 10.1021/jacs.2c08947 36414278

[50] S. Hayakawa , K. Matsuo , H. Yamada , N. Fukui , H. Shinokubo , J. Am. Chem. Soc. 2020, 142, 11663. 32543842 10.1021/jacs.0c04096

[51] Y. Tanaka , K. Matsuo , H. Yamada , N. Fukui , H. Shinokubo , Eur. J. Org. Chem. 2022, 2022, e202200770.

[52] I. Pozo , E. Guitián , D. Pérez , D. Peña , Acc. Chem. Res. 2019, 52, 2472. 31411855 10.1021/acs.accounts.9b00269

[53] M. Feofanov , V. Akhmetov , R. Takayama , K. Y. Amsharov , J. Org. Chem. 2021, 86, 14759. 34672595 10.1021/acs.joc.1c01565

[54] T. Igarashi , M. Tobisu , N. Chatani , Angew. Chem. Int. Ed. 2017, 56, 2069. 10.1002/anie.201612535 28111913

[55] L. Niu , H. Yang , Y. Jiang , H. Fu , Adv. Synth. Catal. 2013, 355, 3625.

[56] E. Dimitrijević , M. Cusimano , M. S. Taylor , Org. Biomol. Chem. 2014, 12, 1391. 24473678 10.1039/c3ob42065e

[57] R. P. Greenhalgh , Synlett 1992, 3, 235.

[58] M. Šiaučiulis , N. Ahlsten , A. P. Pulis , D. J. Procter , Angew. Chem. Int. Ed. 2019, 58, 8779. 10.1002/anie.201902903 30964596

[59] J. Toldo , O. El Bakouri , M. Solà , P. Norrby , H. Ottosson , ChemPlusChem 2019, 84, 712. 31944021 10.1002/cplu.201900066

[60] W. Zhang , S. Tao , H. Ge , Q. Li , Z. Ai , X. Li , B. Zhang , F. Sun , X. Xu , Y. Du , Org. Lett. 2020, 22, 448. 31894988 10.1021/acs.orglett.9b04206

[61] F. Naso , C. Cardellicchio , M. A. M. Capozzi , F. Capitelli , V. Bertolasi , New J. Chem. 2006, 30, 1782.

[62] B. T. King , J. Kroulík , C. R. Robertson , P. Rempala , C. L. Hilton , J. D. Korinek , L. M. Gortari , J. Org. Chem. 2007, 72, 2279. 17326684 10.1021/jo061515x

[63] R. Papadakis , H. Ottosson , Chem. Soc. Rev. 2015, 44, 6472. 25960203 10.1039/c5cs00057b

[64] C. Prior , R. S. Grainger , In Progress in Heterocyclic Chemistry *, Vol*. 34 (Eds.: G. Gribble , R. A. Aitken ), Elsevier, 2023, pp. 1–34.

[65] M. Hippler , J. Chem. Educ. 2003, 80, 1074.

[66] N. Fabre , T. Fukaminato , I. Ikariko , L. Chocron , A. Brosseau , R. Métivier , Adv. Opt. Mater. 2024, 12, 2400452.

[67] K. Togashi , S. Nomura , N. Yokoyama , T. Yasuda , C. Adachi , J. Mater. Chem. 2012, 22, 20689.

[68] E. C. Rüdiger , M. Müller , S. Koser , F. Rominger , J. Freudenberg , U. H. F. Bunz , Chem. Eur. J. 2018, 24, 1036. 28976041 10.1002/chem.201704103

[69] Deposition Numbers CCDC‐ 2463548 (for *exo*‐**1**), CCDC‐2463549 (for *endo*‐**1**), CCDC‐2463550 (for **5**), CCDC‐2463551 (for **9**), and CCDC‐2463552 (for **10**) contain the supplementary crystallographic data for this paper. These data are provided free of charge by the joint Cambridge Crystallographic Data Centre and Fachinformationszentrum Karlsruhe Access Structures service.

[70] S. S. Zalesskiy , V. P. Ananikov , Organometallics 2012, 31, 2302.

[71] B. A. I. SADABS, Bruker, USA, 2008.

[72] G. M. Sheldrick , Acta Crystallogr. Found. Adv. 2015, 71, 3. 10.1107/S2053273314026370 PMC4283466 25537383

[73] G. M. Sheldrick , Acta Crystallogr. C Struct. Chem. 2015, 71, 3. 25567568 10.1107/S2053229614024218 PMC4294323

[74] M. Shimizu , Y. Tomioka , I. Nagao , T. Hiyama , Synlett 2009, 19, 3147.

[75] M. Feofanov , V. Akmetov , R. Takayama , K. Amsharov , Angew. Chem. Int. Ed. 2021, 60, 5199. 10.1002/anie.202007427 PMC7986400 32924244

[76] E. Dimitrijević , M. S. Taylor , Chem. Sci. 2013, 4, 3298.

[77] K. E. Yamada , I. A. Stepek , W. Matsuoka , H. Ito , K. Itami , Angew. Chem. Int. Ed. 2023, 62, e202311770. 10.1002/anie.202311770 37902441

[78] Gaussian 16, M. J. Frisch , G. W. Trucks , H. B. Schlegel , G. E. Scuseria , M. A. Robb , J. R. Cheeseman , G. Scalmani , V. Barone , G. A. Petersson , H. Nakatsuji , X. Li , M. Caricato , A. V. Marenich , J. Bloino , B. G. Janesko , R. Gomperts , B. Mennucci , H. P. Hratchian , J. V. Ortiz , A. F. Izmaylov , J. L. Sonnenberg , D. Williams‐Young , F. Ding , F. Lipparini , F. Egidi , J. Goings , B. Peng , A. Petrone , T. Henderson , D. Ranasinghe , V. G. Zakrzewski , J. Gao , N. Rega , G. Zheng , W. Liang , M. Hada , M. Ehara , K. Toyota , R. Fukuda , J. Hasegawa , M. Ishida , T. Nakajima , Y. Honda , O. Kitao , H. Nakai , T. Vreven , K. Throssell , J. A. Montgomery Jr. , J. E. Peralta , F. Ogliaro , M. J. Bearpark , J. J. Heyd , E. N. Brothers , K. N. Kudin , V. N. Staroverov , T. A. Keith , R. Kobayashi , J. Normand , K. Raghavachari , A. P. Rendell , J. C. Burant , S. S. Iyengar , J. Tomasi , M. Cossi , J. M. Millam , M. Klene , C. Adamo , R. Cammi , J. W. Ochterski , R. L. Martin , K. Morokuma , O. Farkas , J. B. Foresman , D. J. Fox , Gaussian, Inc., Wallingford CT, 2016.

[79] M. W. Lodewyk , C. Soldi , P. B. Jones , M. M. Olmstead , J. Rita , J. T. Shaw , D. J. Tantillo , J. Am. Chem. Soc. 2012, 134, 18550. 23101682 10.1021/ja3089394

[80] M. W. Lodewyk , M. R. Siebert , D. J. Tantillo , Chem. Rev. 2012, 112, 1839. 22091891 10.1021/cr200106v

[81] T. Cheshire , P. Ramblenm , D. J. Tantillo , M. R. Siebert , M. W. Lodewyk , CHEmical SHift REpository with Coupling Constants Added Too, http://cheshirenmr.info.

[82] T. Lu , F. Chen , J. Comput. Chem. 2012, 33, 580. 22162017 10.1002/jcc.22885

[83] T. Lu , J. Chem. Phys. 2024, 161, 082503. 39189657 10.1063/5.0216272

[84] C. F. Macrae , I. Sovago , S. J. Cottrell , P. T. A. Galek , P. McCabe , E. Pidcock , M. Platings , G. P. Shields , J. S. Stevens , M. Towler , P. A. Wood , J. Appl. Crystallogr. 2020, 53, 226. 32047413 10.1107/S1600576719014092 PMC6998782

