# Supplementary material for: Light‐Induced SO Extrusion from Tribenzothiepine S‐oxides: A Precursor Approach to the Triphenylene Core
Source: Chemistry. 2025 Nov 7;31(70):e02655. doi: 10.1002/chem.202502655 (PMC12712768; doi:10.1002/chem.202502655)
Supplement: Supplementary file 1 — Supporting Information [file CHEM-31-e02655-s003.pdf]

Light-Induced SO Extrusion from Tribenzothiepine *S*-oxides: a Precursor Approach to the Triphenylene Core

Pablo Simón Marqués,[a] Aissam Okba,[a,b] Nicolas Bréfuel,[a] So Ueno,[c] Nathalie Saffon-Merceron,[d] Nicolas Ratel-Ramond,[e] Kyohei Matsuo,[b,c] Gwénaël Rapenne,[a,b] Naoki Aratani,[b] Claire Kammerer*[a] and Hiroko Yamada*[c]

[a]CEMES, Université de Toulouse, CNRS, 29 rue Marvig, 31055 Toulouse, France
[b]Division of Materials Science, Nara Institute of Science and Technology, NAIST, 8916-5 Takayama-cho, Ikoma, Nara 630-0192, Japan
[c]Institute for Chemical Research, Kyoto University, Gokasho, Uji, Kyoto 611-0011, Japan
[d]Université de Toulouse, Institut de Chimie de Toulouse, ICT UAR 2599, 118 route de Narbonne, 31062 Toulouse, France
[e]Université de Toulouse, LPCNO, INSA-UPS-CNRS, 135 avenue de Rangueil, 31077 Toulouse, France

Supporting information

1. Materials and Methods S2
  1.1 Chemicals S2
  1.2 Methods S2
2. Synthetic methods S3
  2.1 Synthesis of tribenzo[*b*,*d*,*f*]thiepine **9** S3
  2.2 General procedure for the oxidation of tribenzothiepine **9** with *m*CPBA S5
3. Mass spectrometry S6
4. NMR spectroscopy S7
  4.1 Characterisation of synthetic intermediates, thiepine **9** and thiepine *S*,*S*-dioxide **10** S7
  4.2 Characterisation of thiepine *S*-oxide *exo*-**1** S10
  4.3 Characterisation of thiepine *S*-oxide *endo*-**1** S13
5. Electrochemical characterisation S16
6. Reactivity studies S17
  6.1 Investigations by NMR spectroscopy S17
    Thermal reactivity S17
    Photoreactivity S19
  6.2 Investigations by UV-vis absorption spectroscopy S20
7. Theoretical calculations S23
8. Solid-state characterisation S27
  8.1 Single crystal X-ray diffraction S27
    Structure Tables S28
    exo-tribenzo[*b*,*d*,*f*]thiepine *S*-oxide (*exo*-**1**) S32
    endo-tribenzo[*b*,*d*,*f*]thiepine *S*-oxide (*endo*-**1**) S33
    2,2''-difluoro-1,1':2',1''-terphenyl (**5**) S34
    tribenzo[*b*,*d*,*f*]thiepine (**9**) S35
    tribenzo[*b*,*d*,*f*]thiepine *S*,*S*-dioxide (**10**) S36
  8.2 Thermogravimetric analysis S38
  8.3 Optical and polarised optical microscopy S38
  8.4 Thin-films and powder X-ray diffraction S39
9. References S40

1. Materials and Methods

1.1 Chemicals

All chemicals listed herein were used as received without further purification if not indicated: 1,2-Diiodobenzene was purchased from Apollo Scientific. Tris(pentafluorophenyl)borane was purchased from BLDPharm. 4,4,5,5-Tetramethyl-1,3,2-dioxaborolane and tri-*tert*-butylphosphonium tetrafluoroborate were purchased from TCI chemicals. 1-Bromo-2-fluorobenzene, diphenylsulfane, bis(2-bromophenyl)sulfane, *m*CPBA (≤77wt%), Oxone®, $BF_3.OEt_2$, *n*-BuLi (2.5 M in hexanes), anhydrous Na_2S, K_2CO_3, Cs_2CO_3, Na_2SO_4, NEt_3, TMEDA, HFIP, SPhos, AgTFA, $Pd(MeCN)_2Cl_2$ and $Pd_2(dba)_3$ were purchased from Merck. Tetrabutylammonium bromide (TBAB) was purchased from Acros. Trimethyl borate was purchased from Alfa Aesar. $Pd_2(dba)_3$ was repurified from commercially available sources as described by Ananikov *et al*.[1] Solvents were purchased from Merck.

1.2 Methods

Reactions and purifications: Reactions under inert atmosphere were carried out using standard Schlenk techniques. Microwave reactions were performed in a CEM Discover LabMate cavity using 10 mL tubes, sealed with a PTFE cap. Flash chromatography was performed using an ARMEN-Interchim Glider Flash Spot 2 system and silica gel columns of 4, 12, 24 and 40 g of RediSep and puriFlash. Flexible plates ALUGRAM® Xtra SIL G UV254 from MACHEREY-NAGEL were used for TLC, with visualisation effected using a UV lamp (254, 366 nm).

Analysis: ^1H and ^{13}C-NMR spectra were recorded on Bruker Avance III HD 500 MHz (cryoprobe Prodigy 5mm BBO, 1H ATMA), Avance 500 MHz (cryoprobe 5mm 1H, 13C) and Avance NEO 300 MHz (probe 5mm BBFO ATM) spectrometers. Chemical shifts (δ) are reported in ppm. Coupling constants (J) are given in Hz and the following abbreviations have been used to describe the signals: singlet (s); doublet (d); triplet (t); multiplet (m). Assignments of ^1H and ^{13}C NMR spectra were made with the assistance of HMBC, HSQC and COSY spectra, as well as DFT NMR calculations.
High-resolution mass spectra (HR-MS) were performed with a Waters Xevo G2 QTof spectrometer for electrospray ionisation (ESI).
UV–Vis spectra were recorded on a Varian Cary 5000 spectrometer and fluorescence spectra on a Hitachi F-4500 fluorescence spectrophotometer.
Cyclic voltammetry was performed using a AUTOLAB PGSTAT204 potentiostat with positive feedback compensation in 0.10 M Bu_4NPF_6/CH_2Cl_2 (HPLC grade). Experiments were carried out under Ar atmosphere in a one-compartment cell equipped with a platinum working electrode (2 mm of diameter) and a platinum wire counter electrode. A saturated calomel electrode (SCE) was used as reference electrode and the potentials were calibrated using the ferrocene/ferrocenium couple (Fc/Fc+) whose redox potential was measured before and after each experiment.
Thermogravimetric analysis (TGA) was carried out using an ATG/DSC3+ (Mettler-Toledo).
Atomic force microscopy (AFM) images were obtained using Shimadzu SPM-9700 in the tapping mode.
Polarised optical microscopy images of the thin films were obtained using a Zeiss Axio Scope.A1 microscope using an objective lens (10 ×).

Photoirradiation: Photochemical experiments were carried out in quartz vessels using a HAMAMATSU UV Spot Light Source Lamp (L9588-06A) with a light guide and filter allowing 280-400 nm wavelengths. Light power and distance were adjusted to obtain an irradiation of 120 mW/cm^2.

Thin-film preparation: Organic thin-films of compound *exo*-**1** were prepared by drop-casting from toluene solutions (2 mg / mL) onto highly n-doped silicon wafers with a 300 nm-thick thermally grown SiO_2 layer, glass and quartz. The silicon substrates were previously cleaned with deionised water, acetone and 2-propanol for 10 min in an ultrasonic bath. The substrates drawn in 2-propanol were heated

to 100 °C using a hotplate and exposed to 2-propanol vapour to clean the surface when the substrates were removed. Then, they were dried using argon gas and cleaned under the exposure of UV and O_3 using UV−O_3 cleaner (UV253V8, Filgen) for 30 min.

Single crystal X-ray diffraction: Crystallographic data were collected at 193(2) K using Mo Kα radiation (λ=0.71073 Å) on a Bruker-AXS Kappa APEX II Quazar diffractometer equipped with a 30W air-cooled microfocus source (*endo*-**1**, **5** and **9**) and on a Bruker AXS D8-Venture diffractometer equipped with a Mo Kα sealed tube, a multilayer TRIUMPH X-ray mirror, a Photon III-C14 detector (*exo*-**1** and **10**). Phi- and omega-scans were used. Space group was determined on the basis of systematic absences and intensity statistics. Semi-empirical absorption correction was employed.[2] These structures were solved using an intrinsic phasing method (SHELXT),[3] and refined using the least-squares method on F^2.[4] All non-H atoms were refined with anisotropic displacement parameters. Hydrogen atoms were refined isotropically at calculated positions using a riding model with their isotropic displacement parameters constrained to be equal to 1.2 times for all aromatic carbon atoms. Short-contact distances are given in Å.
CCDC-2463548 (*exo*-**1**), CCDC-2463549 (*endo*-**1**), CCDC-2463550 (**5**), CCDC-2463551 (**9**) and CCDC-2463552 (**10**) contain the supplementary crystallographic data for this paper. These data can be obtained free of charge from The Cambridge Crystallographic Data Centre via www.ccdc.cam.a-c.uk/data_request/cif.

Thin-films X-ray diffraction: Data for X-ray diffraction (XRD) analysis were collected on a Panalytical Empyrean diffractometer, using the Kα radiation produced by a Co source (λ = 1.789 Å), equipped with programmable divergence slits and an X'Celerator 1D detector. Diffraction peak indexing was performed based on simulated diffraction profiles using the atomic coordinates determined in this study. However, as measurements temperatures differ between single crystal and thin-films XRD (193K and 298K, respectively), room temperature lattice parameters were determined from XRD measurement of pure powders at room temperature using Rietveld refinement.

2. Synthetic methods

2.1 Synthesis of tribenzo[*b*,*d*,*f*]thiepine 9

- **Method A**

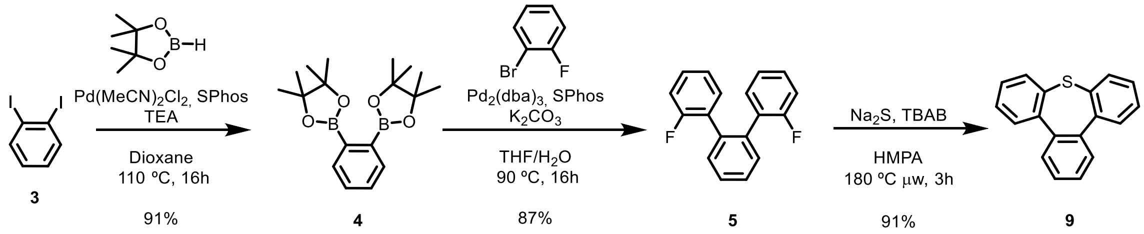

1,2-bis(4,4,5,5-tetramethyl-1,3,2-dioxaborolan-2-yl)benzene (4): Compound **4** was synthesised adapting a previous method reported for the borylation of *o*-dibromobenzene.[5]
To a Schlenk tube were added $PdCl_2(MeCN)_2$ (29 mg, 0.1 mmol) and SPhos (160 mg, 0.4 mmol). The Schlenk tube was capped with a rubber septum and then evacuated and purged with argon. Under inert atmosphere, 1,4-dioxane (6.0 mL), 1,2-diiodobenzene (1.63 g, 0.65 mL, 4.9 mmol), triethylamine (3.0 g, 4.1 mL, 29.6 mmol), and pinacolborane (3.16 g, 3.6 mL, 24.7 mmol) were added to the Schlenk tube. The reaction mixture was heated to 110 °C and the progress of the reaction was monitored by TLC. After 16 h, the reaction vessel was allowed to cool to room temperature and the reaction mixture was filtered through a thin pad of Celite with ethyl acetate as the eluent. The organic solvents were removed

under reduced pressure to give the crude product, which was purified by column chromatography on silica gel (eluent: hexane to hexane:AcOEt 9/1) to yield the desired product as pale yellow crystals (1.5 g, 4.46 mmol, 91% yield). ^{1}H NMR (300 MHz, $CDCl_3$): δ (ppm) 7.64 (dd, *J* = 5.5, 3.3 Hz, 2H), 7.37 (dd, *J* = 5.5, 3.3 Hz, 2H), 1.37 (s, 24H). The ^{1}H-NMR signals are in agreement with those reported in the literature.[5]

2,2''-difluoro-1,1':2',1''-terphenyl (5): To a mixture of compound **4** (700 mg, 2.12 mmol), 1-bromo-2-fluorobenzene (1.11 g, 0.7 mL, 6.36 mmol), $Pd_2(dba)_3$ (97 mg, 0.11 mmol) and SPhos (87 mg, 0.21 mmol) in a Schlenk tube, THF (15 mL) and an aqueous solution of K_2CO_3 (5 mL, 2 M) were added under inert atmosphere. The reaction was heated overnight at 90 ºC and after cooling down, extraction was performed with AcOEt. The resulting organic phase was dried over Na_2SO_4 and the solvent was removed in vacuo. The crude product was purified by column chromatography on silica gel (eluent: hexane) to give compound **5** as a crystalline white solid (490 mg, 1.84 mmol, 87% yield). ^{1}H NMR (300 MHz, $CDCl_3$): δ (ppm) 7.50 - 7.40 (m, 4H), 7.23 - 7.07 (m, 4H), 7.01 (dd, *J* = 7.4, 1.2 Hz, 2H), 6.92 (ddd, *J* = 9.8, 8.4, 1.2 Hz, 2H). The ^{1}H-NMR signals are in agreement with those reported in the literature.[6]

tribenzo[*b,d,f*]thiepine (9): Compound **9** was synthesised through the method reported by Foefanov *et al*.:[7] A glass tube for microwave reactor was charged with compound **5** (30 mg, 0.11 mmol), tetrabutylammonium bromide (73 mg, 0.23 mmol) and anhydrous Na_2S (88 mg, 1.13 mmol). Under inert atmosphere, HMPA (1.1 mL), previously dried over 3Å sieves, was added. The tube was placed in the microwave cavity and heated for 3h at 180 ºC. Thereafter, the tube was cooled to room temperature and the crude product was purified by flash-column chromatography on silica gel (eluent: hexane) to afford the desired compound **9** as a white solid (27 mg, 0.10 mmol, 91% yield). ^{1}H NMR (300 MHz, $CDCl_3$): δ (ppm) 7.63 (dd, *J* = 7.6, 1.5 Hz, 2H), 7.55 – 7.45 (m, 6H), 7.36 (td, *J* = 7.5, 1.5 Hz, 2H), 7.28 (td, *J* = 7.5, 1.6 Hz, 2H). The ^{1}H-NMR signals are in agreement with those reported in the literature.[7]

An up-scaled approach was also developed: A round-bottom Schlenk flask was charged with **5** (500 mg, 1.88 mmol), tetrabutylammonium bromide (1.22 g, 3.76 mmol) and anhydrous Na_2S (1.47 g, 18.8 mmol). Under inert atmosphere, HMPA (19 mL), previously dried over 3Å sieves, was added and the mixture was stirred at 180 ºC (classical heating), monitoring the reaction by TLC. After consumption of the starting material (~ 3h), the flask was cooled to room temperature and the solvent evaporated under vacuum-distillation (~120 ºC). The crude product was purified by flash-column chromatography on silica gel (eluent: hexane) to afford the desired compound **9** as a white solid (310 mg, 1.19 mmol, 63% yield).

- **Method B**

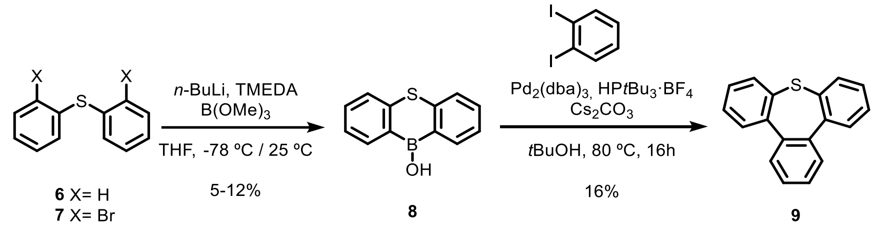

Borinic acid **8** was prepared from diphenylsulfane **6** or bis(2-bromophenyl)sulfane **7** following the procedures already reported in the literature.[8]

tribenzo[*b,d,f*]thiepine (9): Borinic acid **8** (21 mg, 0.1 mmol), $Pd_2(dba)_3$ (1.4 mg, 0.0015 mmol), *t*$Bu_3PH(BF_4)$ (0.9 mg, 0.003 mmol) and Cs_2CO_3 (107 mg, 0.33 mmol) were added to an oven-dried Schlenk tube equipped with a magnetic stirring bar. The flask was evacuated and refilled with argon three times, then *t*BuOH (2 mL) and 1,2-diiodobenzene (66 mg, 30 µL, 0.2 mmol) were added. The reaction was allowed to stir for 1 h at room temperature prior to heating to 80 ºC for 16 h. The reaction

medium was cooled to room temperature, diluted with EtOAc, filtered over Celite, and concentrated in vacuo. The crude product was purified by column chromatography on silica gel (eluent: hexane) to yield the desired product **9** as a white solid (4 mg, 0.016 mmol, 16% yield).

2.2 General procedure for the oxidation of tribenzothiepine 9 with *m*CPBA

A solution of tribenzothiepine **9** (20 mg, 0.077 mmol) in CH_2Cl_2 (1 mL) was cooled to the desired temperature and the additive (0.616 mmol, 8.0 equiv.) was added under inert atmosphere. A solution of *m*CPBA (18 mg, 0.081 mmol, 1.05 equiv.) in CH_2Cl_2 (1 mL) was added dropwise to the resulting mixture. The reaction was stirred at the same temperature for 1 h, following the progress by TLC. The mixture was quenched by the addition of NaOH (1 mL, 1 M aqueous solution), the aqueous phase extracted with CH_2Cl_2 and the resulting organic phase dried over Na_2SO_4 before removing the solvent in vacuo. The crude product was purified by column chromatography on silica gel (eluent: CH_2Cl_2) isolating four different fractions. The first fraction corresponds to starting material **9**, the second fraction was characterised as the tribenzothiepine *S*,*S*-dioxide **10**, the third fraction corresponds to the *exo* isomer of the tribenzothiepine *S*-oxide *exo*-**1**, with the last and fourth fraction being the *endo* isomer *endo*-**1**.

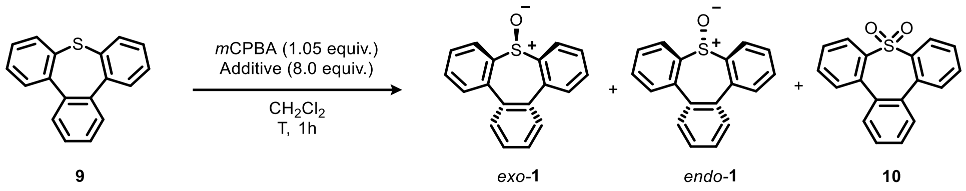

Entry	Additive	T (°C)	yield (%)[a]	*exo*-**1**:*endo*-**1**:**10**
1	/	-80	52	14:0:86
2	$BF_3.OEt_2$	-80	94	66:33:1
3	$BF_3.OEt_2$	-10	96	70:29:1
4	AgTFA	-10	64	56:35:9
5	HFIP[b]	-10	98	43:48:9
6	BCF[c]	-10	95	0:100:0

[a] Combined yield of *exo*-**1**, *endo*-**1** and **10**. [b] 1,1,1,3,3,3-hexafluoroisopropanol.
[c] tris(2,3,4,5,6-pentafluorophenyl)borane

***exo*-tribenzo[*b,d,f*]thiepine *S*-oxide (*exo*-1):** ^{1}H NMR (500 MHz, $CDCl_3$): δ (ppm) 7.90 (d, *J* = 7.8 Hz, 2H, H_1), 7.62 – 7.53 (m, 6H, $H_5/H_6/H_2$), 7.48 – 7.43 (m, 4H, H_4/H_3). ^{13}C NMR (125 MHz, $CDCl_3$): δ (ppm) 146.6 (C_6), 136.4 (C_7), 134.2 (C_5), 130.3 (C_8), 130.3 (C_4), 130.0 (C_3), 128.9 (C_9), 128.7 (C_2), 120.0 (C_1). HRMS (TOF-MS ES+): m/z calcd for $C_{18}H_{13}SO$: 277.0687, found: 277.0686 [$M+H^+$].

***endo*-tribenzo[*b,d,f*]thiepine *S*-oxide (*endo*-1):** ^{1}H NMR (500 MHz, $CDCl_3$): δ (ppm) 7.72 (dd, *J* = 7.7, 1.4 Hz, 2H, H_1), 7.70 – 7.67 (m, 2H, H_5), 7.64 (dd, *J* = 7.8, 1.3 Hz, 2H, H_4), 7.58 – 7.55 (m, 2H, H_6), 7.54 (td, *J* = 7.6, 1.4 Hz, 2H, H_3), 7.42 (td, *J* = 7.6, 1.3 Hz, 2H, H_2). ^{13}C NMR (125 MHz, $CDCl_3$): δ (ppm) 144.9 (C_6), 137.1 (C_5), 136.4 (C_7), 132.6 (C_4), 131.8 (C_3), 130.0 (C_8), 128.6 (C_9), 128.0 (C_2), 126.5 (C_1). HRMS (TOF-MS ES+): m/z calcd for $C_{18}H_{13}SO$: 277.0687, found: 277.0689 [$M+H^+$].

tribenzo[*b,d,f*]thiepine *S,S*-dioxide (10): ^{1}H NMR (300 MHz, $CDCl_3$): δ (ppm) 8.16 (d, *J* = 7.8 Hz, 2H), 7.70 – 7.63 (m, 6H), 7.61 – 7.51 (m, 4H). HRMS (TOF-MS ES+): m/z calcd for $C_{18}H_{13}SO_2$: 293.0636, found: 293.0639 [$M+H^+$]. Data in agreement with those reported in the literature.[9]

3. Mass spectrometry

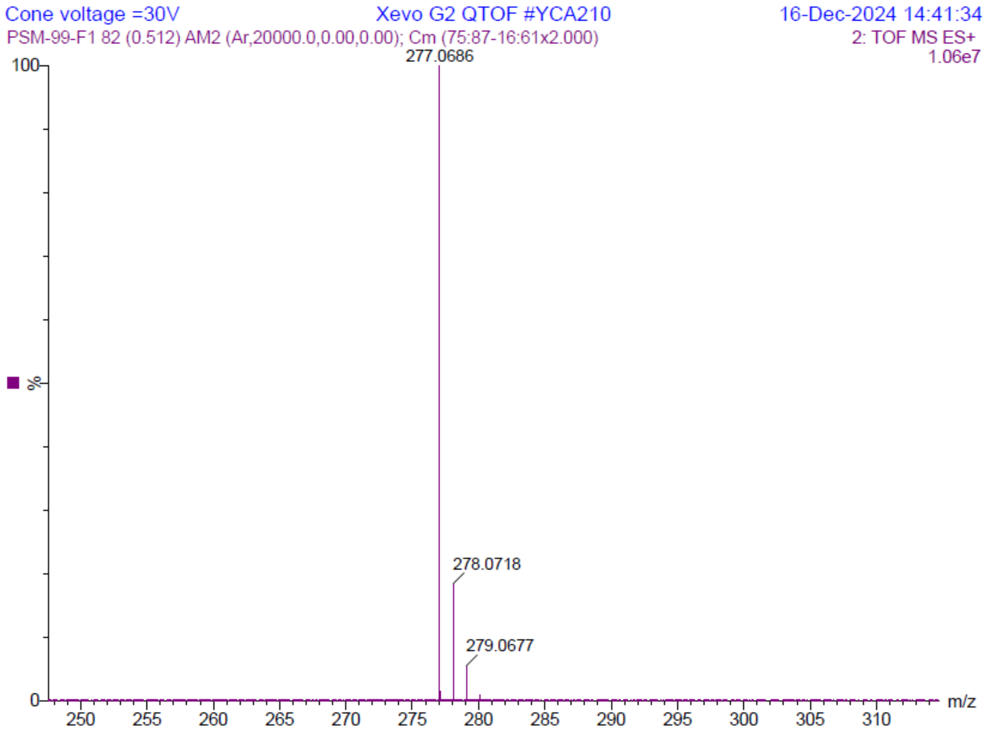

*Figure S1. High Resolution Mass Spectrometry spectrum of sulfoxide exo-**1** (TOF-MS ES+).*

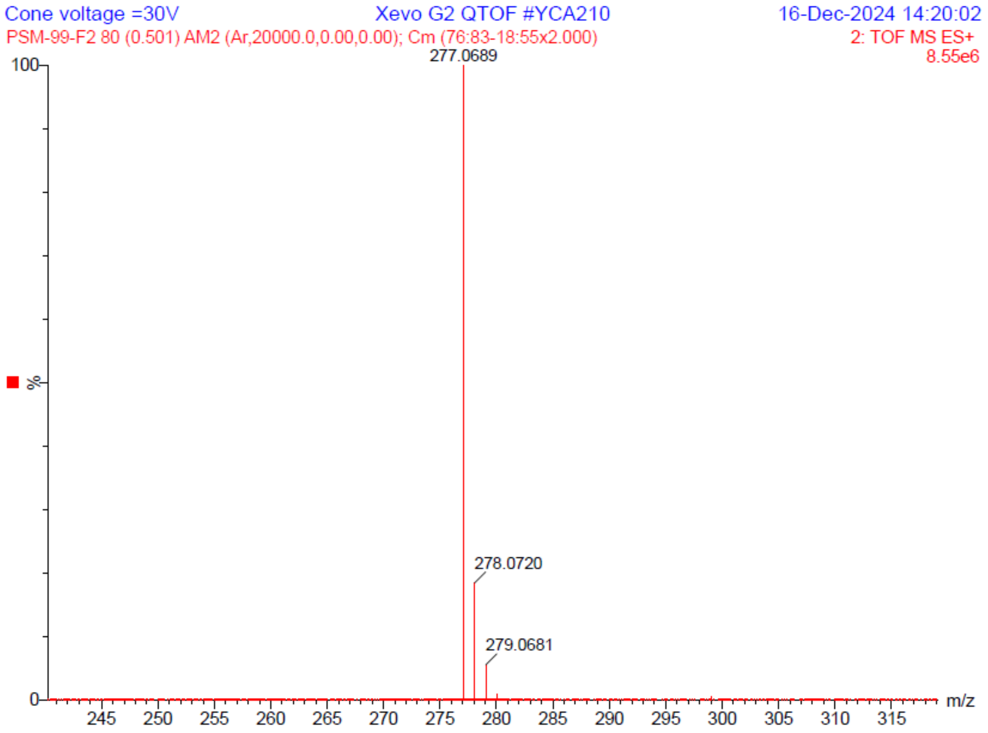

*Figure S2. High Resolution Mass Spectrometry spectrum of sulfoxide endo-**1** (TOF-MS ES+).*

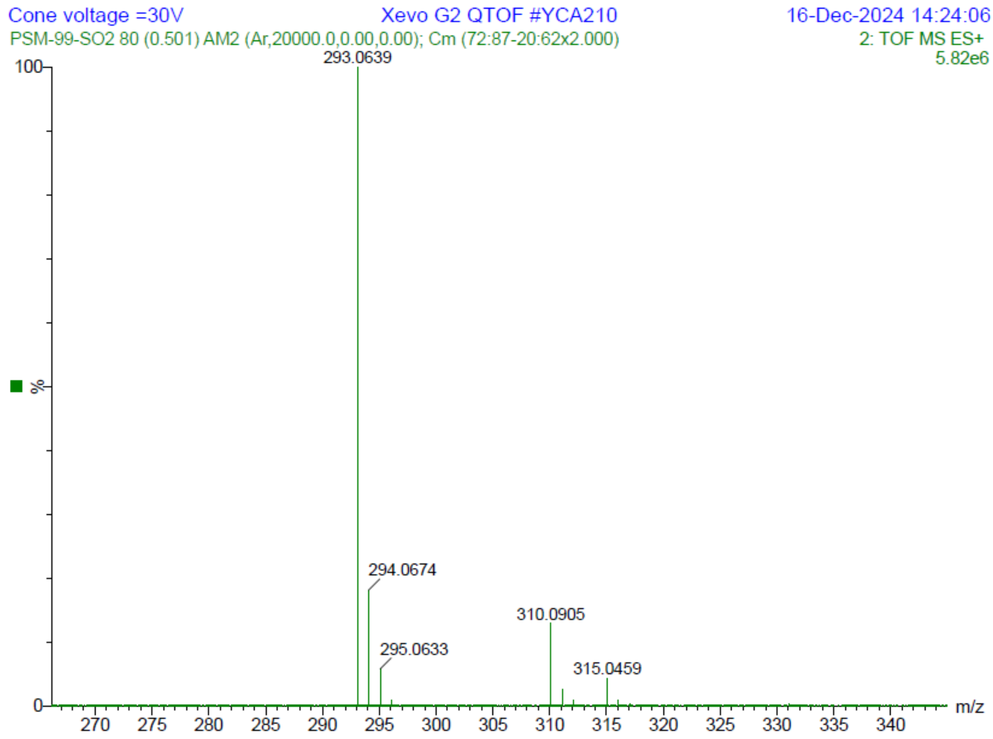

*Figure S3. High Resolution Mass Spectrometry spectrum of sulfone **10** (TOF-MS ES+).*

4. NMR spectroscopy

4.1 Characterisation of synthetic intermediates, thiepine 9 and thiepine *S,S*-dioxide 10

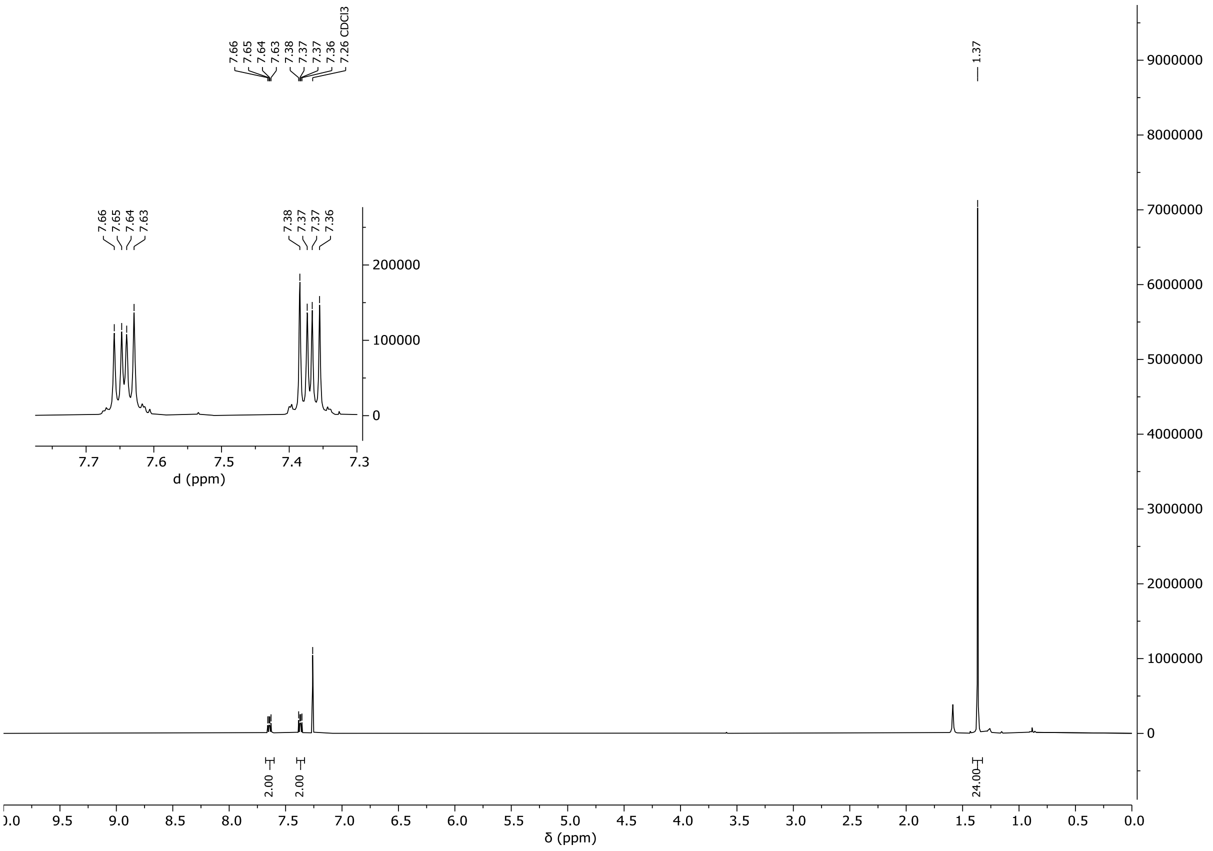

*Figure S4. ^{1}H-NMR spectrum of **4**, in $CDCl_3$ (300 MHz).*

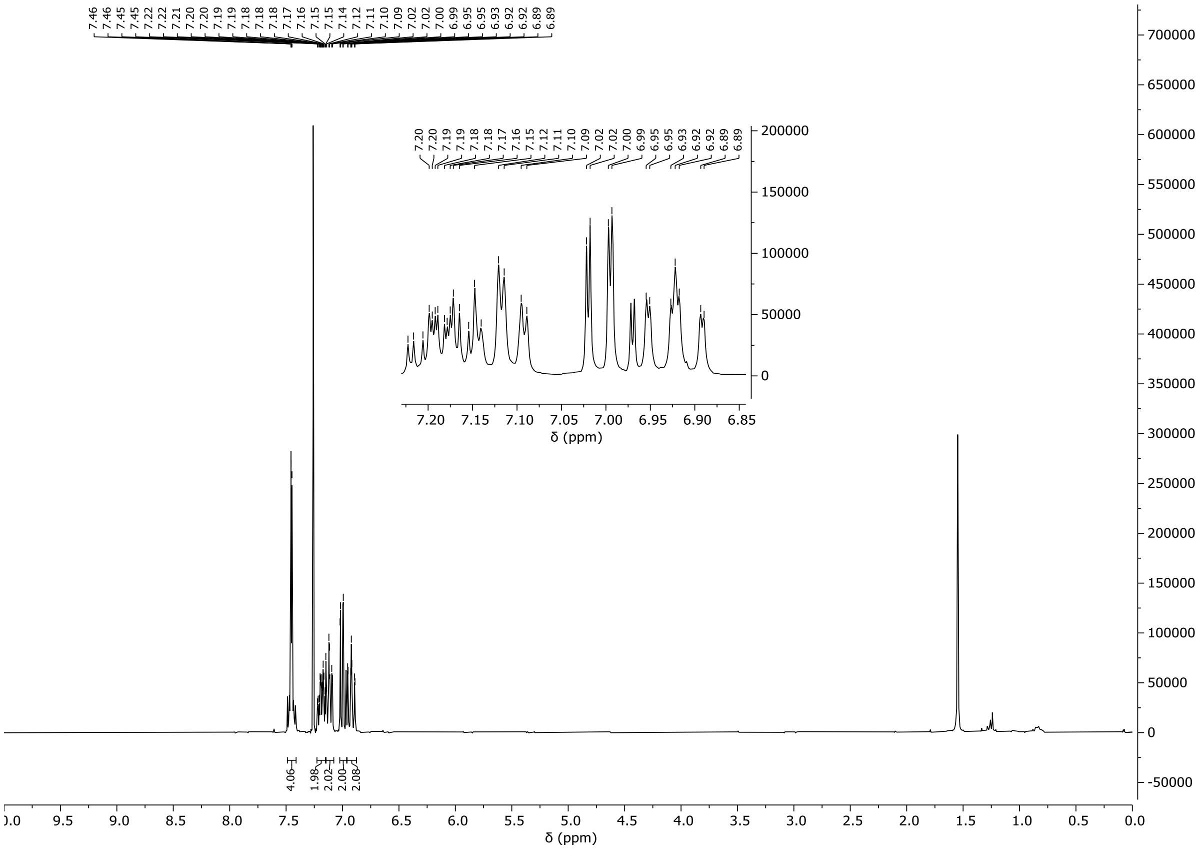

Figure S5. *^{1}H-NMR spectrum of* ***5****, in $CDCl_3$ (300 MHz).*

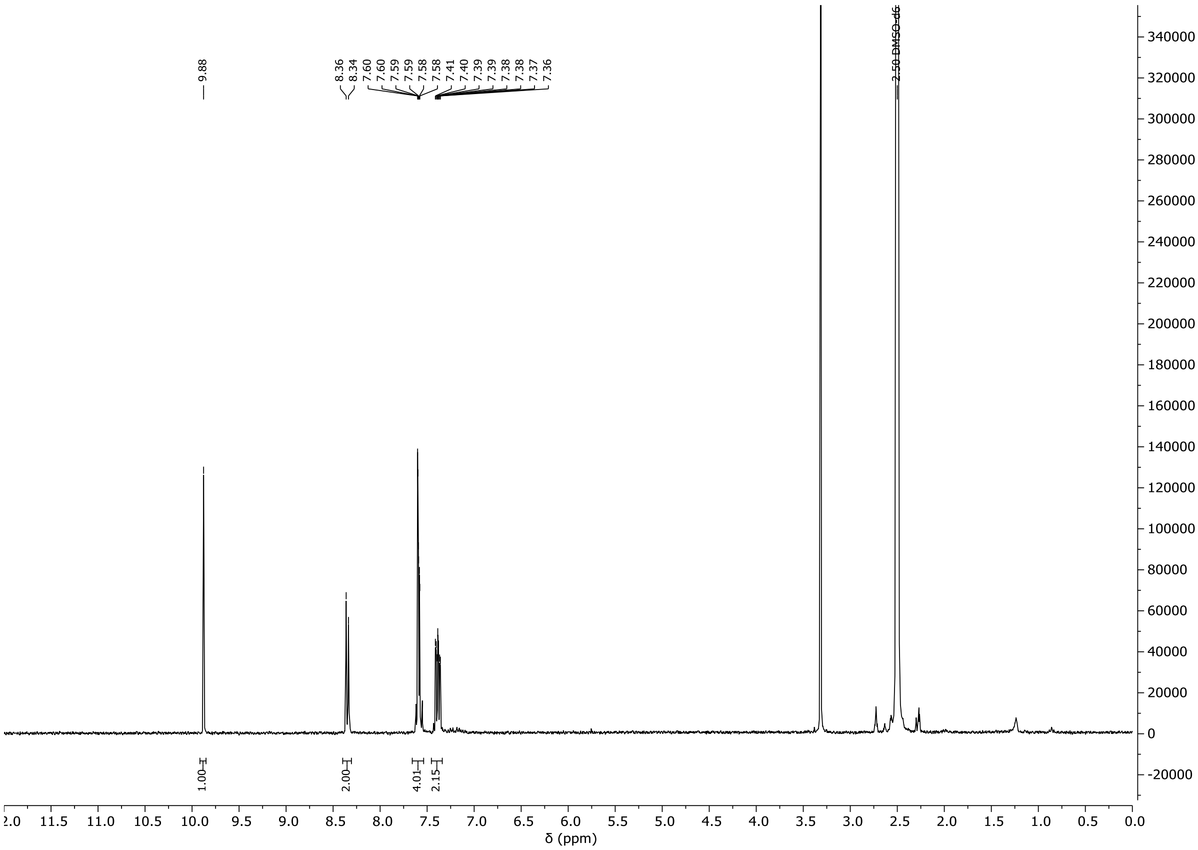

Figure S6. *^{1}H-NMR spectrum of* ***8****, in DMSO-d6 (300 MHz).*

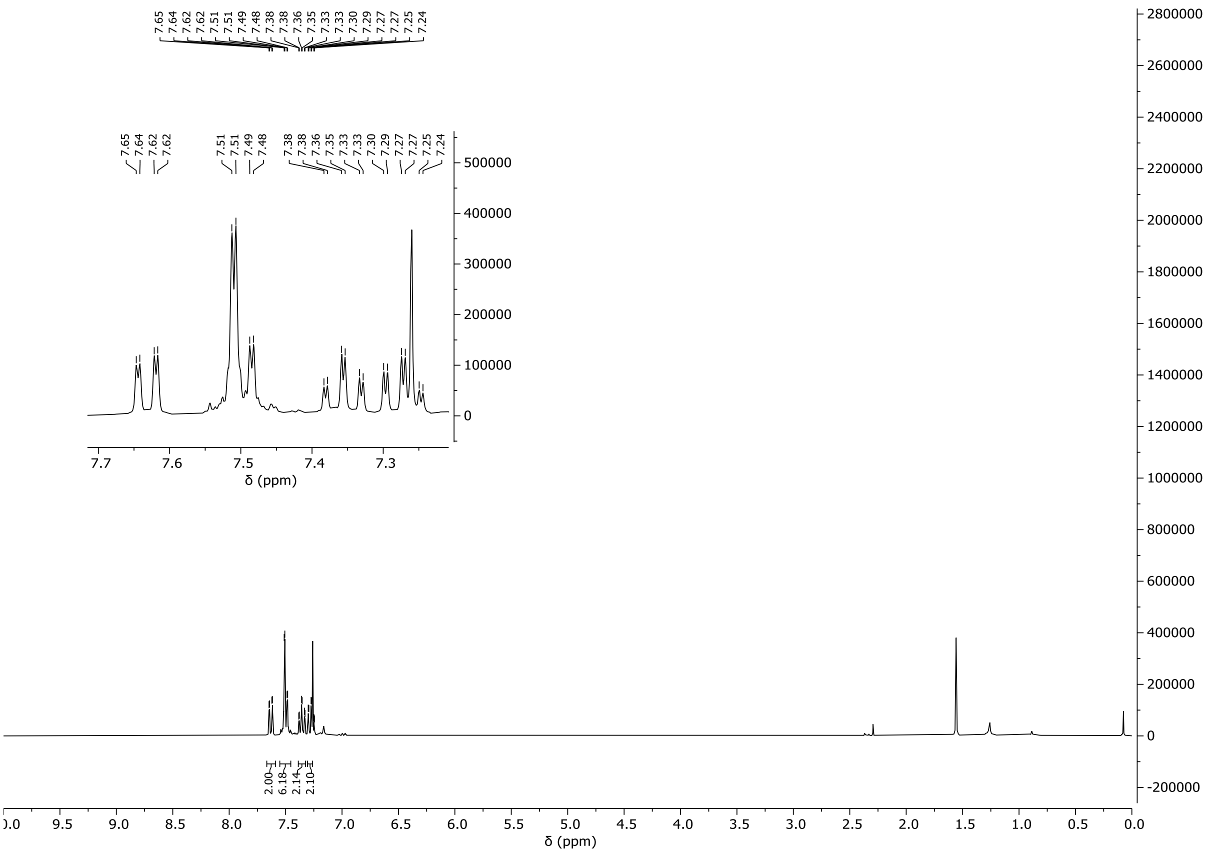

*Figure S7. ^{1}H-NMR spectrum of **9**, in $CDCl_3$ (300 MHz).*

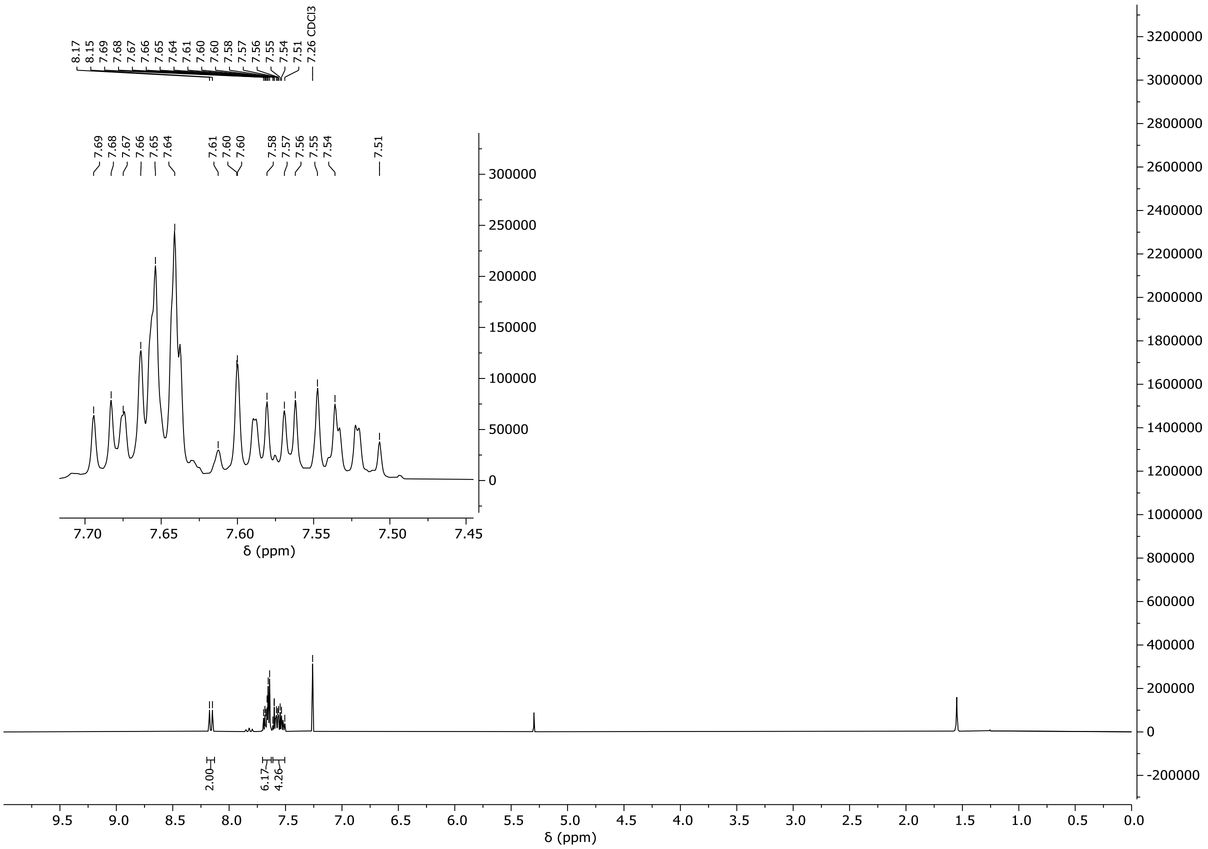

*Figure S8. ^{1}H-NMR spectrum of **10**, in $CDCl_3$ (300 MHz).*

4.2 Characterisation of thiepine *S*-oxide *exo*-1

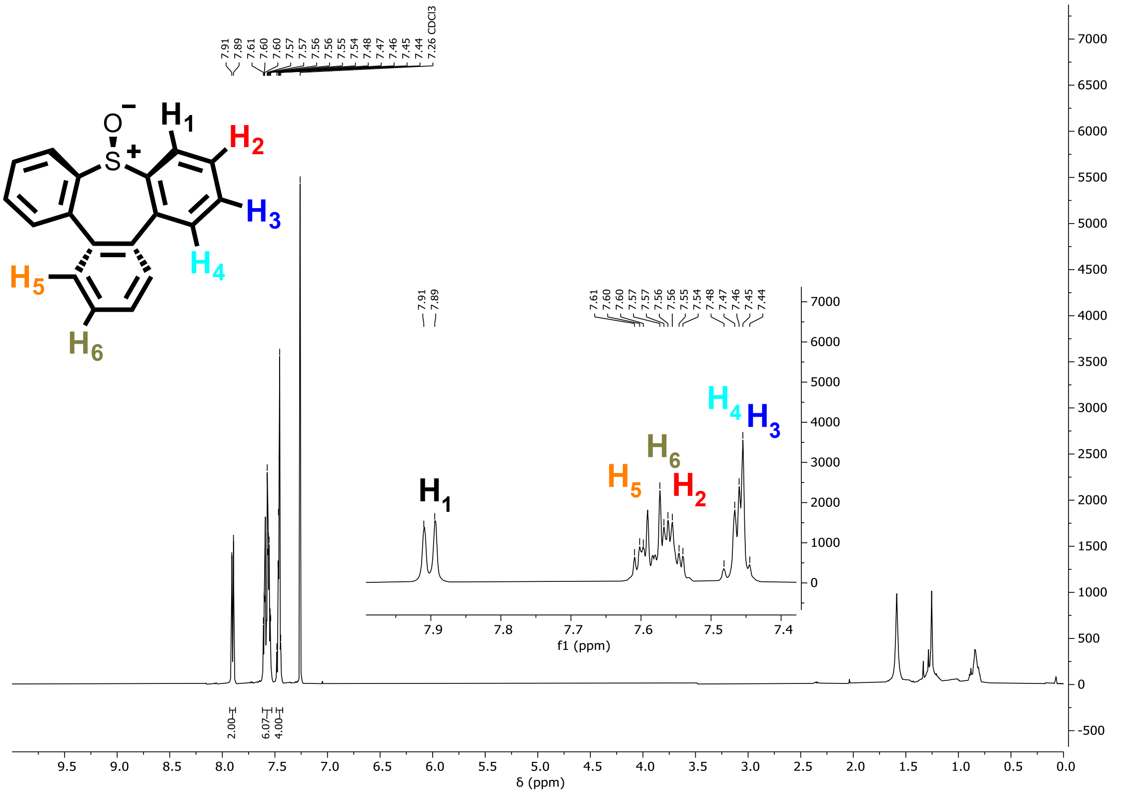

*Figure S9. ^{1}H-NMR spectrum of exo-**1**, in $CDCl_3$ (500 MHz).*

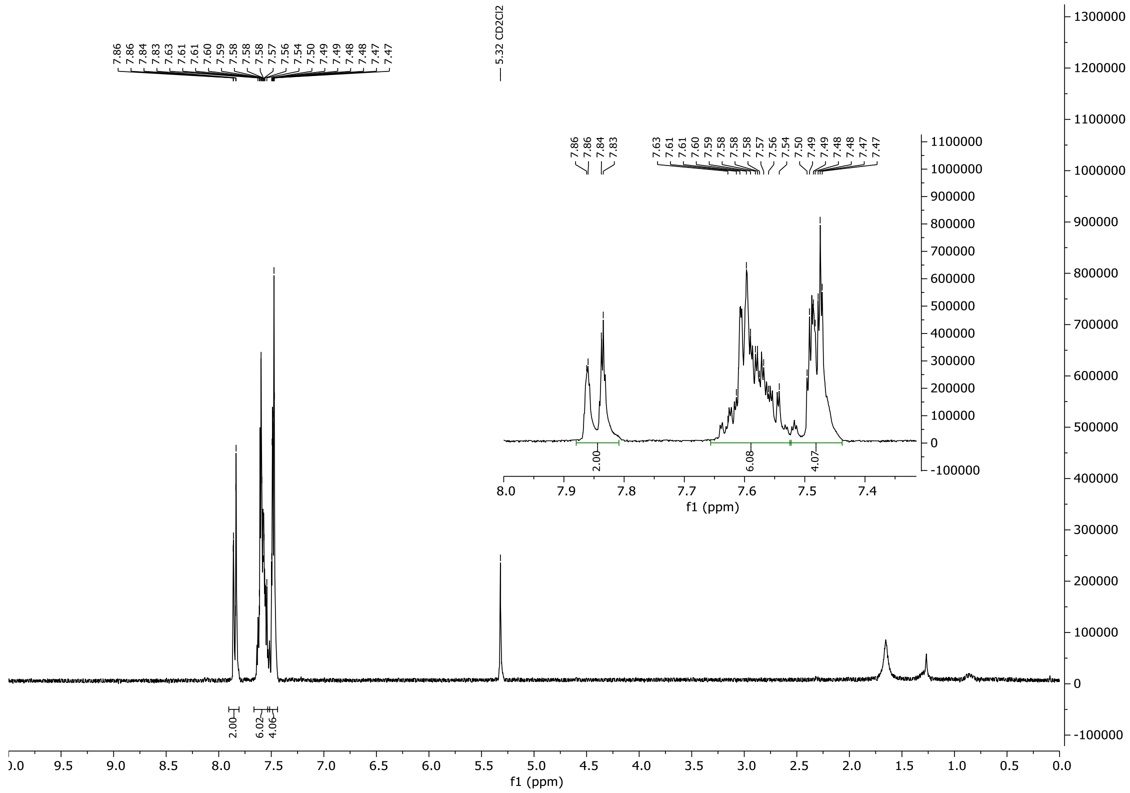

*Figure S10. ^{1}H-NMR spectrum of exo-**1**, in CD_2Cl_2 (300 MHz).*

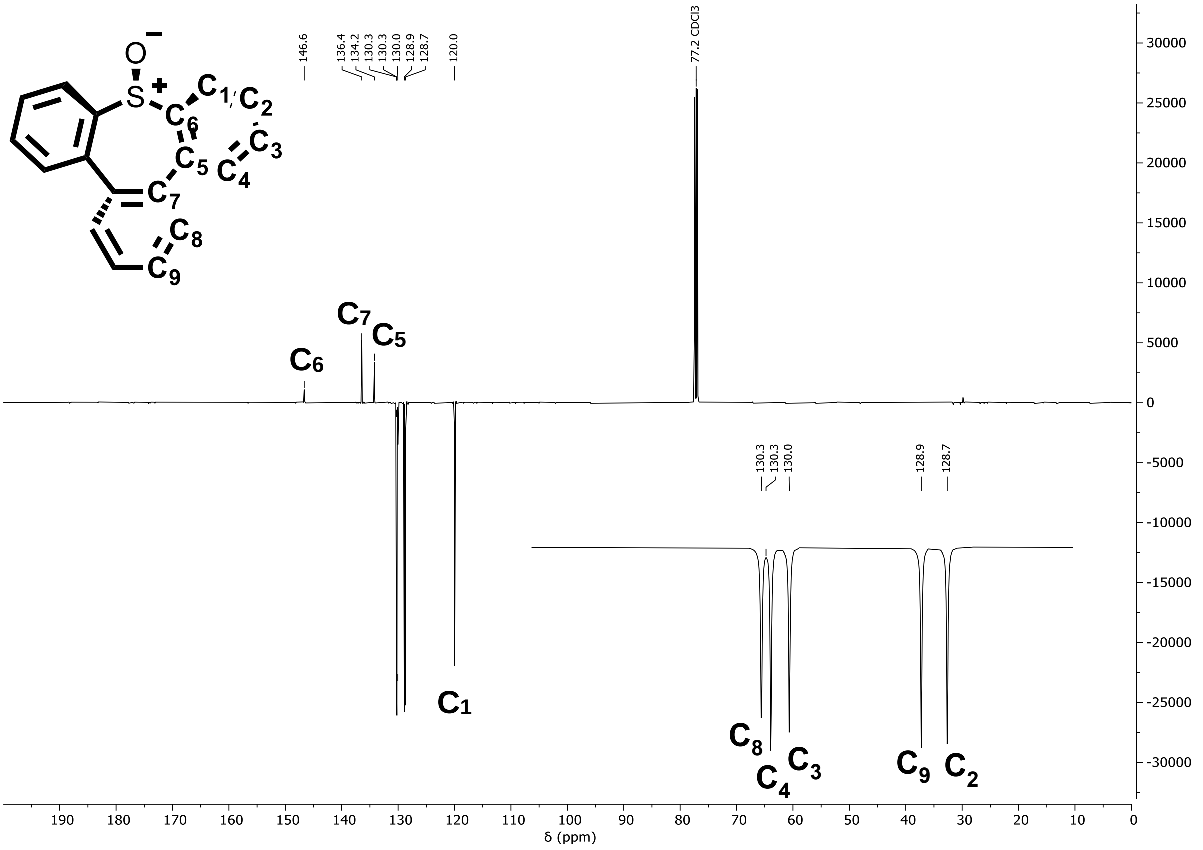

Figure S11. [13]*C-Jmod-NMR spectrum of exo-**1**, in CDCl₃ (125 MHz).*

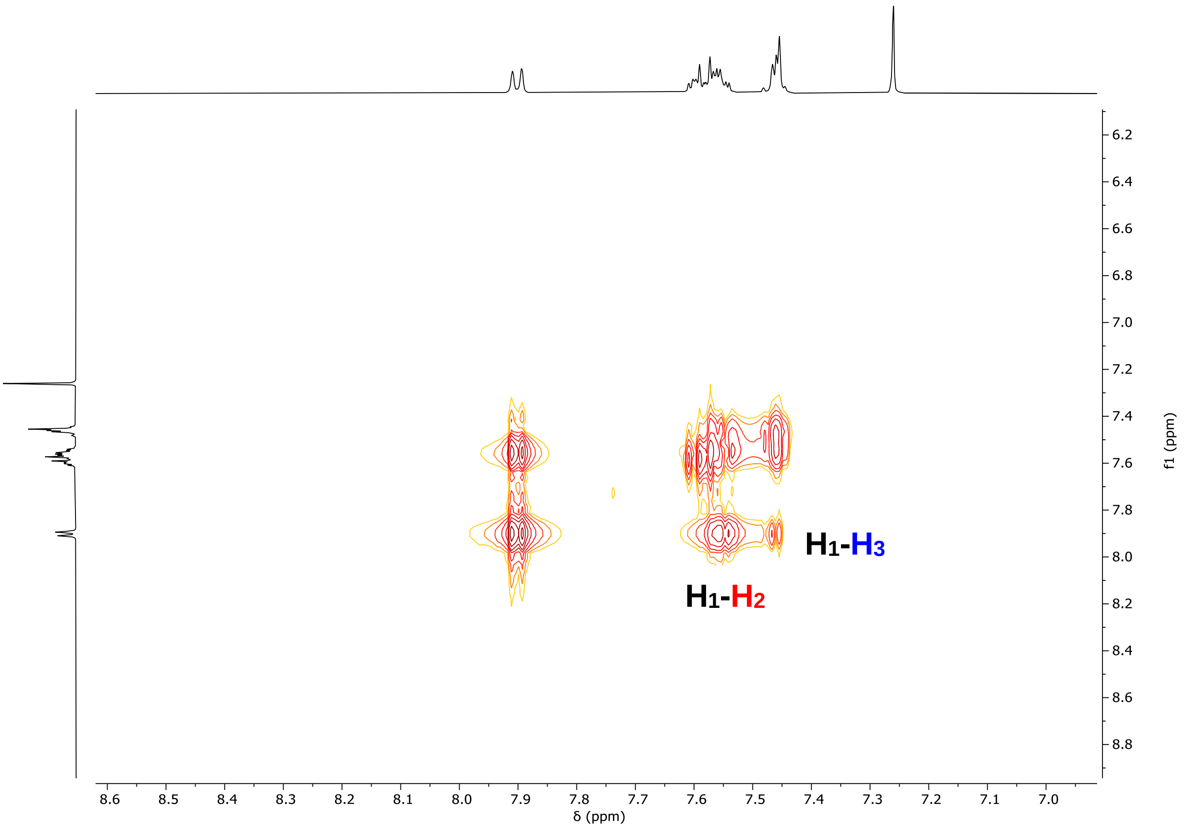

*Figure S12. COSY spectrum of exo-**1**, in CDCl₃.*

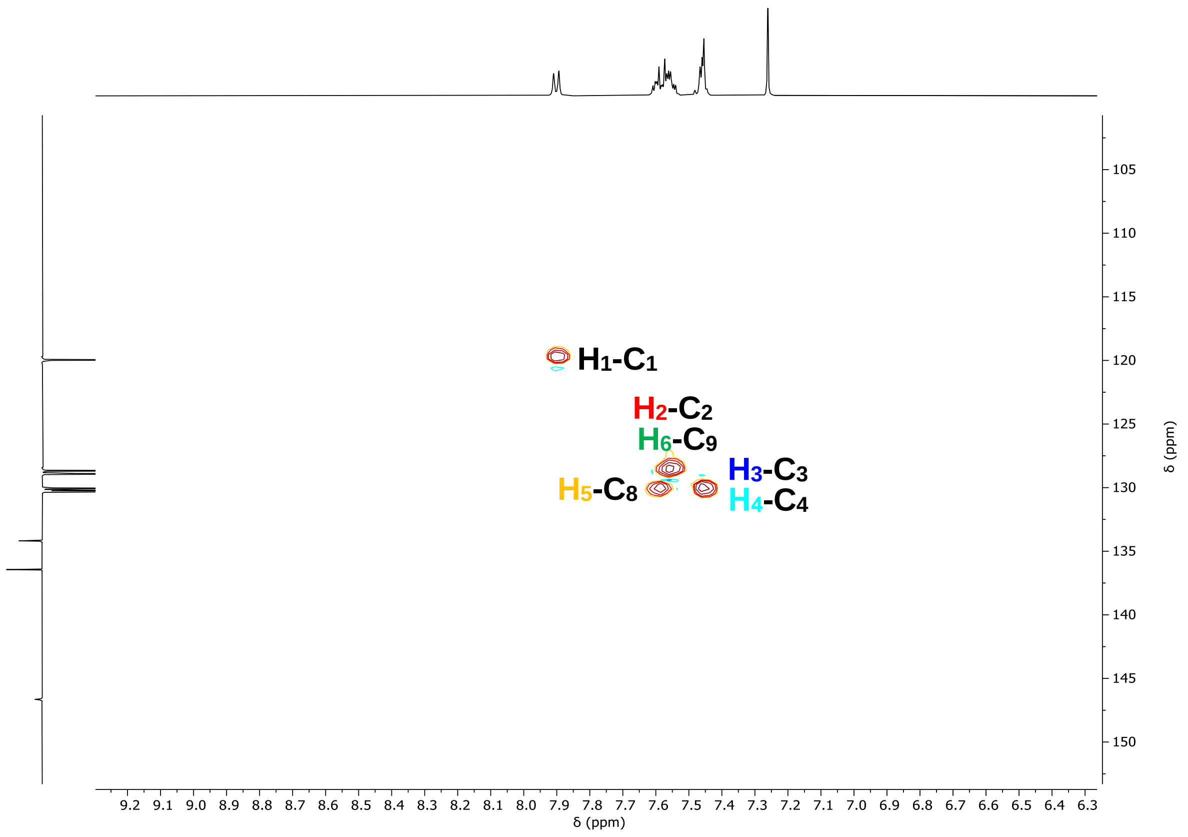

*Figure S13. HSQC spectrum of exo-**1**, in $CDCl_3$.*

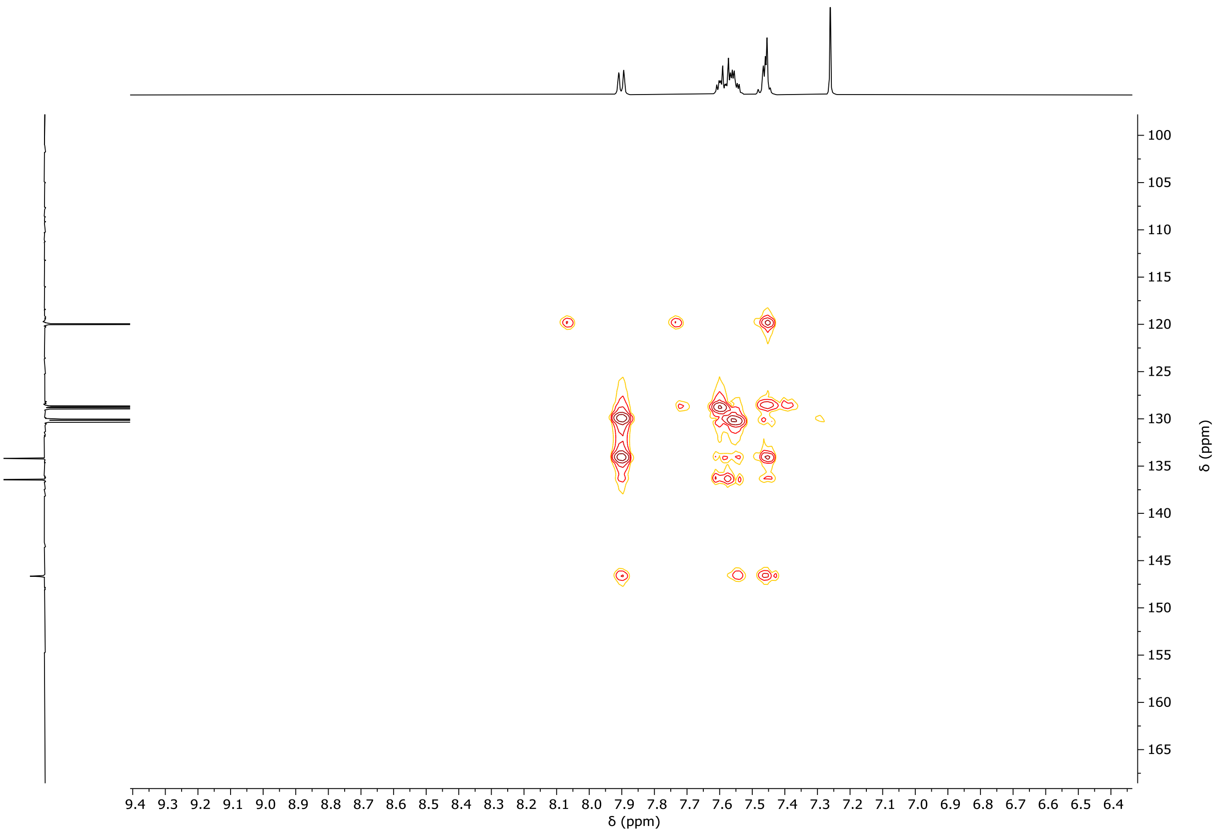

*Figure S14. HMBC spectrum of exo-**1**, in $CDCl_3$.*

4.3 Characterisation of thiepine *S*-oxide *endo*-1

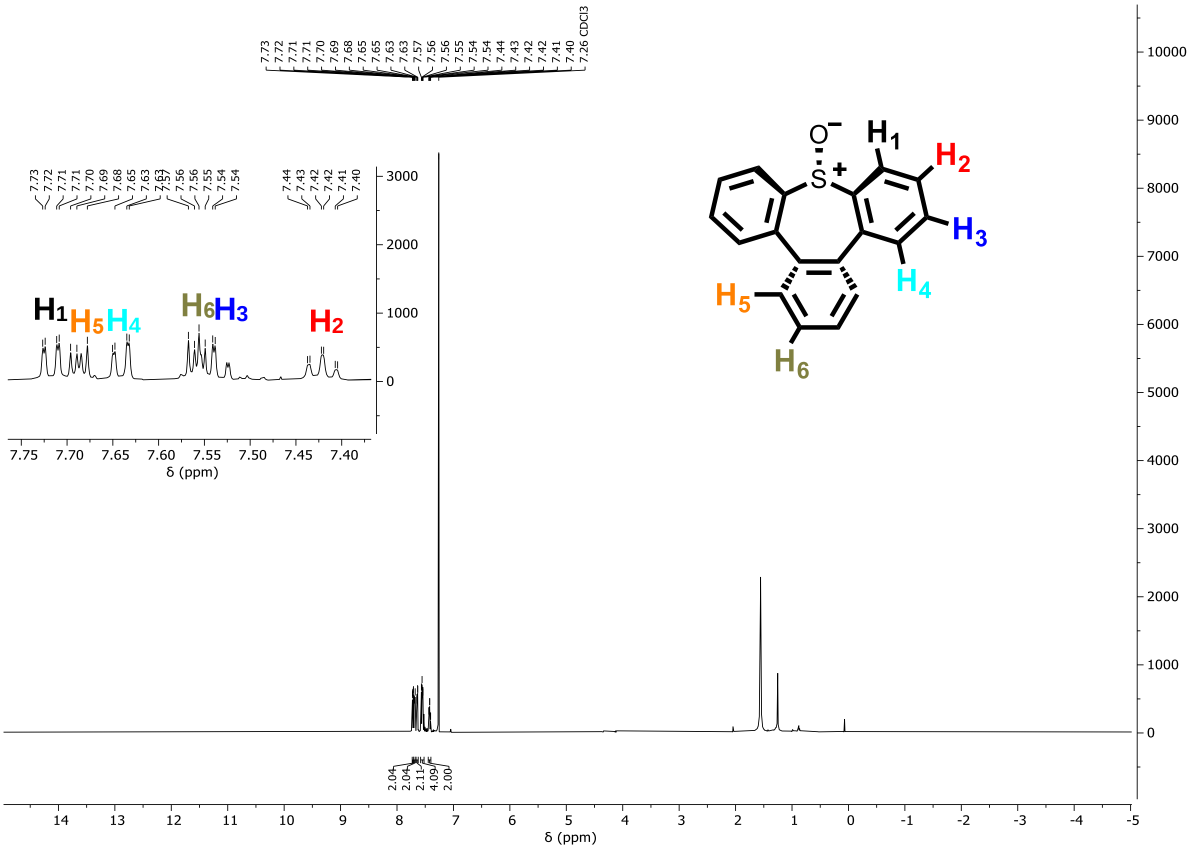

*Figure S15. ^{1}H-NMR spectrum of endo-**1**, in $CDCl_3$ (500 MHz).*

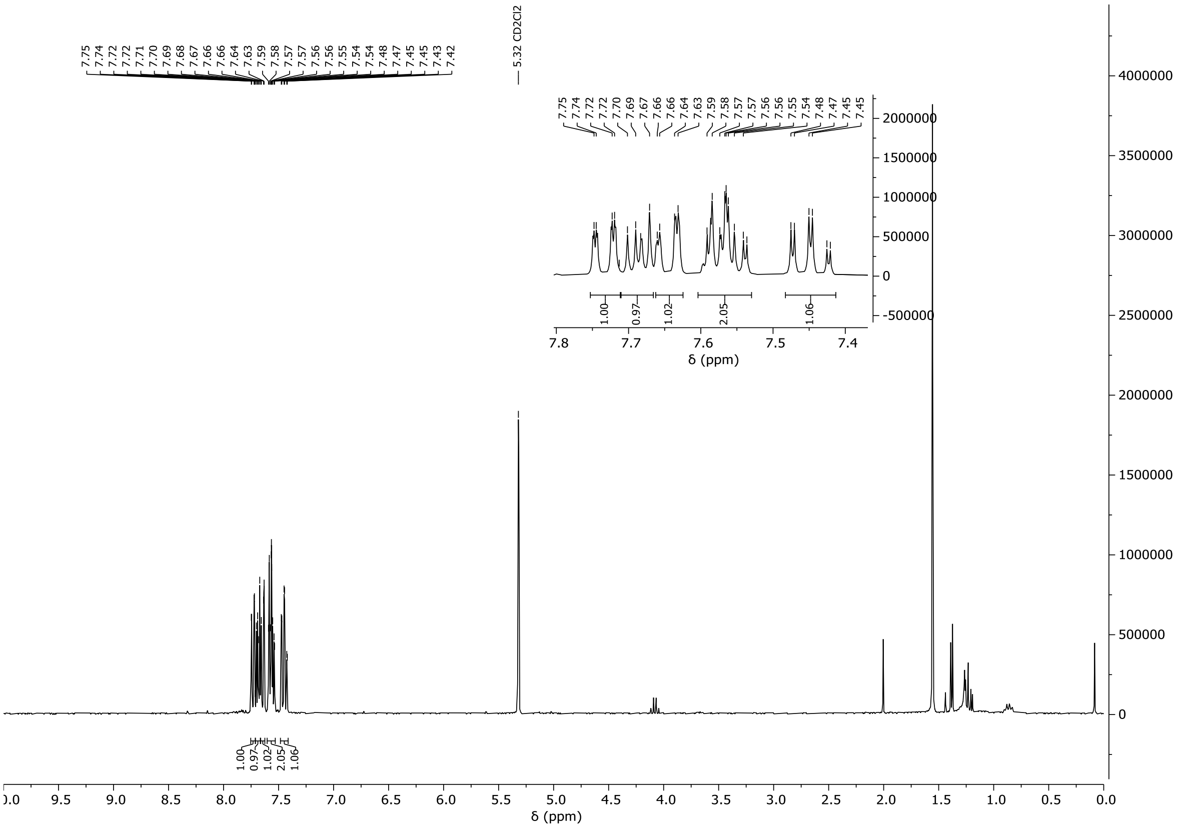

*Figure S16. ^{1}H-NMR spectrum of endo-**1**, in CD_2Cl_2 (300 MHz).*

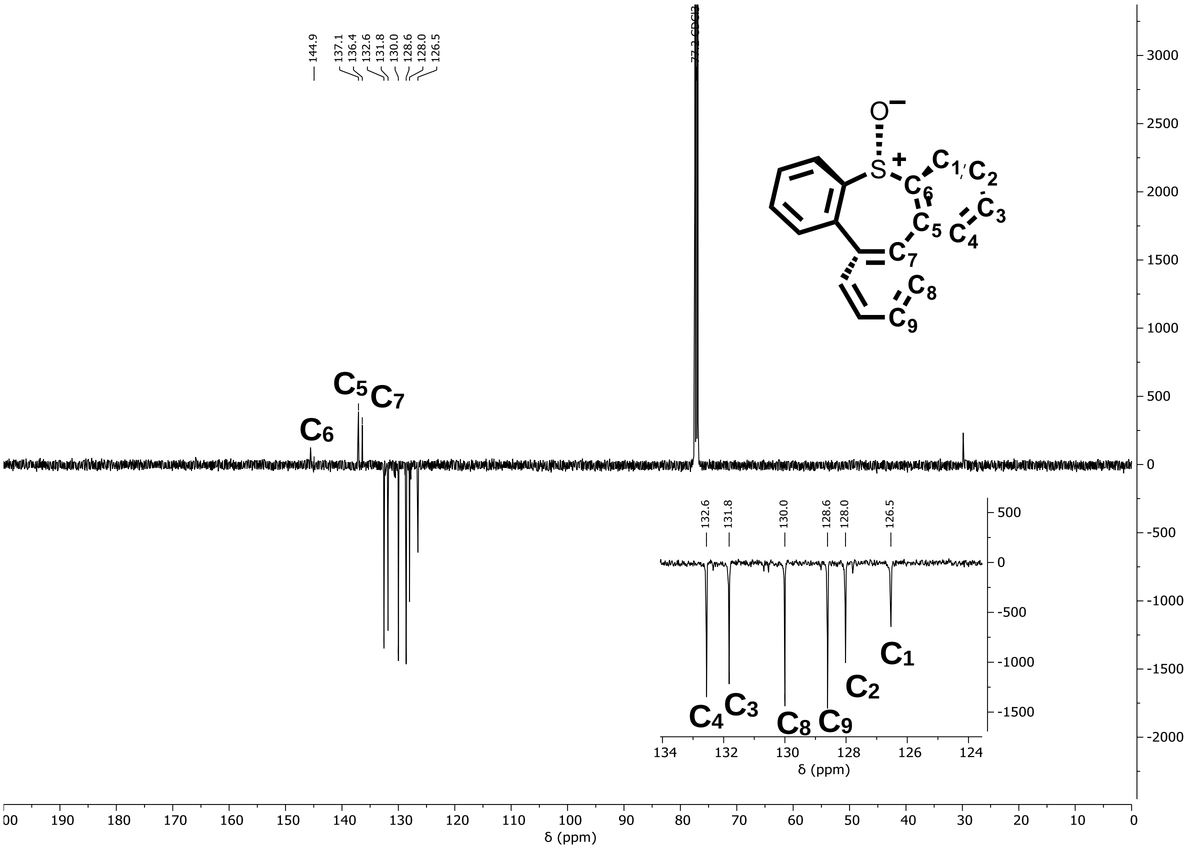

Figure S17. ^{13}*C-Jmod-NMR spectrum of endo-***1***, in* $CDCl_3$ *(125 MHz).*

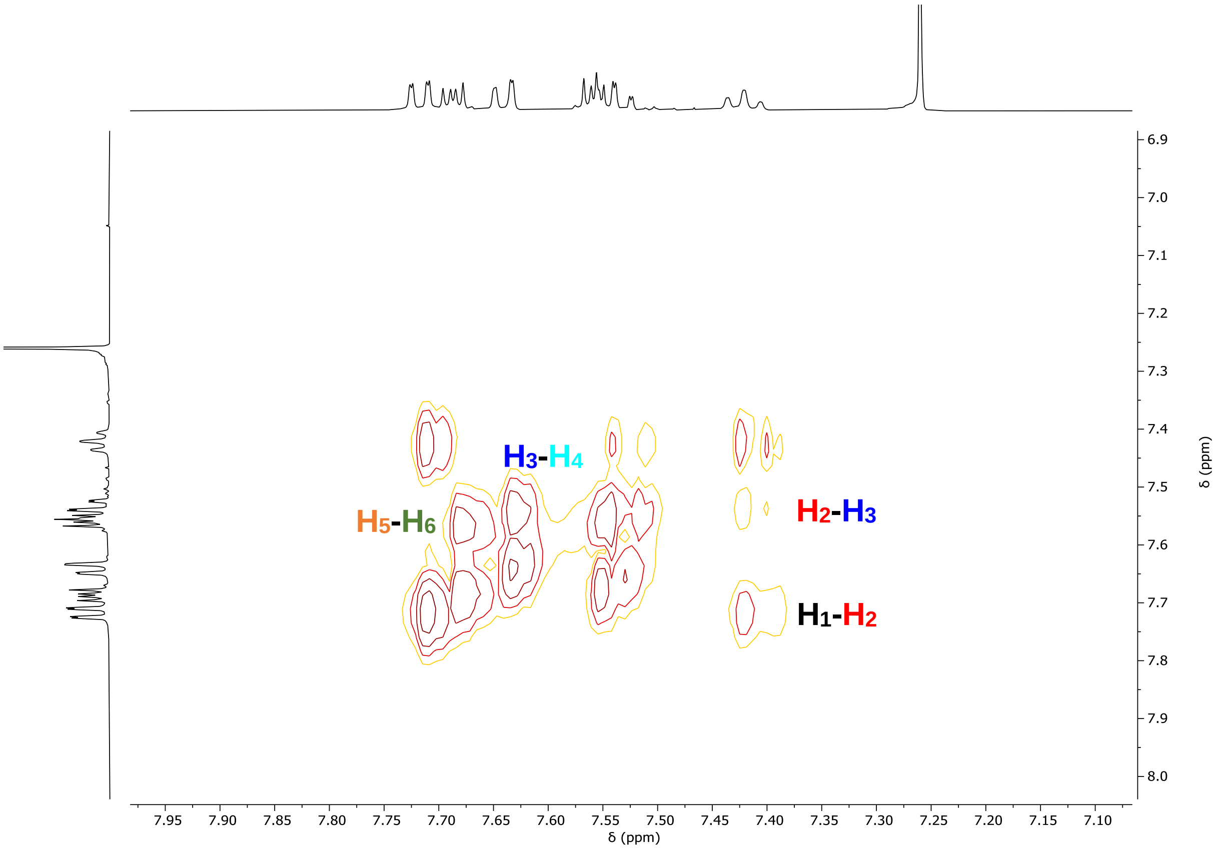

*Figure S18. COSY spectrum of endo-***1***, in* $CDCl_3$*.*

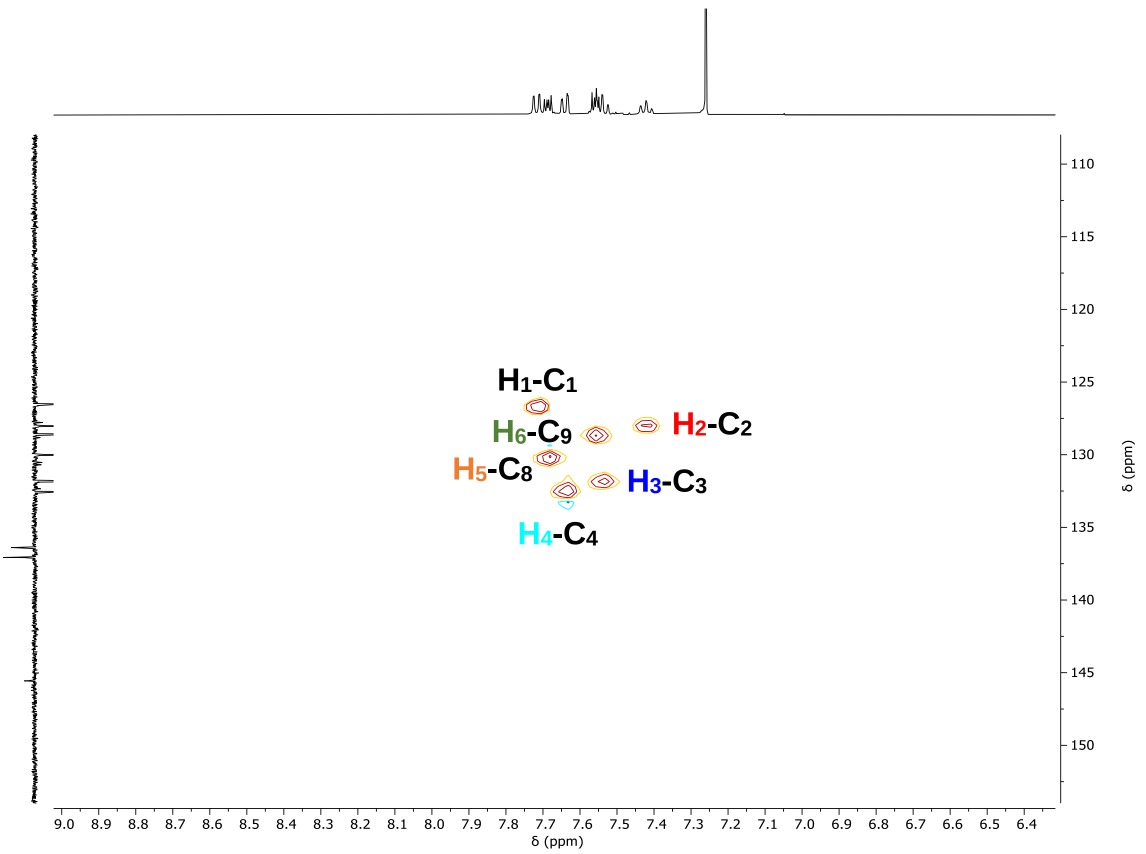

*Figure S19. HSQC spectrum of endo-**1**, in $CDCl_3$.*

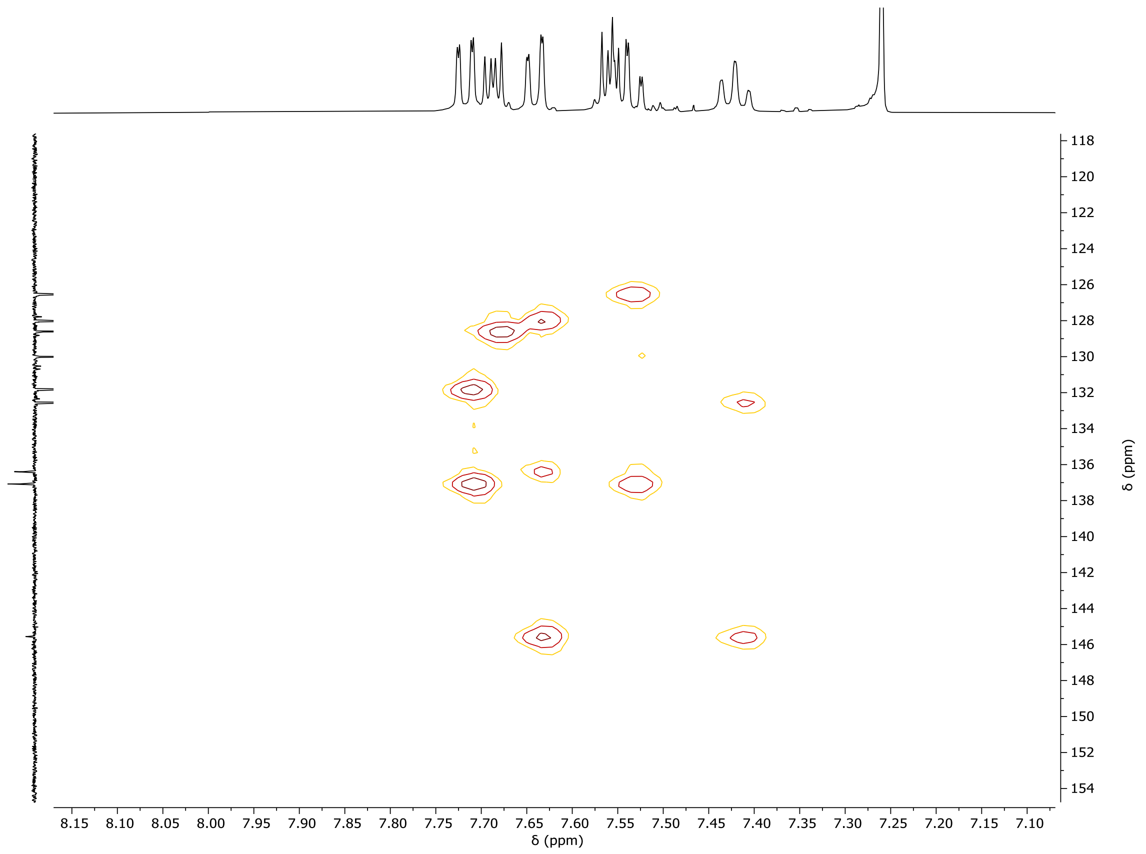

*Figure S20. HMBC spectrum of endo-**1**, in $CDCl_3$.*

5. Electrochemical characterisation

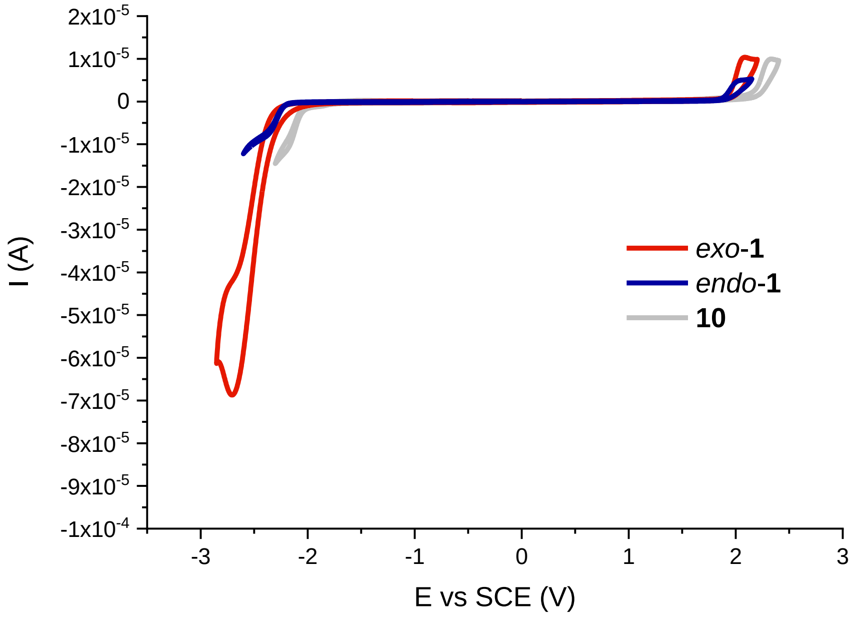

*Figure S21. Cyclic voltammograms of exo-**1** (red curve), endo-**1** (blue curve) and **10** (grey curve), ca. 1 mM in CH_2Cl_2 (0.10 M Bu_4NPF_6, scan rate 100 mV.s^{-1}, Pt working electrode).*

6. Reactivity studies

6.1 Investigations by NMR spectroscopy

Thermal reactivity

Variable temperature (VT)-^{1}H NMR was used to study the thermal isomerisation of *endo*-**1** into *exo*-**1** (Fig. S22 and S23) and assess the thermal stability of *exo*-**1** (Fig. S24). For a better understanding of the time-dependent experiments, the signals ascribed to the protons located *ortho* to the sulfoxide moiety are indicated with a blue (*endo*-**1**) and a red (*exo*-**1**) dot, respectively.

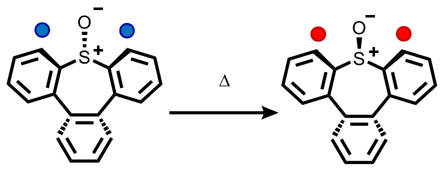

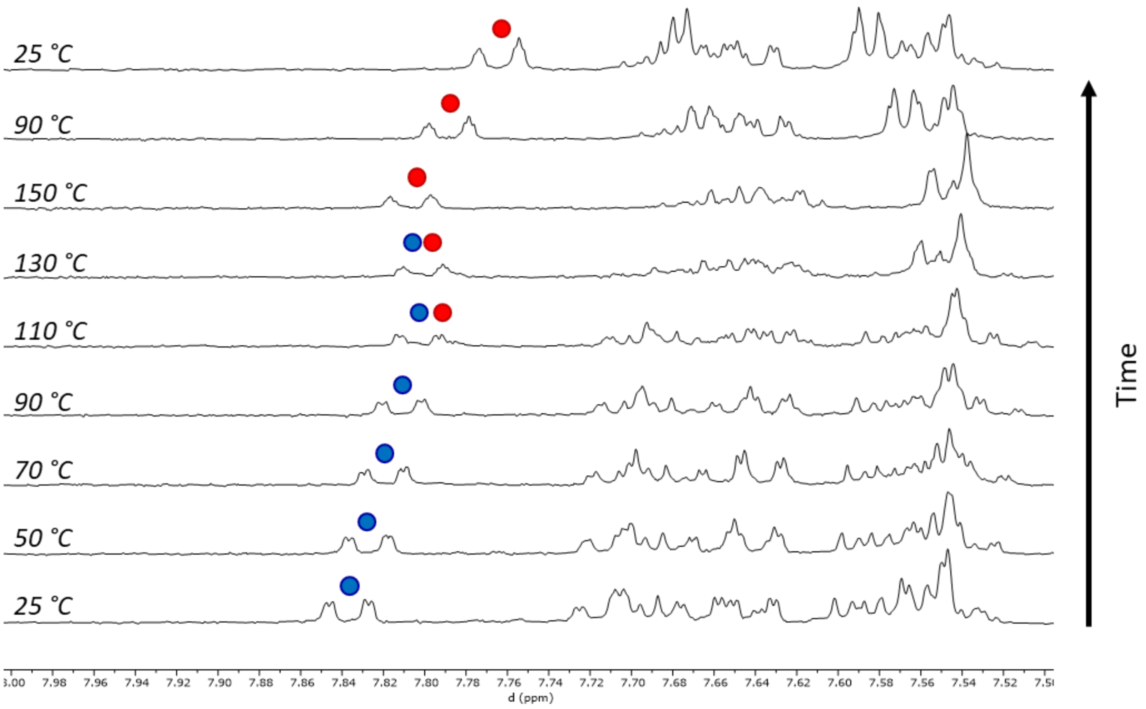

*Figure S22. Variable temperature ^{1}H-NMR spectra (500 MHz) of endo-**1** in DMSO-d6, showing its thermal isomerisation into exo-**1**. The NMR sample was progressively heated and measured at temperatures ranging from 25 °C to 150 °C, and then cooled down again to room temperature. At 110 °C the isomerisation of endo-**1** (blue dots) into exo-**1** can be already detected in the coalescence signal at 7.81 ppm. The reaction proceeds until 150 °C and eventually only the thermodynamic product exo-**1** (red dots) is recovered. We confirmed by mass spectrometry the partial deoxygenation of the sulfoxide under these conditions, thus releasing some tribenzothiepine **9**.*

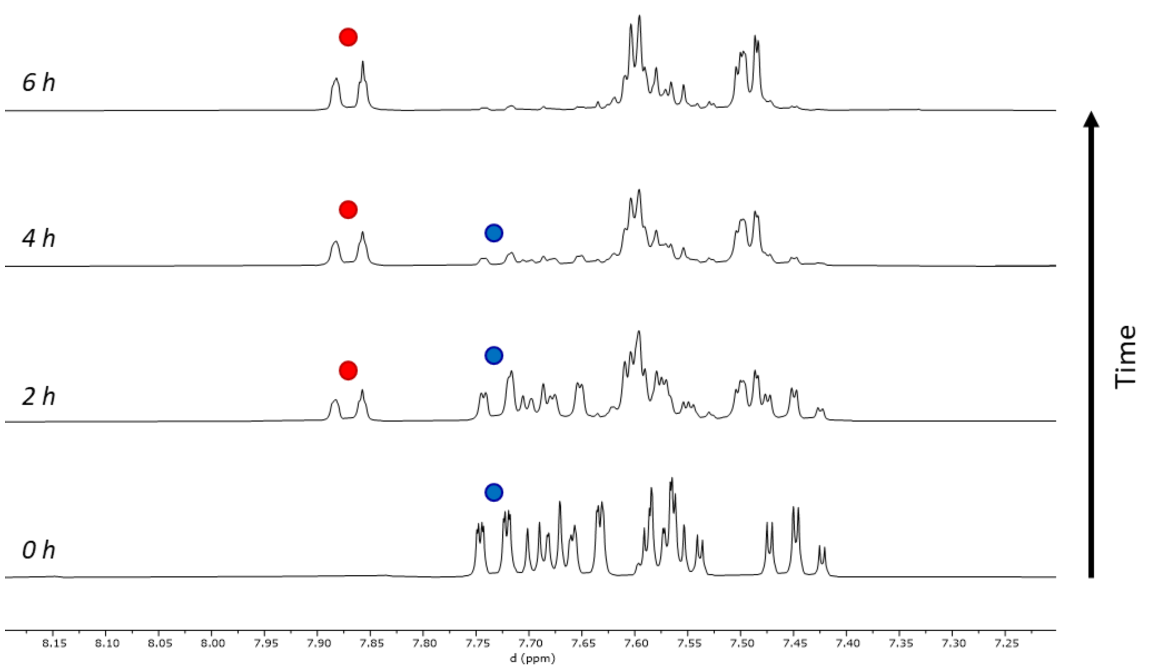

*Figure S23. Room-temperature ^{1}H-NMR spectra (300 MHz) of a sample of endo-**1** in tetrachloroethane-d2, recorded at t_0 (bottom) and after heating at 110 ºC for 2h, 4h and 6h, respectively. The endo-isomer of **1** (blue dots) is quantitatively transformed into exo-**1** (red dots) after 6h at 110 ºC.*

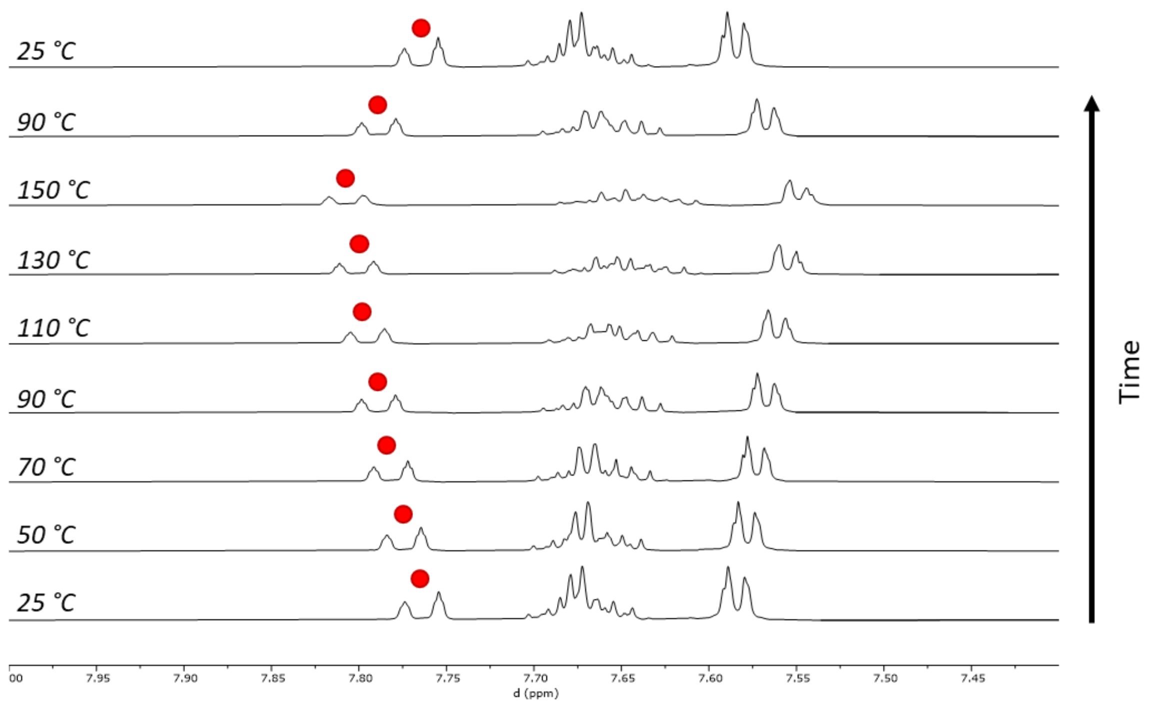

*Figure S24. Variable temperature ^{1}H-NMR spectra (500 MHz) of exo-**1** in DMSO-d6. The NMR sample was progressively heated and measured at temperatures ranging from 25 ºC to 150 ºC, and then cooled down again to room temperature, showing no modifications from the initial spectrum.*

Photoreactivity

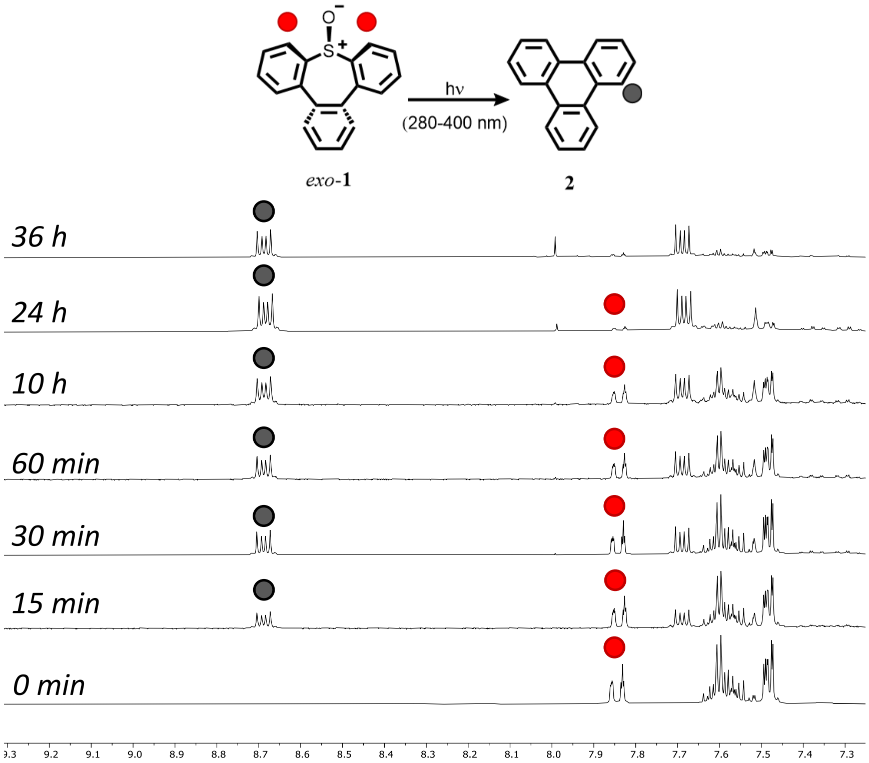

*Figure S25. Photoinduced SO-extrusion of the thiepine S-oxide exo-**1** monitored by ^{1}H-NMR (300 MHz) in CD_2Cl_2 (δ ppm), upon UV-light irradiation (280-400 nm). The consumption of exo-**1** was monitored by the disappearance of the signal at 7.83 ppm (red dots), while the formation of triphenylene was confirmed by the growth of the multiplet at 8.70 ppm (grey dots).*

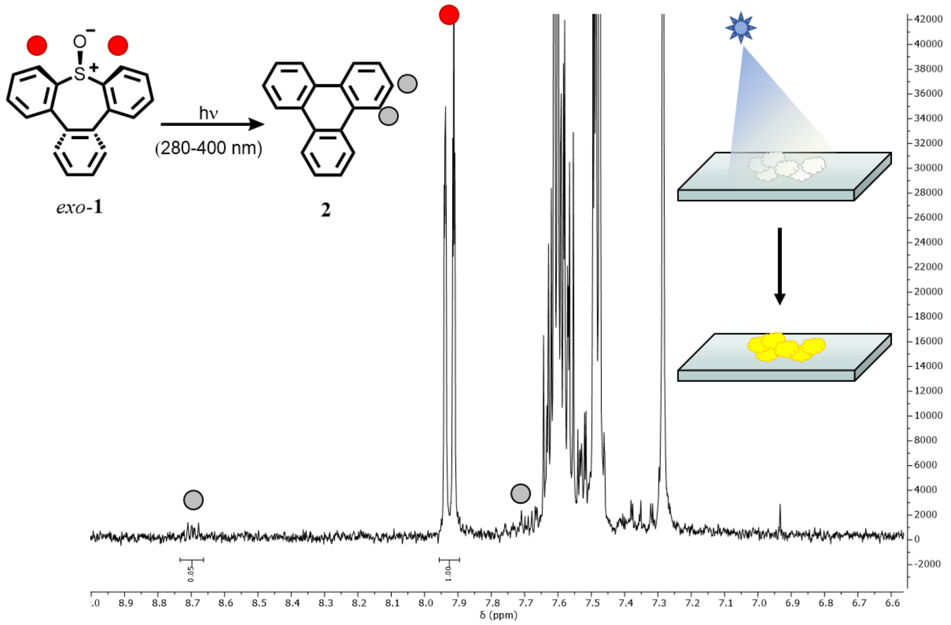

*Figure S26. ^{1}H-NMR spectrum (300 MHz) in $CDCl_3$ of exo-**1** (red dot), after its irradiation (280-400 nm) as a powder during 6 hours. The powder turned yellow, as the resulting NMR sample solution, however only ca. 2% of triphenylene **2** (grey dots) was observed thereafter.*

6.2 Investigations by UV-vis absorption spectroscopy

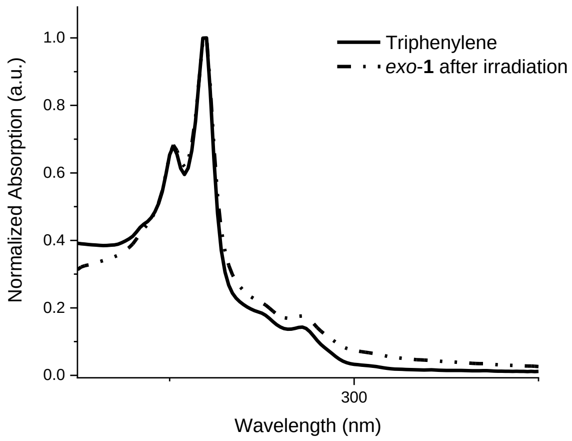

Figure S27. UV-vis absorption spectra in CH_2Cl_2 (10^{-5} M) of freshly prepared triphenylene **2** *(solid line) and of a sample of tribenzothiepine S-oxide exo-***1** *submitted to UV-light irradiation (280-400 nm) for 225 seconds (dotted line). The perfect match of both spectra confirmed the SO-extrusion of the thiepine S-oxide derivative to form triphenylene* **2** *as final product.*

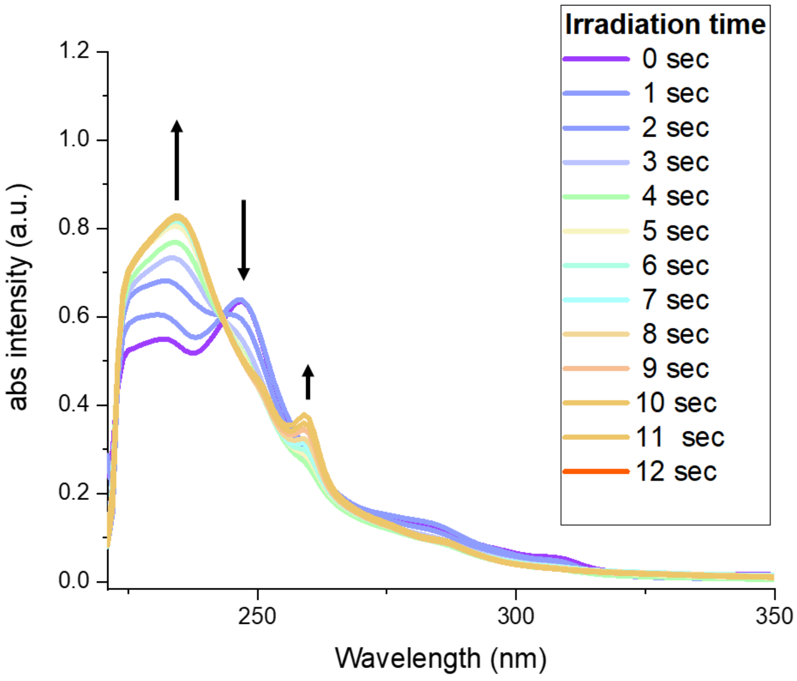

*Figure S28. Photoisomerisation of endo-***1** *(purple) into exo-***1** *(orange) in CH_2Cl_2 solutions (10^{-5} M) upon UV-light irradiation (280-400 nm), monitored by UV-vis absorption spectroscopy. The photochemical reaction succeeded in 12 seconds, as was observed from the increasing peak at 234 nm ascribed to the exo-sulfoxide exo-***1***, while the characteristic maximum of endo-***1** *at 247 nm decreased. In contrast with the ^{1}H-NMR experiments, traces of triphenylene* **2** *were detected in the last stage illuminations of the sample.*

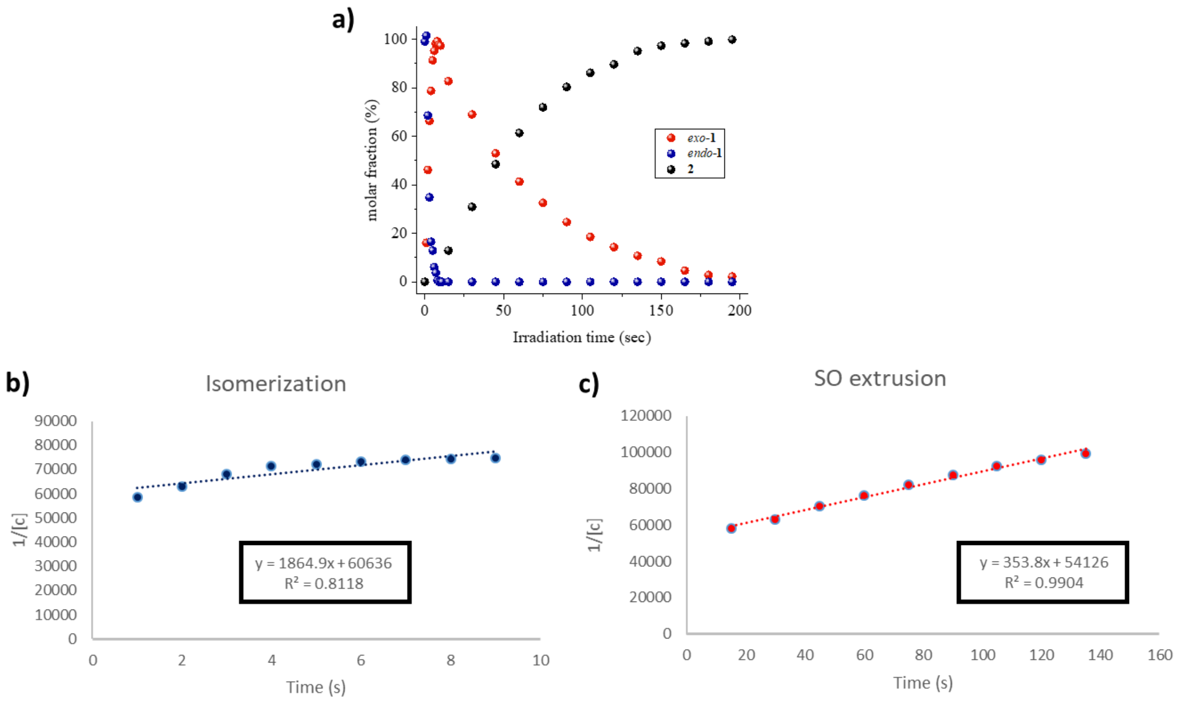

*Figure S29. a) Kinetic profile for the reaction of sulfoxide endo-**1** (blue dots, initial concentration of ca. 10^{-5} M in CH_2Cl_2) upon photoirradiation (280-400 nm), involving first the isomerisation into the exo-**1** intermediate (red dots) and subsequent SO extrusion releasing triphenylene **2** (black dots). b,c) Second-order plot of the experimental isomerisation (b) and SO-extrusion (c), extracted from the kinetic profile. Linear regression was applied to the 1/[c] vs Time plots in order to extract the kinetic constant (black squares, in L/mol·s).*

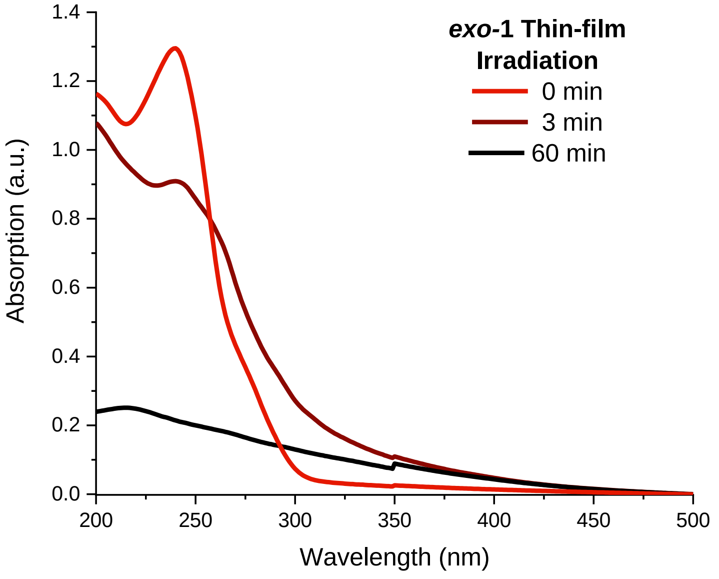

*Figure S30. Thin-film UV-vis absorption spectra of exo-**1** onto quartz, before (red curve) and after irradiation by UV-light (280-400 nm). After 3 minutes (brown curve), only a red-shifted shoulder was observed. Increasing the irradiation time or the source power ended-up in a negligible spectrum, due to photobleaching or loss of the thin-film integrity.*

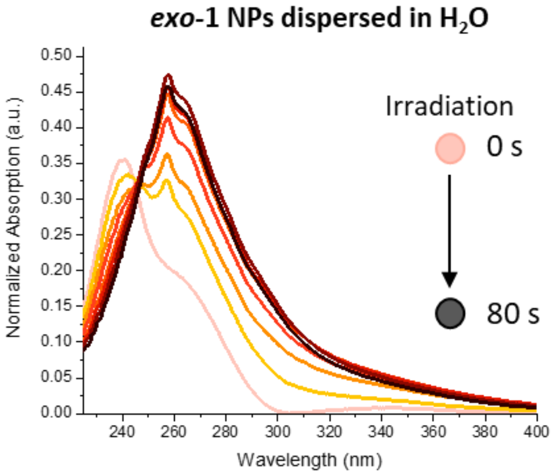

*Figure S31. UV-vis spectra of exo-**1** nanoparticles dispersed in water. Similarly to the experiment in CH_2Cl_2 solution, the photoinduced extrusion of SO can be monitored by the continuous growth of the triphenylene maximum at 257 nm and the concomitant decrease of the thiepine S-oxide exo-**1** peak at 240 nm.*

7. Theoretical calculations

The density functional theory (DFT) and time-dependent (TD)-DFT single point geometry optimisation were carried out with Gaussian16[10] at the PBE1PBE/6-311+g(d,p) level applying the D3 version of Grimme's dispersion. The transition states calculations were performed using the Berny geometry optimisation and corroborate with the QST3 method and the IRC reaction path request. The absorption spectra were computed with full TD-DFT calculations at the ωB97X-D/6-31G(d,p), applying the polarisable continuum mode (PCM) using a screening dielectric constant, with the role of solvent polarity being addressed elsewhere.

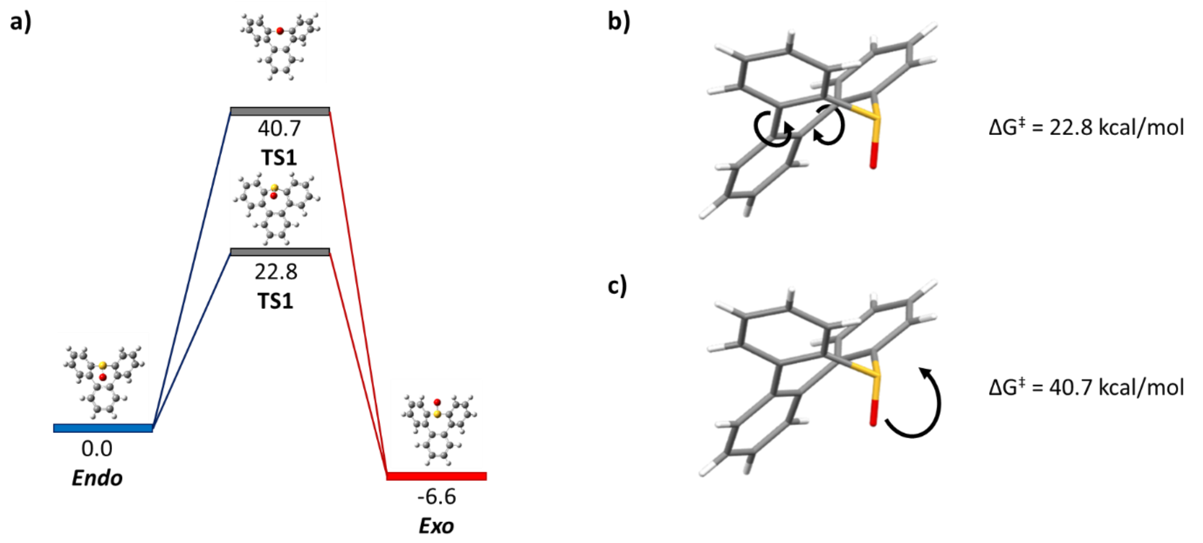

*Figure S32. Energy diagram of the isomerisation paths for the conversion of endo-**1** into exo-**1** obtained through DFT theoretical calculations (a). An activation energy of $\Delta G^{\ddagger}$ = 22.8 kcal/mol was found for this irreversible transformation from the endo-isomer to the exo-counterpart via simultaneous rotation of the two biaryl single bonds of the tribenzothiepine scaffold (b), while an activation energy of 40.7 kcal/mol was calculated for the sulfoxide epimerisation occurring through a change of hybridisation at the sulfur atom (c).*

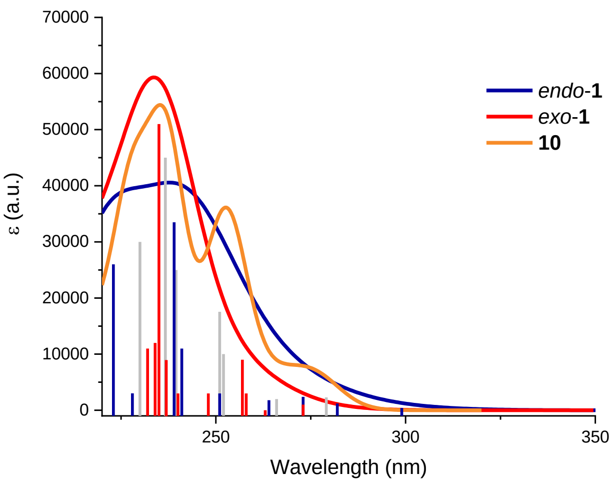

*Figure S33. TD-DFT simulated UV-vis spectra of the tribenzothiepine S-oxides exo-**1** (red curve) and endo-**1** (blue curve), and of the corresponding sulfone **10** (orange curve). Vertical lines correspond to the excited state transitions and the representative oscillator strength.*

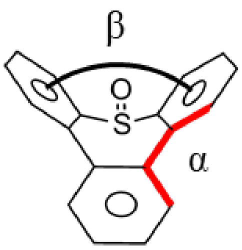

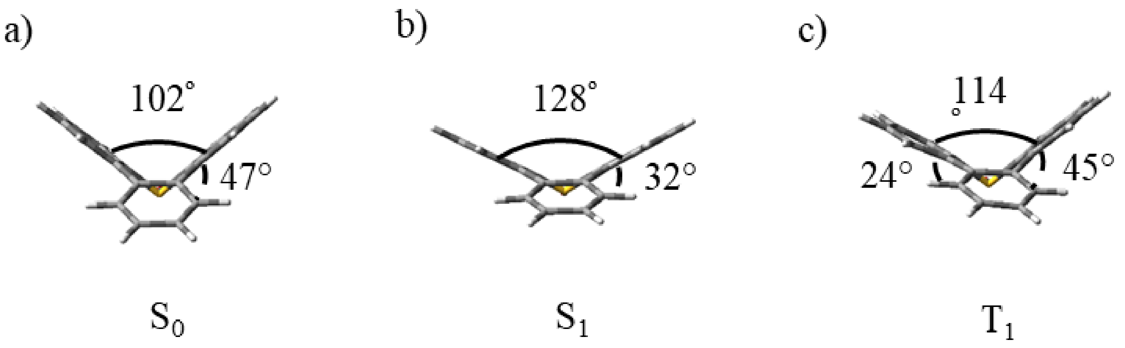

d)

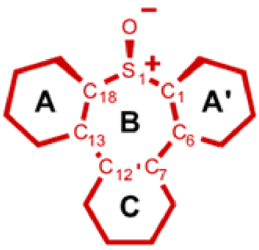

	S_0 (Å)	S_1 (Å)	T_1 (Å)
C1-S1	1.81	1.75	1.78
C1-C6	1.39	1.42	1.39
C6-C7	1.48	1.44	1.47
C7-C12	1.41	1.45	1.47
C12-C13	1.48	1.44	1.40
C13-C18	1.40	1.42	1.45
C18-S1	1.81	1.74	1.78

	exo-**1**		
	HOMA		
	S_0	S_1	T_1
**A**	0.991	0.860	0.992
**A'**	0.991	0.860	0.435
**B**	-0.118	0.366	-0.041
**C**	0.965	0.626	0.471

*Figure S34. a) DFT-optimised geometry of exo-**1** in the ground state (S_0) showing a dihedral angle of 47° in the biphenyl system (α angle, depicted on the scheme above) and 102° between the two aryls vicinal to the sulfoxide moiety (β angle). These values are in agreement with the single crystal X-ray diffraction analysis displaying angles of α=49° and β=103° (Fig. S49). b) At the TD-DFT level, geometry optimisation of the singlet first excited state (S_1) of exo-**1** shows a dihedral angle α=32° in the biphenyl moiety and a value of β=128°. This picture emphasises the planarisation of the thiepine exo-S-oxide upon excitation. This phenomenon agrees with the large Stokes shift and poor photoluminescence of the molecule due to the molecular reorganisation. c) The TD-DFT optimised geometry of the first triplet state (T_1) depicts lower planarisation than S_1, with an angle β=114°. Additionally, the breaking of symmetry yields two different α values of 24° and 45°, respectively. In comparison with the theoretical calculations reported for the dibenzo[b,f]thiepine,[11] the tribenzothiepine seven-membered ring does not become fully planar after excitation, probably because of the hindrance between hydrogens in the biphenyl systems. The view chosen to display the above-mentioned angles hampers the observation of the sulfoxide oxygen atom that remains hidden behind the sulfur atom. d) Bond lengths in the 7-membered ring (centre) and HOMA values for the four rings (right) of the computed geometries of exo-**1** in its S_0, S_1 and T_1 states.*

*Table S1. Selected angle values of optimised geometries of endo-**1** in its ground state (S_0), first singlet (S_1) and triplet (T_1) excited states and radical cation (+1) state, to evaluate the hybridisation of the sulfur atom in the sulfoxide moiety. The angles a, b, c and Φ are defined on the scheme, and "ave" represents the average value of a, b and c. A larger average value "ave" combined with a smaller absolute value of the dihedral angle Φ indicate higher sp2 hybridisation.*

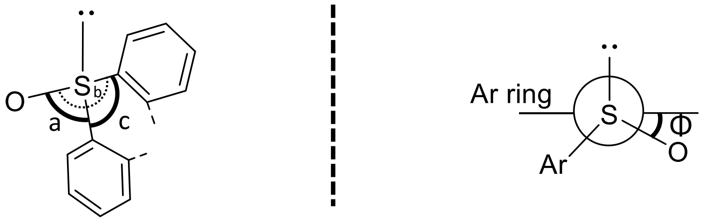

	a	*b*	*c*	*ave*	Φ
S_0	110.00	109.69	95.69	105.13	-63.82
S_1	114.24	114.26	106.84	111.78	-54.86
T_1	108.89	108.50	95.04	104.14	-66.32
+*1*	103.85	103.84	97.06	101.58	-71.15

Computed NMR shifts were determined using the GIAO method with Gaussian16[10] at the WP04/cc-pVDZ level of theory and they were empirically scaled following the reported method of Tantillo *et al.*[12,13] The ^1H-^1H coupling constants were simulated at the B3LYP/6-31G(d,p) level of theory. In both cases, the influence of chloroform was included by the PCM model. ^1H-NMR shifts and coupling constants were extracted using scripts available at the CHEmical SHIft REpository (CHESHIRE).[14] The simulated ^1H-NMR spectra were generated in MestReNova 14.0.1 using the spin simulation tool. ^{13}C-NMR shifts and spectra were extracted using the software Multiwfn[15,16] and eventually depicted with Origin.

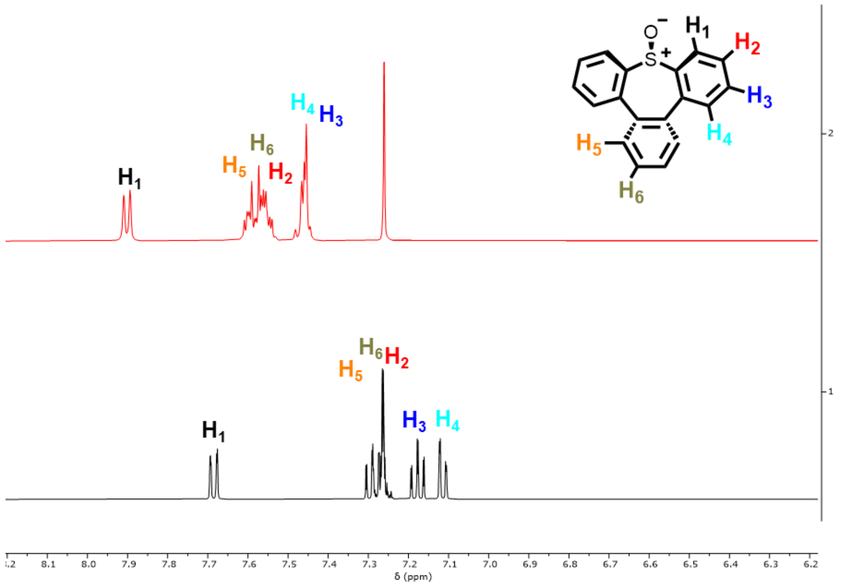

*Figure S35. Aromatic region of the calculated ^1H-NMR (0.5 Hz line width) spectrum of exo-**1** (bottom) and of the experimental spectrum (top) of the pure compound.*

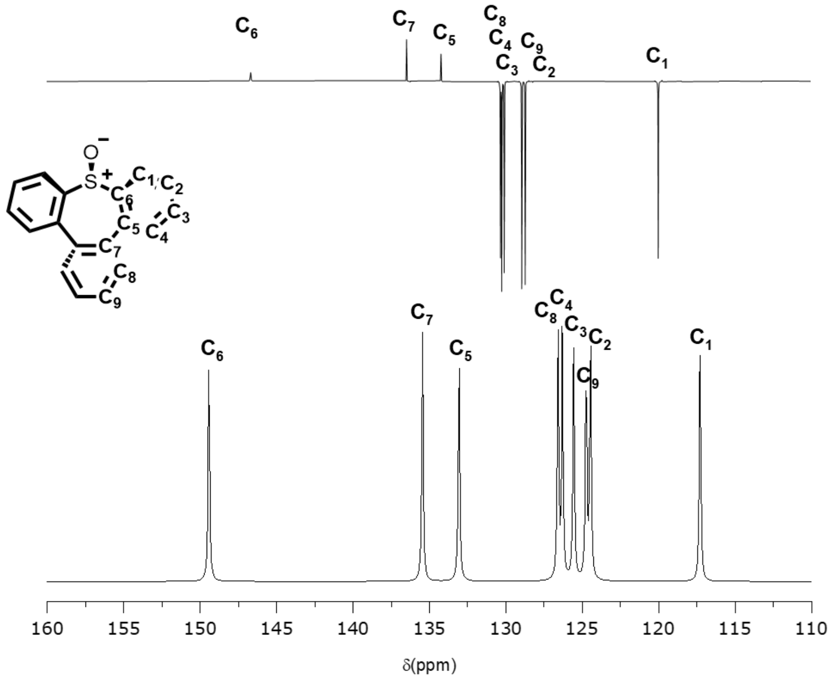

*Figure S36. 110-160 ppm region of the calculated ^{13}C-NMR spectrum of exo-**1** (bottom) and of the experimental ^{13}C-Jmod-NMR spectrum (top) of the pure compound.*

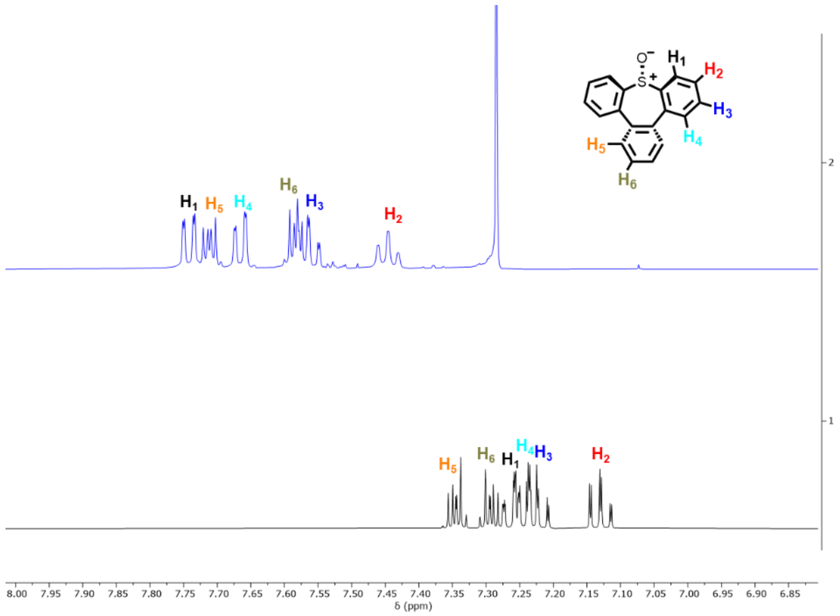

*Figure S37. Aromatic region of the calculated ^{1}H-NMR (0.5 Hz line width) spectrum of endo-**1** (bottom) and of the experimental spectrum (top) of the pure compound.*

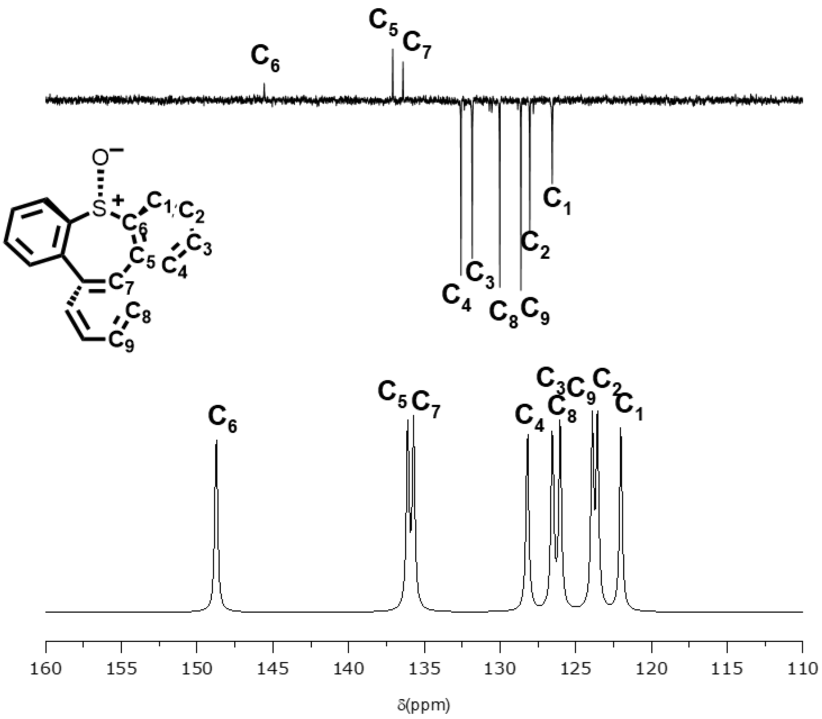

*Figure S38. 110-160 ppm region of the calculated ^{13}C-NMR spectrum of endo-**1** (bottom) and of the experimental ^{13}C-Jmod-NMR spectrum (top) of the pure compound.*

8. Solid-state characterisation

8.1 Single crystal X-ray diffraction

CCDC-2463548 to CCDC-2463552 contain the supplementary crystallographic data for these compounds. These data can be obtained free of charge from The Cambridge Crystallographic Data Centre via https://www.ccdc.cam.ac.uk/structures.

Crystal data and structure refinement of compounds *exo*-**1**, *endo*-**1**, **5**, **9** and **10** are displayed in Table S2. Selected bond lengths and angles are gathered in Table S3 for direct comparison of the tribenzothiepine scaffolds bearing different oxidation degrees. Eventually, additional information for each compound regarding the obtained crystals, corresponding unit cell and short-contact interactions is detailed in Tables S4-S7 and Figures S39-S50. Molecular graphics were created by Mercury software.[17]

Structure Tables

*Table S2. Crystal data and structure refinement of compounds exo-**1**, endo-**1**, **5**, **9** and **10**.*

	exo-**1**	*endo*-**1**	**5**	**9**	**10**
CCDC number	2463548	2463549	2463550	2463551	2463552
Empirical formula	$C_{18}H_{12}OS$	$C_{18}H_{12}OS$	$C_{18}H_{12}F_2$	$C_{18}H_{12}S$	$C_{18}H_{12}O_2S$
Formula weight	276.34	276.34	266.28	260.34	292.34
Crystal system	monoclinic	orthorhombic	monoclinic	monoclinic	monoclinic
Space group	$P2_1/c$	$Pna2_1$	$P2_1/c$	$P2_1/n$	$C2/c$
a [Å]	12.3321(8)	36.535(7)	14.7655(6)	7.4380(6)	25.6615(17)
b [Å]	15.8573(11)	7.6974(15)	6.7441(3)	21.9521(18)	8.0439(5)
c [Å]	13.8659(8)	9.6111(18)	14.7616(6)	16.1226(15)	13.7786(8)
α [°]	90	90	90	90	90
β [°]	102.912(3)	90	114.8414(12)	92.945(3)	95.872(3)
γ [°]	90	90	90	90	90
Volume [Å^3]	2643.0(3)	2702.8(9)	1333.95(10)	2629.0(4)	2829.2(3)
Z	8	8	4	8	8
ρ_{calc} [gcm^{-3}]	1.389	1.358	1.326	1.315	1.373
μ [mm^{-1}]	0.236	0.231	0.095	0.227	0.229
F(000)	1152	1152	552	1088	1216
Crystal size [mm^3]	0.200×0.250×0.300	0.040×0.050×0.150	0.200×0.250×0.400	0.030×0.050×0.160	0.120×0.200×0.400
Crystal colour	colourless	colourless	colourless	colourless	colourless
Crystal shape	prism	stick	plate	needle	plate
Reflections collected	121855	80833	41178	75403	69089
Independent reflections	6597<br>R_{int} = 0.0592<br>R_{sigma} = 0.0203	6793<br>R_{int} = 0.1381<br>R_{sigma} = 0.0731	3145<br>R_{int} = 0.0350<br>R_{sigma} = 0.0156	5475<br>R_{int} = 0.1640<br>R_{sigma} = 0.0904	3521<br>R_{int} = 0.0539<br>R_{sigma} = 0.0206
Completeness to θ = 25.242°	99.7	99.9	99.8	100.0	99.5
Data / Restraints / Parameters	6597 / 0 / 361	6793 / 1 / 361	3145 / 2 / 202	5475 / 0 / 343	3521 / 0 / 190
Goodness-of-fit on F^2	1.026	1.032	1.031	1.031	1.079
Final R indexes [I≥2σ(I)]	R_1 = 0.0371<br>wR_2 = 0.0954	R_1 = 0.0542<br>wR_2 = 0.0957	R_1 = 0.0357<br>wR_2 = 0.0846	R_1 = 0.0599<br>wR_2 = 0.0878	R_1 = 0.0432<br>wR_2 = 0.1138
Final R indexes [all data]	R_1 = 0.0472<br>wR_2 = 0.1027	R_1 = 0.1006<br>wR_2 = 0.1122	R_1 = 0.0469<br>wR_2 = 0.0928	R_1 = 0.1330<br>wR_2 = 0.1074	R_1 = 0.0506<br>wR_2 = 0.1197
Largest peak/hole [eÅ^{-3}]	0.38/−0.32	0.26/−0.37	0.20/−0.14	0.26/−0.31	0.42/−0.36
Extinction coefficient	---	---	0.0091(11)	---	---
Flack X parameter	---	0.02(5)	---	---	---

*Table S3. Selected bond lengths and angles for compounds exo-**1**, endo-**1**, **9** and **10**, with ORTEP drawn at 50% probability level (hydrogen atoms omitted for clarity).*

	***exo*-1**	***endo*-1**	**9**	**10**
CCDC number	2463548	2463549	2463551	2463552
**Selected bond lengths [Å]**				
S1-O1	1.493(1)	1.483(4)	---	1.441(1)
S1-O2	---	---	---	1.441(1)
S1-C18	1.795(1)	1.787(4)	1.774(3)	1.756(1)
S1-C1	1.795(2)	1.793(4)	1.784(3)	1.762(2)
C1-C6	1.398(2)	1.403(6)	1.405(4)	1.400(2)
C6-C7	1.488(2)	1.483(6)	1.485(4)	1.490(2)
C7-C12	1.412(2)	1.401(6)	1.409(4)	1.409(2)
C12-C13	1.488(2)	1.494(6)	1.491(4)	1.486(2)
C13-C18	1.402(2)	1.402(6)	1.399(4)	1.407(2)
**Selected angles [°]**				
O1-S1-C18	107.4(1)	108.7(2)	---	108.9(1)
O1-S1-C1	107.3(1)	110.0(2)	---	109.1(1)
O2-S1-C18	---	---	---	109.4(1)
O2-S1-C1	---	---	---	110.2(1)
O2-S1-O1	---	---	---	117.6(1)
C18-S1-C1	93.2(1)	95.7(2)	97.7(1)	100.3(1)
C6-C1-S1	118.9(1)	122.9(4)	120.5(2)	118.9(1)
C1-C6-C7	121.9(1)	123.7(4)	123.2(3)	124.2(1)
C12-C7-C6	122.9(1)	124.5(4)	124.5(3)	123.7(1)
C7-C12-C13	123.5(1)	123.6(4)	124.5(3)	124.5(1)
C18-C13-C12	122.5(1)	124.1(4)	123.2(3)	123.7(1)
C13-C18-S1	119.0(1)	122.6(3)	120.6(2)	119.4(1)

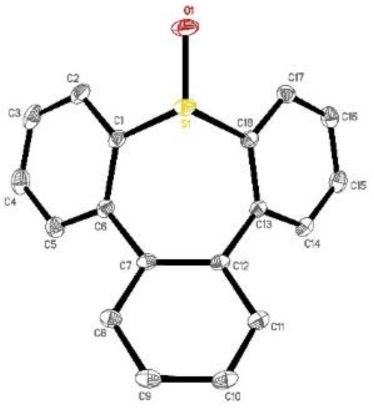

exo-**1**

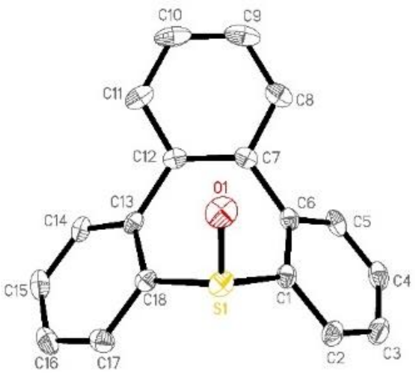

endo-**1**

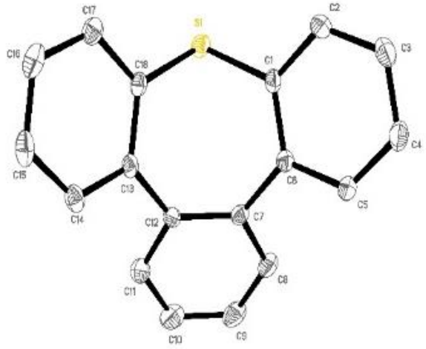

9

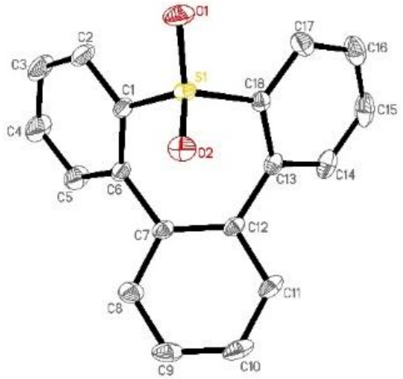

10

*Table S4. Short contacts involving S and O detected in structures of compounds exo-**1**, endo-**1**, **9** and **10**, and short contacts involving F detected in structure **5**.*

D—H•••A	D—H [Å]	H•••A [Å]		D•••A [Å]	D—H•••A [°]	Symmetry codes
			exo-**1**			
C8—H8•••S2	0.95	2.84		3.415(2)	120	-1+*x*, *y*, *z*
C10—H10•••O1	0.95	2.57		3.438(2)	151	1-*x*, -1/2+*y*, 1/2-*z*
C28—H28•••O2	0.95	2.55		3.320(2)	139	2-*x*, 1/2+*y*, 1/2-*z*
			endo-**1**			
C16—H16•••O1	0.95	2.41		3.251(7)	147	1–*x*, 1–*y*,1/2 +*z*
C34—H34•••O2	0.95	2.46		3.319(7)	151	3/2–*x*, –1/2+*y*, –1/2+z
			**5**			
C2—H2•••F2	0.95	2.53		3.454(2)	163	*x*, 1/2–*y*, –1/2+*z*
			**10**			
C2—H2•••O1	0.95	2.43		3.263(2)	146	1–*x*, *y*, 3/2–*z*
C16—H16•••O2	0.95	2.47		3.192(2)	133	*x*, –1+*y*, *z*

*Table S5. Geometrical parameters (Å, °) for C–H···π interactions detected in structures of compounds exo-**1**, endo-**1**, and **9**.*

C—H(i)•••Cg(i)	H···Cg [Å]	C···Cg [Å]	C–H···Cg [°]	H-Perp	Gamma
		exo-**1**			
C21—H21•••Cg(3)[#1]	2.87	3.6534(18)	141	–2.84	8.35
C27—H27•••Cg(12)[#2]	2.99	3.7655(16)	140	2.98	3.97
C34—H34•••Cg(2)[#3]	2.82	3.5393(19)	134	–2.74	13.54
		endo-**1**			
C2—H2•••Cg(12)[#4]	2.97	3.746(5)	140	–2.88	14.32
C3—H3•••Cg(14)[#4]	2.78	3.670(6)	156	–2.73	11.42
C20—H20•••Cg(1)[#5]	2.86	3.627(5)	138	–2.75	15.84
C21—H21•••Cg(3)[#5]	2.72	3.498(5)	140	2.69	7.65
C26—H26•••Cg(2)[#6]	2.93	3.566(5)	125	–2.83	15.10
C28—H28•••Cg(1)[#7]	2.97	3.345(5)	105	2.90	12.48
		**9**			
C29—H29•••Cg(2)[#8]	2.97	3.819(3)	149	2.90	12.60

Centroids:
For compound *exo*-**1**, Cg(3) is the centroid of the ring (C13–C18), Cg(12) is the centroid of the ring (C19–C24), and Cg(2) is the centroid of the ring (C7–C12).
For compound *endo*-**1**, Cg(12) is the centroid of the ring (C19–C24), Cg(14) is the centroid of the ring (C31–C36), Cg(1) is the centroid of the ring (C1–C6), Cg(3) is the centroid of the ring (C13–C18), Cg(2) is the centroid of the ring (C7–C12).
For compound **9**, Cg(2) is the centroid of the ring (C7–C12).

Symmetry codes:
#1: *1–x, –1/2+y, 1/2–z*; #2: *x, 3/2–y, –1/2+z*; #3: 1+*x, 3/2–y*, 1/2+*z;* #4: *x, y*, 1+*z;* #5: *x*, 1+*y*, –1+*z;* #6: *x*, 1+*y, z;* #7: *x, y, z*, #8: *3/2–x, –1/2+y, 1/2–z*.

*Table S6. Geometrical parameters (Å, °) for C–F···π interactions detected in structure of compound **5**.*

C—F(i)•••Cg(i)	F···Cg [Å]	C···Cg [Å]	C–F···Cg [°]	H-Perp	Gamma
**5**					
C18'—F2'•••Cg(1)$^{\#1}$	3.912(17)	4.9850(16)	143.4(10)	-3.719	18.10

For complex **5**, Cg(1) is the centroid of the ring (C1–C6).
Symmetry codes: #1: *x*, 1+*y*, *z*.

*Table S7. Geometrical parameters (Å, °) for π···π interactions detected in structures of compounds exo-**1**, **5**, **9** and **10**.*

Cg(i)•••Cg(j)	Cg···Cg [Å]	α [°]	β [°]	γ [°]	Cg(i)_Perp	Cg(j)_Perp	Slippage
exo-**1**							
Cg(1)•••Cg(12)$^{\#1}$	3.8205(9)	11.22(7)	22.8	19.9	3.5930(6)	3.5217(6)	1.481
Cg(13)•••Cg(14)$^{\#2}$	3.9406(9)	8.03(7)	29.4	24.4	3.5874(6)	3.4348(7)	1.931
**5**							
Cg(4)•••Cg(4)$^{\#3}$	3.8050(8)	0.02(7)	20.3	20.3	3.5683(6)	3.5682(6)	1.321
**9**							
Cg(3)•••Cg(12)$^{\#4}$	3.7809(17)	2.76(14)	24.5	24.1	3.4504(12)	3.4417(12)	1.565
**10**							
Cg(1)•••Cg(1)$^{\#5}$	3.8357(1)	0.02(10)	13.3	13.3	3.7335(8)	3.7335(8)	0.879

Centroids:
For compound *exo*-**1**, Cg(1), Cg(12), Cg(13) and Cg(14) are the centroids of the rings (C1–C6), (C19–C24), (C25–C30) and (C31–C36), respectively.
For compound **5**, Cg(4) is the centroid of the ring (C13–C18).
For compound **9**, Cg(3) and Cg(12) are the centroids of the rings (C13–C18) and (C19–C24), respectively.
For compound **10**, Cg(1) is the centroid of the ring (C1–C6).

Symmetry codes:
#1: –1–*x*, 1/2+ *y*, 1/2–*z*; #2: 1+*x*, *y*, *z*; #3: 1–*x*, 1–*y*, 1–*z*, #4: *x*, *y*, *z*, #5: 1–*x*, 2–*y*, 1–*z*.

exo-tribenzo[*b*,*d*,*f*]thiepine *S*-oxide (*exo*-1)

Crystals suitable for XRD analysis were obtained by slow evaporation of a chloroform solution.

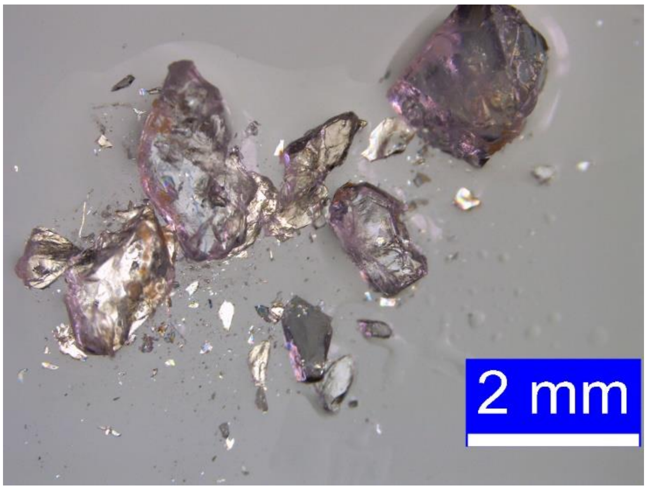

*Figure S39. Picture of the crystals of compound exo-**1**.*

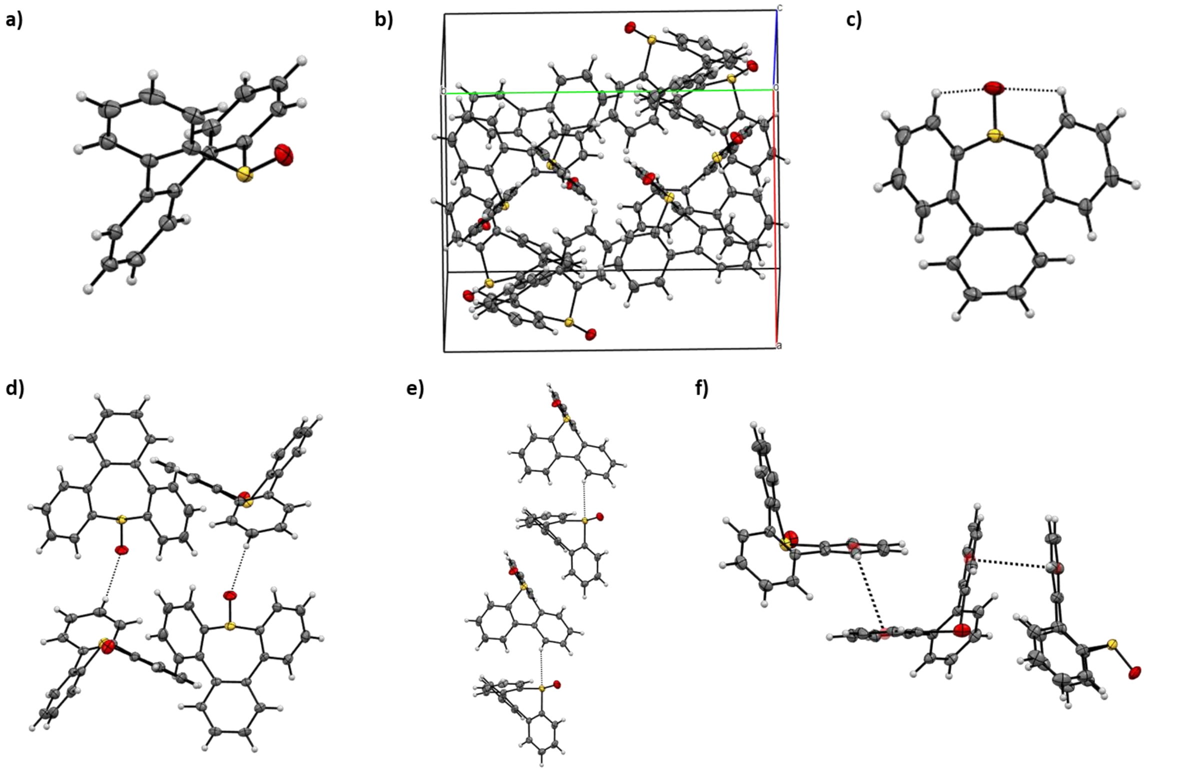

*Figure S40. a) Molecular structure and b) unit cell of compound exo-**1**. c) Intramolecular short-contact interactions between the sulfoxide oxygen and the vicinal aromatic hydrogens displaying a distance assessed as moderate hydrogen bonding. d-f) Intermolecular short-contact interactions comprising: d) O--H hydrogen bonding, e) S--H hydrogen bonding and f) π-π stacking between the aromatic rings. Thermal ellipsoids are drawn at 50% probability level. Centroids involved in short-contact interactions are represented by orange balls.*

endo-tribenzo[*b*,*d*,*f*]thiepine *S*-oxide (*endo*-1)

Crystals suitable for XRD analysis were obtained by vapour diffusion of hexane into a dichloromethane solution of *endo*-**1**.

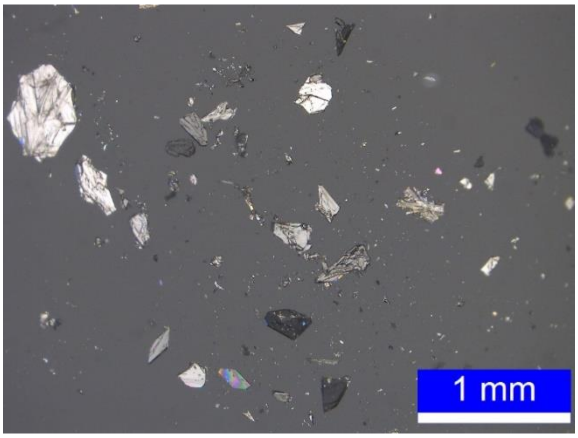

*Figure S41. Picture of the crystals of compound endo-**1**.*

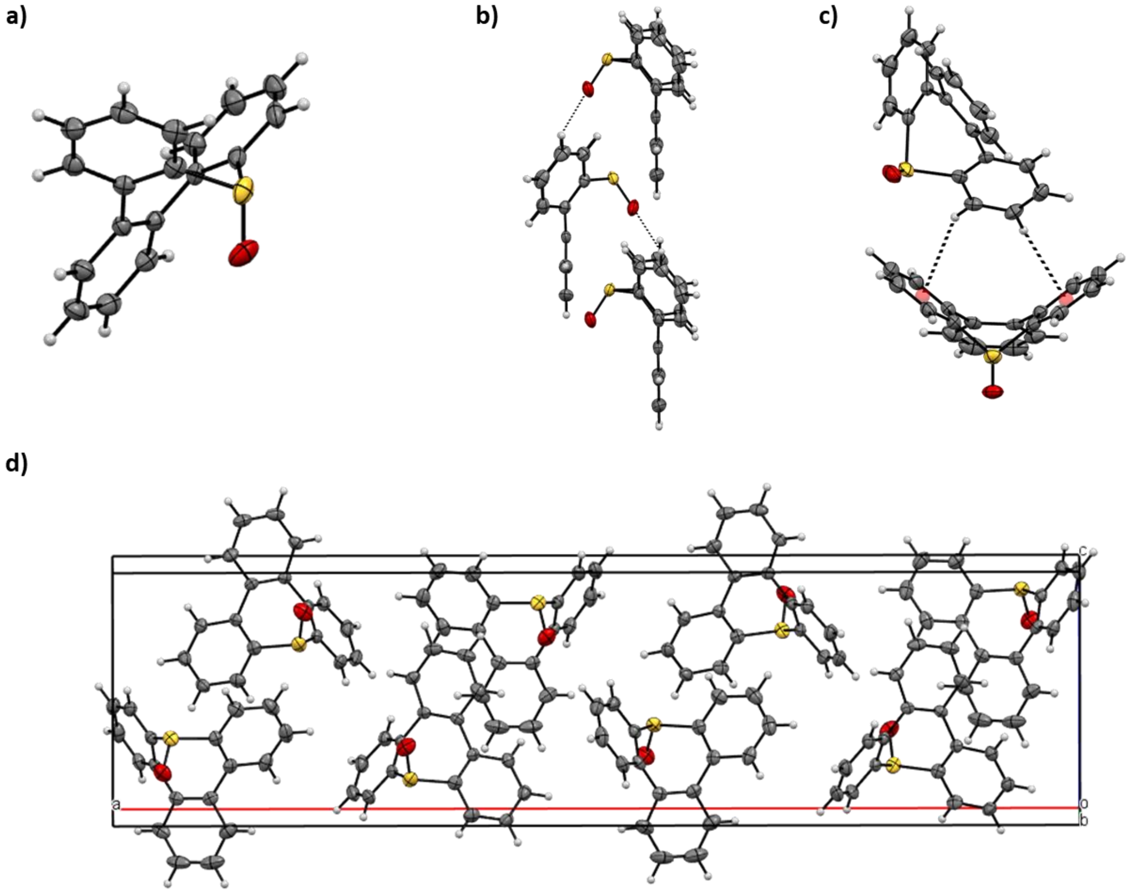

*Figure S42. a) Molecular structure and d) unit cell of compound endo-**1**. b,c) Intermolecular short-contact interactions comprising: b) O--H hydrogen bonding and c) edge-to-face C-H--π interactions. Thermal ellipsoids are drawn at 50% probability level. Centroids involved in short-contact interactions are represented by orange balls.*

2,2''-difluoro-1,1':2',1''-terphenyl (5)

Crystals suitable for XRD analysis were obtained by slow cooling of the pure compound at 5 ºC. The position of the two F atoms in the structure of **5** was found to be disordered. Several restraints (SIMU, DELU, SADI) and equal xyz and U_{ij} constraints (EXYZ and EADP) were applied to refine some moieties of the molecule and to avoid the collapse of the structure during the least-squares refinement by the large anisotropic displacement parameters.

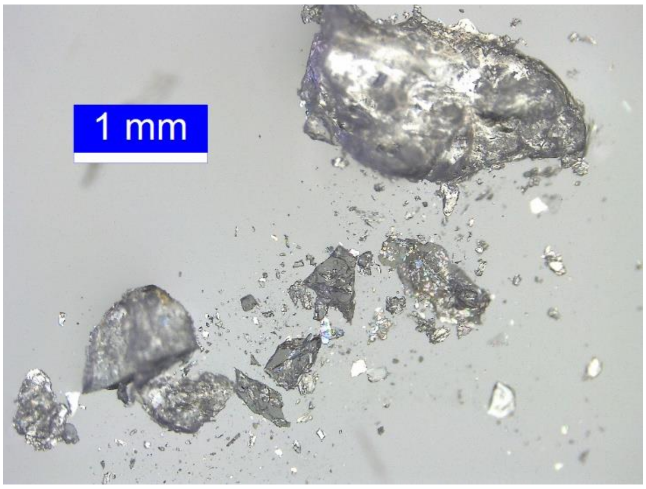

Figure S43. Picture of the crystals of compound ***5****.*

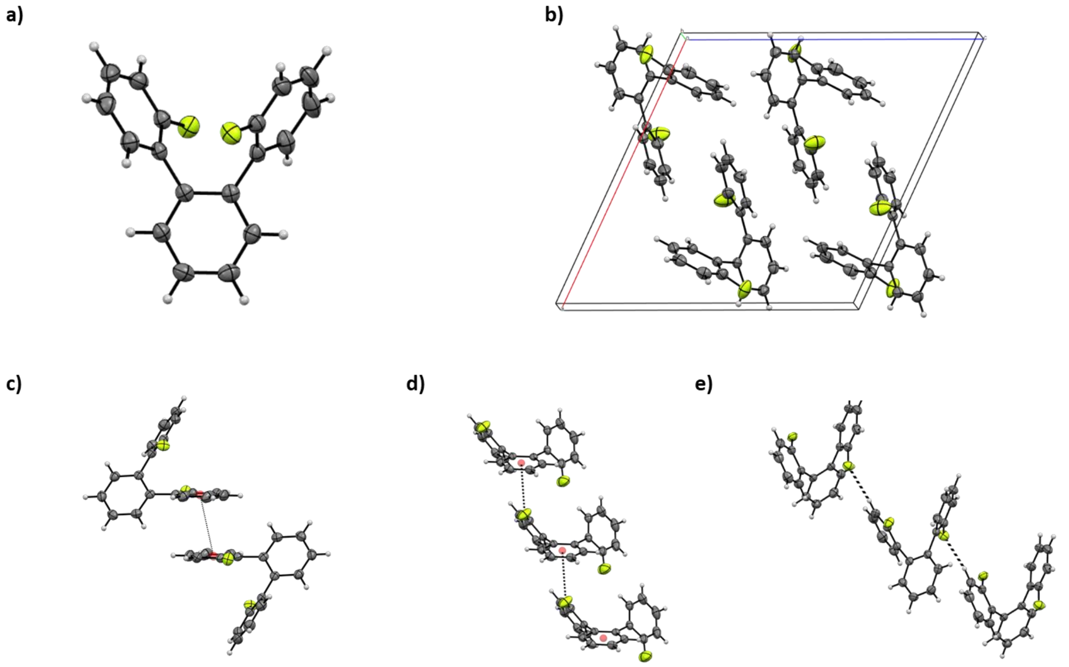

Figure S44. a) Molecular structure and b) unit cell of compound ***5****. c-e) Intermolecular short-contact interactions comprising: c) π-π stacking, d) C-F--π interactions and e) F--H hydrogen bonding. Thermal ellipsoids are drawn at 50% probability level. Centroids involved in short-contact interactions are represented by orange balls.*

tribenzo[*b*,*d*,*f*]thiepine (9)

Crystals suitable for XRD analysis were obtained by slow cooling of the pure compound at 5 ºC.

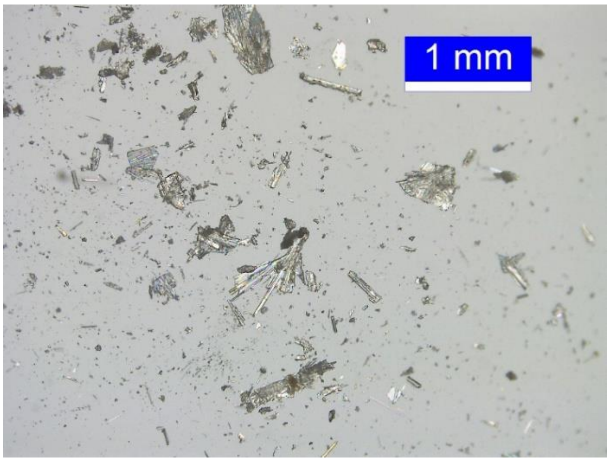

Figure S45. Picture of the crystals of compound ***9****.*

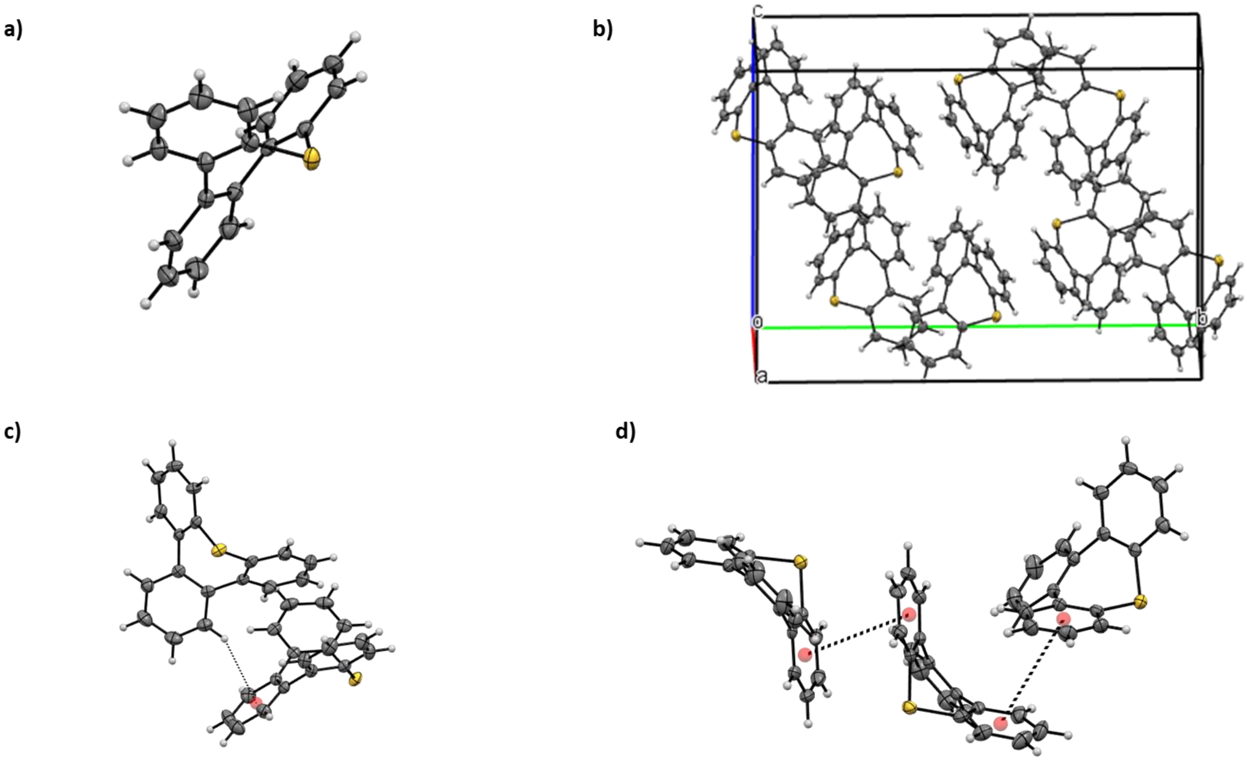

Figure S46. a) Molecular structure and b) unit cell of compound ***9****. c-d) Intermolecular short-contact interactions comprising: c) C-H--π interactions and d) π-π stacking between the aromatic rings. Thermal ellipsoids are drawn at 50% probability level. Centroids involved in short-contact interactions are represented by orange balls.*

tribenzo[*b*,*d*,*f*]thiepine *S*,*S*-dioxide (10)

Crystals suitable for XRD analysis were obtained by vapour diffusion of hexane into a dichloromethane solution of **10**.

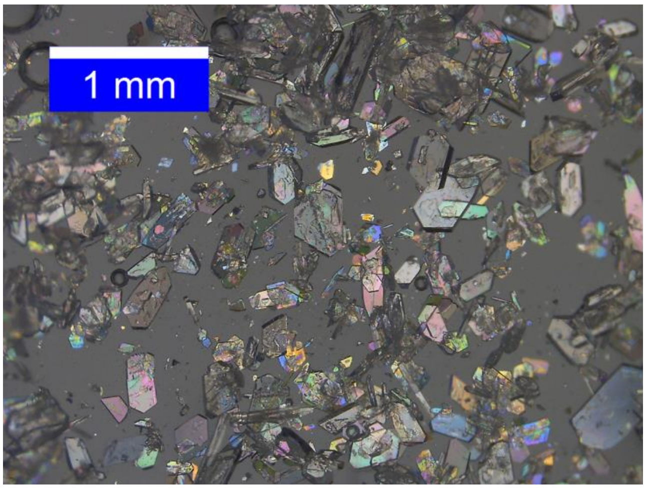

*Figure S47. Picture of the crystals of compound **10**.*

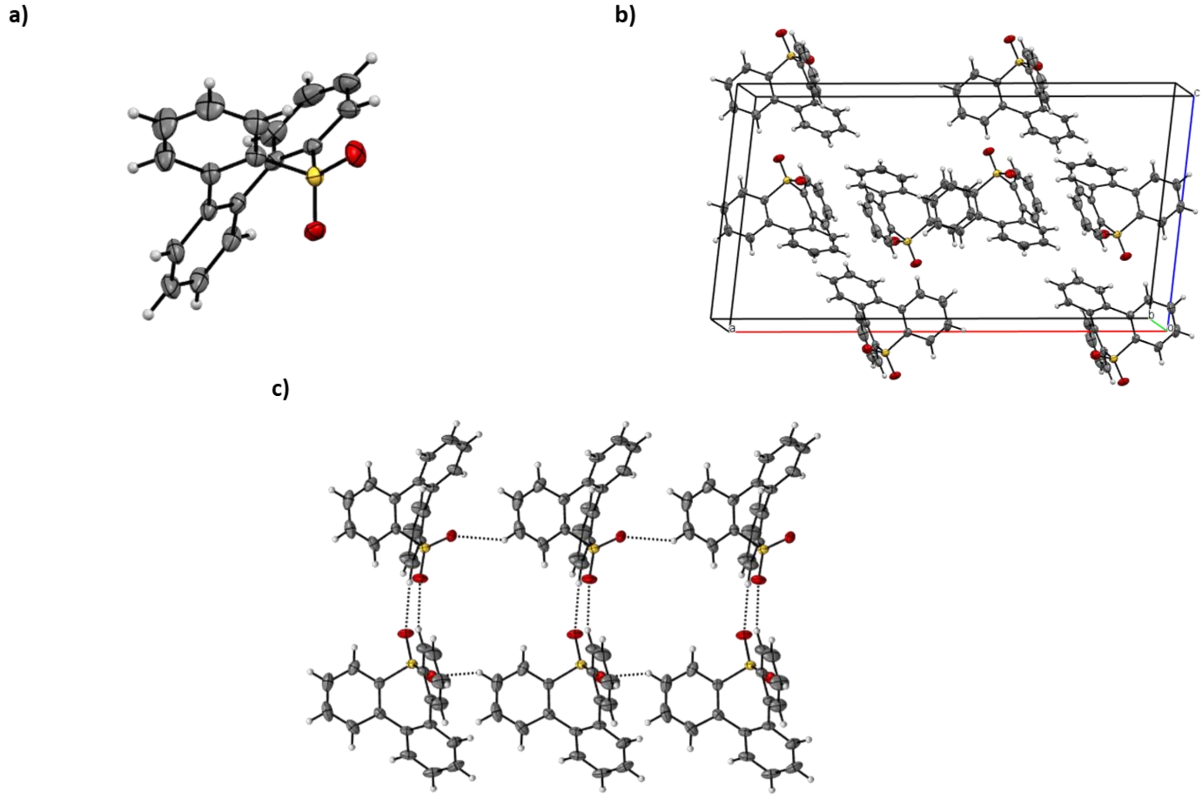

*Figure S48. a) Molecular structure and b) unit cell of compound **10**. c) Intermolecular short-contact interactions comprising O--H hydrogen bonding. Thermal ellipsoids are drawn at 50% probability level.*

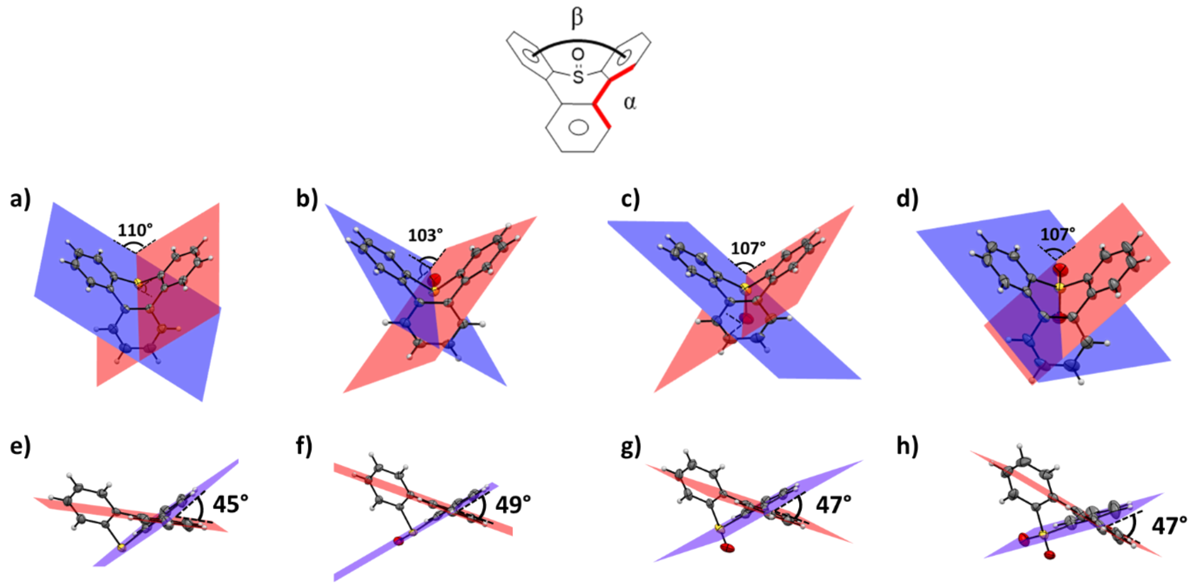

*Figure S49. Views of compounds **9** (a, e), exo-**1** (b, f), endo-**1** (c, g) and **10** (d, h) obtained from their X-ray crystal structure showing the angle **β** measured between the two planes comprising the phenyl rings vicinal to the sulfur atom (top) and the angle **α** between the two aryl rings from the biphenyl moiety (bottom). For simplicity, only one of the two possible biphenyl dihedral angles (**α**) is depicted, owing to the similar values.*

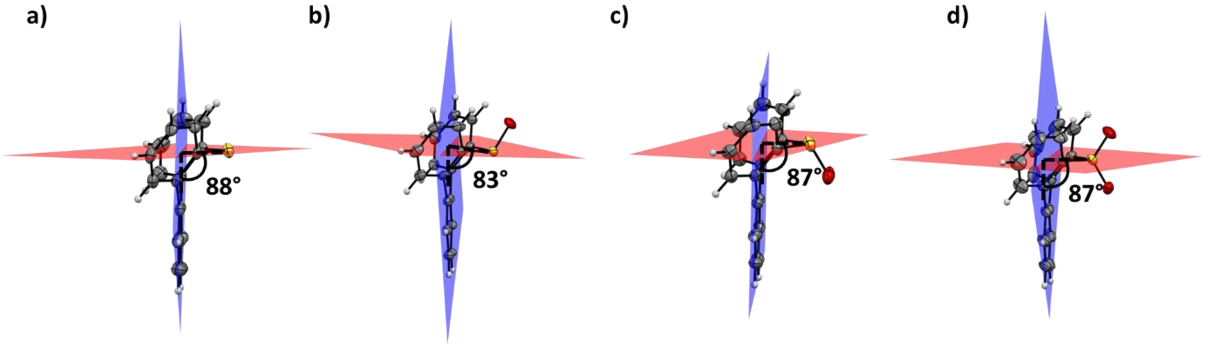

*Figure S50. Views of **9** (a), exo-**1** (b), endo-**1** (c) and **10** (d) obtained from their X-ray crystal structure, showing the angle between the plane comprising the S and the two vicinal carbon atoms, and the plane comprising the phenyl ring facing the sulfoxide moiety.*

8.2 Thermogravimetric analysis

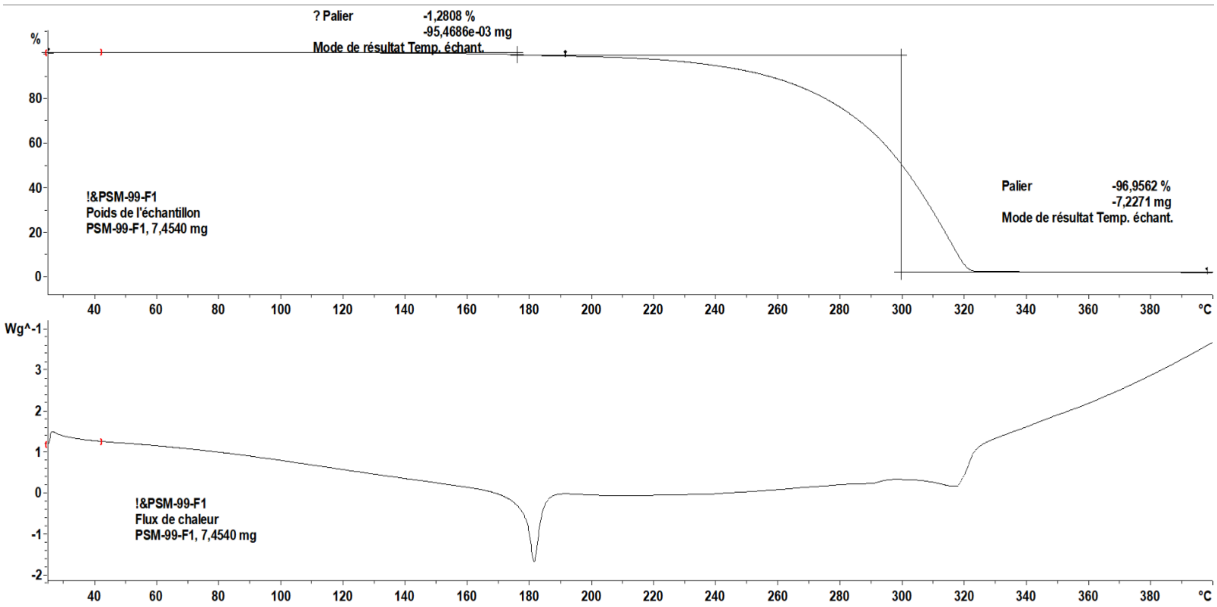

*Figure S51. Thermogravimetry analysis (TGA) of compound exo-**1**. The thermogram shows that the sample is stable until 180 ºC, thereafter it degrades until ca.320 ºC with a weight loss of 97%.*

8.3 Optical and polarised optical microscopy

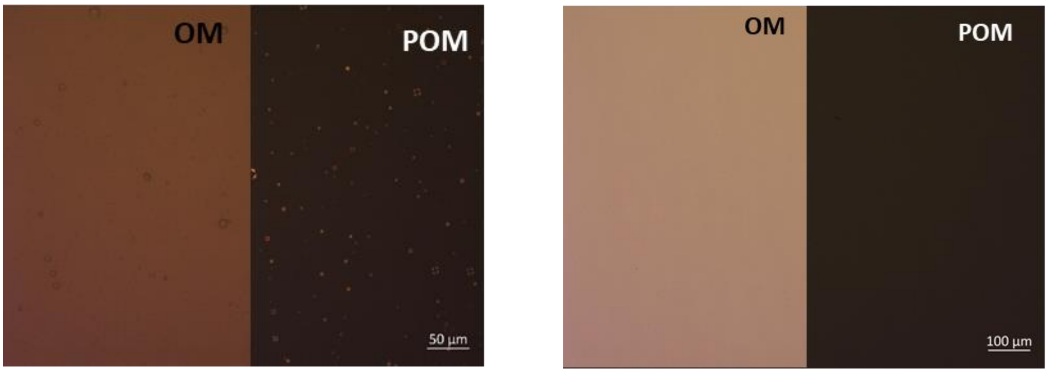

*Figure S52. Optical and polarised optical microscopy micrographs of micro- and nanoparticles of exo-**1** prepared by the precipitation process.*

8.4 Thin-films and powder X-ray diffraction

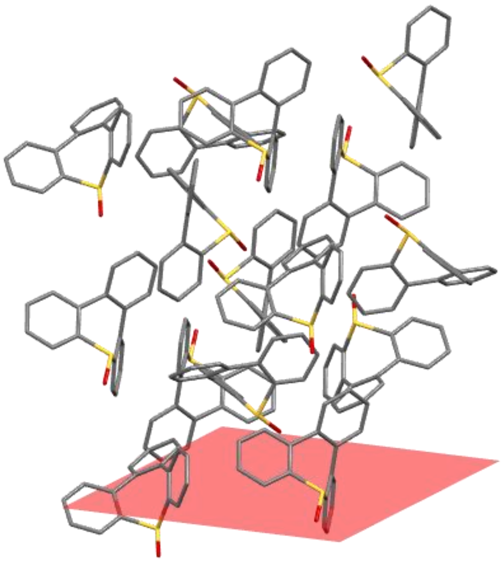

*Figure S53. Out-of-plane preferential orientation of exo-**1** in the thin-film. The plane (102), in red, appears parallel to the substrate leading the crystal growth by drop-casting deposition.*

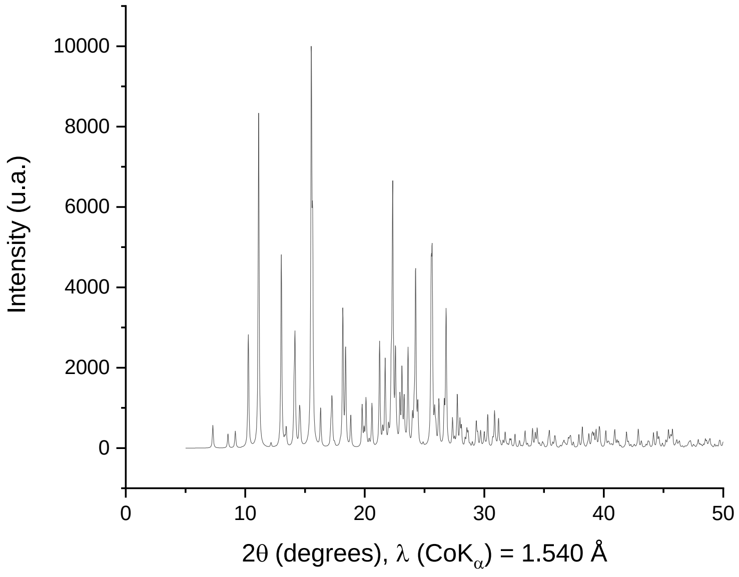

*Figure S54. X-ray diffraction 2θ/ω pattern of an exo-**1** powder sample.*

9. References

[1] S. S. Zalesskiy, V. P. Ananikov, *Organometallics* **2012**, *31*, 2302–2309.
[2] B. A. I. SADABS, *Bruker*, USA, **2008**.
[3] G. M. Sheldrick, *Acta Crystallogr. Sect. Found. Adv.* **2015**, *71*, 3–8.
[4] G. M. Sheldrick, *Acta Crystallogr. Sect. C Struct. Chem.* **2015**, *71*, 3–8.
[5] M. Shimizu, Y. Tomioka, I. Nagao, T. Hiyama, *Synlett* **2009**, 3147–3150.
[6] M. Feofanov, V. Akmetov, R. Takayama, K. Amsharov, *Angew. Chem. Int. Ed.* **2021**, *60*, 5199–5203.
[7] M. Feofanov, V. Akhmetov, R. Takayama, K. Y. Amsharov, *J. Org. Chem.* **2021**, *86*, 14759–14766.
[8] E. Dimitrijević, M. S. Taylor, *Chem. Sci.* **2013**, *4*, 3298.
[9] K. E. Yamada, I. A. Stepek, W. Matsuoka, H. Ito, K. Itami, *Angew. Chem. Int. Ed.* **2023**, *62*, e202311770.
[10] Gaussian 16, M. J. Frisch, G. W. Trucks, H. B. Schlegel, G. E. Scuseria, M. A. Robb, J. R. Cheeseman, G. Scalmani, V. Barone, G. A. Petersson, H. Nakatsuji, X. Li, M. Caricato, A. V. Marenich, J. Bloino, B. G. Janesko, R. Gomperts, B. Mennucci, H. P. Hratchian, J. V. Ortiz, A. F. Izmaylov, J. L. Sonnenberg, D. Williams-Young, F. Ding, F. Lipparini, F. Egidi, J. Goings, B. Peng, A. Petrone, T. Henderson, D. Ranasinghe, V. G. Zakrzewski, J. Gao, N. Rega, G. Zheng, W. Liang, M. Hada, M. Ehara, K. Toyota, R. Fukuda, J. Hasegawa, M. Ishida, T. Nakajima, Y. Honda, O. Kitao, H. Nakai, T. Vreven, K. Throssell, J. A. Montgomery, Jr., J. E. Peralta, F. Ogliaro, M. J. Bearpark, J. J. Heyd, E. N. Brothers, K. N. Kudin, V. N. Staroverov, T. A. Keith, R. Kobayashi, J. Normand, K. Raghavachari, A. P. Rendell, J. C. Burant, S. S. Iyengar, J. Tomasi, M. Cossi, J. M. Millam, M. Klene, C. Adamo, R. Cammi, J. W. Ochterski, R. L. Martin, K. Morokuma, O. Farkas, J. B. Foresman, and D. J. Fox, Gaussian, Inc., Wallingford CT, 2016.
[11] J. Toldo, O. El Bakouri, M. Solà, P. Norrby, H. Ottosson, *ChemPlusChem* **2019**, *84*, 712–721.
[12] M. W. Lodewyk, C. Soldi, P. B. Jones, M. M. Olmstead, J. Rita, J. T. Shaw, D. J. Tantillo, *J. Am. Chem. Soc.* **2012**, *134*, 18550–18553.
[13] M. W. Lodewyk, M. R. Siebert, D. J. Tantillo, *Chem. Rev.* **2012**, *112*, 1839–1862.
[14] T. Cheshire, P. Ramblenm, D. J. Tantillo, M. R. Siebert, M. W. Lodewyk, CHEmical SHift REpository with Coupling Constants Added Too, http://cheshirenmr.info
[15] T. Lu, F. Chen, *J. Comput. Chem.* **2012**, *33*, 580–592.
[16] T. Lu, *J. Chem. Phys.* **2024**, *161*, 082503.
[17] C. F. Macrae, I. Sovago, S. J. Cottrell, P. T. A. Galek, P. McCabe, E. Pidcock, M. Platings, G. P. Shields, J. S. Stevens, M. Towler, P. A. Wood, *J. Appl. Crystallogr.* **2020**, *53*, 226–235.
