# Supplementary material for: Light‐Induced SO Extrusion from Tribenzothiepine S‐oxides: A Precursor Approach to the Triphenylene Core
Source: Chemistry. 2025 Nov 7;31(70):e02655. doi: 10.1002/chem.202502655 (PMC12712768; doi:10.1002/chem.202502655)

checkCIF/PLATON report

Structure factors have been supplied for datablock(s) 10, 5, 9, endo_1, exo_1

THIS REPORT IS FOR GUIDANCE ONLY. IF USED AS PART OF A REVIEW PROCEDURE FOR PUBLICATION, IT SHOULD NOT REPLACE THE EXPERTISE OF AN EXPERIENCED CRYSTALLOGRAPHIC REFEREE.

No syntax errors found. CIF dictionary Interpreting this report

Datablock: exo_1

Bond precision: C-C = 0.0020 A Wavelength=0.71073

Cell:	a=12.3321(8)	b=15.8573(11)	c=13.8659(8)
	alpha=90	beta=102.912(3)	gamma=90
Temperature:	193 K		

	Calculated	Reported
Volume	2643.0(3)	2643.0(3)
Space group	P 21/c	P 21/c
Hall group	-P 2ybc	-P 2ybc
Moiety formula	C18 H12 O S	?
Sum formula	C18 H12 O S	C18 H12 O S
Mr	276.34	276.34
Dx,g cm-3	1.389	1.389
Z	8	8
Mu (mm-1)	0.236	0.236
F000	1152.0	1152.0
F000’	1153.43	
h,k,lmax	16,21,18	16,21,18
Nref	6655	6597
Tmin,Tmax	0.932,0.954	0.682,0.746
Tmin’	0.932	

Correction method= # Reported T Limits: Tmin=0.682 Tmax=0.746
AbsCorr = MULTI-SCAN

Data completeness= 0.991 Theta(max)= 28.407

R(reflections)= 0.0371(5511) wR2(reflections)= 0.1027(6597)

S = 1.026 Npar= 361

The following ALERTS were generated. Each ALERT has the format
test-name_ALERT_alert-type_alert-level.
Click on the hyperlinks for more details of the test.

Alert level C

```
PLAT910_ALERT_3_C Missing # of FCF Reflection(s) Below Theta(Min).          5 Note
                1  0  0,   1  1  0,   0  2  0,  -1  1  1,   0  1  1,
PLAT911_ALERT_3_C Missing FCF Refl Between Thmin & STh/L=    0.600          8 Report
               -3  2  1,   1  2  1,  -3  0  2,   0  0  2,   2  0  2,  -3  2  2,
               -1  2  3,  -1  3  3,
PLAT913_ALERT_3_C Missing # of Very Strong Reflections in FCF ....          7 Note
                0  2  0,  -3  2  1,   1  2  1,   2  0  2,  -3  2  2,  -1  2  3,
               -1  3  3,
```

Alert level G

```
PLAT912_ALERT_4_G Missing # of FCF Reflections Above STh/L=  0.600         45 Note
PLAT969_ALERT_5_G The 'Henn et al.' R-Factor-gap value ..........      5.442 Note
              Predicted wR2: Based on SigI**2  1.89 or SHELX Weight 10.02
PLAT978_ALERT_2_G Number C-C Bonds with Positive Residual Density.         21 Info
```

0 **ALERT level A** = Most likely a serious problem - resolve or explain
0 **ALERT level B** = A potentially serious problem, consider carefully
3 **ALERT level C** = Check. Ensure it is not caused by an omission or oversight
3 **ALERT level G** = General information/check it is not something unexpected

0 ALERT type 1 CIF construction/syntax error, inconsistent or missing data
1 ALERT type 2 Indicator that the structure model may be wrong or deficient
3 ALERT type 3 Indicator that the structure quality may be low
1 ALERT type 4 Improvement, methodology, query or suggestion
1 ALERT type 5 Informative message, check

Datablock: endo_1

Bond precision:	C-C = 0.0068 A		Wavelength=0.71073
Cell:	a=36.535(7)	b=7.6974(15)	c=9.6111(18)
	alpha=90	beta=90	gamma=90
Temperature:	193 K		

	Calculated	Reported
Volume	2702.9(9)	2702.8(9)
Space group	P n a 21	P n a 21
Hall group	P 2c -2n	P 2c -2n
Moiety formula	C18 H12 O S	?
Sum formula	C18 H12 O S	C18 H12 O S
Mr	276.34	276.34
Dx,g cm-3	1.358	1.358
Z	8	8
Mu (mm-1)	0.231	0.231
F000	1152.0	1152.0
F000'	1153.43	
h,k,lmax	49,10,12	48,10,12
Nref	6856[3628]	6793
Tmin,Tmax	0.986,0.991	0.678,0.746
Tmin'	0.966	

Correction method= # Reported T Limits: Tmin=0.678 Tmax=0.746
AbsCorr = MULTI-SCAN

Data completeness= 1.87/0.99 Theta(max)= 28.489

R(reflections)= 0.0542(4614) wR2(reflections)= 0.1122(6793)

S = 1.032 Npar= 361

The following ALERTS were generated. Each ALERT has the format
test-name_ALERT_alert-type_alert-level.
Click on the hyperlinks for more details of the test.

Alert level C

RINTA01_ALERT_3_C The value of Rint is greater than 0.12
Rint given 0.138
PLAT340_ALERT_3_C Low Bond Precision on C-C Bonds .... 0.00677 Ang.
PLAT906_ALERT_3_C Large K Value in the Analysis of Variance ...... 2.091 Check

---

Alert level G

PLAT020_ALERT_3_G The Value of Rint is Greater Than 0.12 .... 0.138 Report
PLAT910_ALERT_3_G Missing # of FCF Reflection(s) Below Theta(Min). 2 Note
2 0 0, 4 0 0,
PLAT912_ALERT_4_G Missing # of FCF Reflections Above STh/L= 0.600 9 Note
PLAT933_ALERT_2_G Number of HKL-OMIT Records in Embedded .res File 1 Note
2 0 0,
PLAT969_ALERT_5_G The 'Henn et al.' R-Factor-gap value .... 2.957 Note
Predicted wR2: Based on SigI**2 3.79 or SHELX Weight 10.87
PLAT978_ALERT_2_G Number C-C Bonds with Positive Residual Density. 3 Info

```
   0 ALERT level A = Most likely a serious problem - resolve or explain
   0 ALERT level B = A potentially serious problem, consider carefully
   3 ALERT level C = Check. Ensure it is not caused by an omission or oversight
   6 ALERT level G = General information/check it is not something unexpected

   0 ALERT type 1 CIF construction/syntax error, inconsistent or missing data
   2 ALERT type 2 Indicator that the structure model may be wrong or deficient
   5 ALERT type 3 Indicator that the structure quality may be low
   1 ALERT type 4 Improvement, methodology, query or suggestion
   1 ALERT type 5 Informative message, check
```

Datablock: 5

Bond precision: C-C = 0.0019 A Wavelength=0.71073

Cell:	a=14.7655(6)	b=6.7441(3)	c=14.7616(6)
	alpha=90	beta=114.8414(12)	gamma=90
Temperature:	193 K		

	Calculated	Reported
Volume	1333.95(10)	1333.95(10)
Space group	P 21/c	P 21/c
Hall group	-P 2ybc	-P 2ybc
Moiety formula	C18 H12 F2	?
Sum formula	C18 H12 F2	C18 H12 F2
Mr	266.28	266.28
Dx,g cm-3	1.326	1.326
Z	4	4
Mu (mm-1)	0.095	0.095
F000	552.0	552.0
F000'	552.31	
h,k,lmax	19,8,19	19,8,19
Nref	3171	3145
Tmin,Tmax	0.972,0.981	0.713,0.746
Tmin'	0.963	

Correction method= # Reported T Limits: Tmin=0.713 Tmax=0.746
AbsCorr = MULTI-SCAN

Data completeness= 0.992 Theta(max)= 27.851

R(reflections)= 0.0357(2540) wR2(reflections)= 0.0928(3145)

S = 1.031 Npar= 202

The following ALERTS were generated. Each ALERT has the format
test-name_ALERT_alert-type_alert-level.
Click on the hyperlinks for more details of the test.

Alert level C

```
PLAT911_ALERT_3_C Missing FCF Refl Between Thmin & STh/L=    0.600          4 Report
               -1  0  2,   5  0  2,   0  3  3,   0  1  6,
```

Alert level G

```
PLAT002_ALERT_2_G Number of Distance or Angle Restraints on AtSite          8 Note
PLAT168_ALERT_4_G The CIF-Embedded .res File Contains EXYZ Records          4 Report
PLAT171_ALERT_4_G The CIF-Embedded .res File Contains EADP Records          4 Report
PLAT176_ALERT_4_G The CIF-Embedded .res File Contains SADI Records          2 Report
PLAT177_ALERT_4_G The CIF-Embedded .res File Contains DELU Records          2 Report
PLAT178_ALERT_4_G The CIF-Embedded .res File Contains SIMU Records          2 Report
PLAT301_ALERT_3_G Main Residue  Disorder .............(Resd    1)         30% Note
PLAT432_ALERT_2_G Short Inter X...Y Contact  F1'       ..C10      .       2.93 Ang.
                                                   x,-1+y,z  =      1_545 Check
PLAT811_ALERT_5_G No ADDSYM Analysis: Too Many Excluded Atoms ....          ! Info
PLAT860_ALERT_3_G Number of Least-Squares Restraints ............           2 Note
PLAT912_ALERT_4_G Missing # of FCF Reflections Above STh/L=  0.600         21 Note
PLAT933_ALERT_2_G Number of HKL-OMIT Records in Embedded .res File          3 Note
                0  3  3,   5  0  2,   0  1  6,
PLAT969_ALERT_5_G The 'Henn et al.' R-Factor-gap value ..........       5.214 Note
              Predicted wR2: Based on SigI**2  1.78 or SHELX Weight  9.00
PLAT978_ALERT_2_G Number C-C Bonds with Positive Residual Density.         22 Info
```

0 **ALERT level A** = Most likely a serious problem - resolve or explain
0 **ALERT level B** = A potentially serious problem, consider carefully
1 **ALERT level C** = Check. Ensure it is not caused by an omission or oversight
14 **ALERT level G** = General information/check it is not something unexpected

0 ALERT type 1 CIF construction/syntax error, inconsistent or missing data
4 ALERT type 2 Indicator that the structure model may be wrong or deficient
3 ALERT type 3 Indicator that the structure quality may be low
6 ALERT type 4 Improvement, methodology, query or suggestion
2 ALERT type 5 Informative message, check

Datablock: 9

Bond precision:	C-C = 0.0043 A		Wavelength=0.71073
Cell:	a=7.4380(6)	b=21.9521(18)	c=16.1226(15)
	alpha=90	beta=92.945(3)	gamma=90
Temperature:	193 K		

	Calculated	Reported
Volume	2629.0(4)	2629.0(4)
Space group	P 21/n	P 21/n
Hall group	-P 2yn	-P 2yn
Moiety formula	C18 H12 S	?
Sum formula	C18 H12 S	C18 H12 S
Mr	260.34	260.34
Dx,g cm-3	1.316	1.315
Z	8	8
Mu (mm-1)	0.227	0.227
F000	1088.0	1088.0
F000'	1089.35	
h,k,lmax	9,27,20	9,27,20
Nref	5511	5475
Tmin,Tmax	0.986,0.993	0.660,0.742
Tmin'	0.964	

Correction method= # Reported T Limits: Tmin=0.660 Tmax=0.742
AbsCorr = MULTI-SCAN

Data completeness= 0.993 Theta(max)= 26.597

R(reflections)= 0.0599(3192) wR2(reflections)= 0.1074(5475)

S = 1.031 Npar= 343

The following ALERTS were generated. Each ALERT has the format
test-name_ALERT_alert-type_alert-level.
Click on the hyperlinks for more details of the test.

Alert level C

```
RINTA01_ALERT_3_C  The value of Rint is greater than 0.12
           Rint given   0.164
PLAT340_ALERT_3_C Low Bond Precision on  C-C Bonds ..............    0.00427 Ang.
PLAT906_ALERT_3_C Large K Value in the Analysis of Variance ......      5.487 Check
```

Alert level G

```
PLAT020_ALERT_3_G The Value of Rint is Greater Than 0.12 .........      0.164 Report
PLAT910_ALERT_3_G Missing # of FCF Reflection(s) Below Theta(Min).          1 Note
                0  1  1,
PLAT912_ALERT_4_G Missing # of FCF Reflections Above STh/L=  0.600         35 Note
PLAT933_ALERT_2_G Number of HKL-OMIT Records in Embedded .res File          1 Note
                0  1  1,
PLAT969_ALERT_5_G The 'Henn et al.' R-Factor-gap value ..........      2.721 Note
              Predicted wR2: Based on SigI**2  3.95 or SHELX Weight 10.42
PLAT978_ALERT_2_G Number C-C Bonds with Positive Residual Density.          4 Info
```

```
0 ALERT level A = Most likely a serious problem - resolve or explain
0 ALERT level B = A potentially serious problem, consider carefully
3 ALERT level C = Check. Ensure it is not caused by an omission or oversight
6 ALERT level G = General information/check it is not something unexpected

0 ALERT type 1 CIF construction/syntax error, inconsistent or missing data
2 ALERT type 2 Indicator that the structure model may be wrong or deficient
5 ALERT type 3 Indicator that the structure quality may be low
1 ALERT type 4 Improvement, methodology, query or suggestion
1 ALERT type 5 Informative message, check
```

Datablock: 10

Bond precision: C-C = 0.0024 A Wavelength=0.71073

Cell:	a=25.6615(17)	b=8.0439(5)	c=13.7786(8)
	alpha=90	beta=95.872(3)	gamma=90
Temperature:	193 K		

	Calculated	Reported
Volume	2829.2(3)	2829.2(3)
Space group	C 2/c	C 2/c
Hall group	-C 2yc	-C 2yc
Moiety formula	C18 H12 O2 S	?
Sum formula	C18 H12 O2 S	C18 H12 O2 S
Mr	292.34	292.34
Dx,g cm-3	1.373	1.373
Z	8	8
Mu (mm-1)	0.229	0.229
F000	1216.0	1216.0
F000’	1217.51	
h,k,lmax	34,10,18	34,10,18
Nref	3538	3521
Tmin,Tmax	0.947,0.973	0.680,0.746
Tmin’	0.912	

Correction method= # Reported T Limits: Tmin=0.680 Tmax=0.746
AbsCorr = MULTI-SCAN

Data completeness= 0.995 Theta(max)= 28.351

R(reflections)= 0.0432(3029) wR2(reflections)= 0.1197(3521)

S = 1.079 Npar= 190

The following ALERTS were generated. Each ALERT has the format
test-name_ALERT_alert-type_alert-level.
Click on the hyperlinks for more details of the test.

Alert level C

```
PLAT230_ALERT_2_C Hirshfeld Test Diff for    C15      --C16      .        6.6 s.u.
PLAT911_ALERT_3_C Missing FCF Refl Between Thmin & STh/L=    0.600         12 Report
                0  2  0,   2  2  0,   4  0  0,  -3  1  1,  -1  1  1,   5  1  1,
               -6  0  2,  -3  1  2,   0  0  2,   2  0  2,  -8  0  4,   0  0  4,
PLAT913_ALERT_3_C Missing # of Very Strong Reflections in FCF ....          7 Note
                0  2  0,   2  2  0,   4  0  0,  -6  0  2,   0  0  2,  -8  0  4,
                0  0  4,
```

Alert level G

```
PLAT910_ALERT_3_G Missing # of FCF Reflection(s) Below Theta(Min).          1 Note
                2  0  0,
PLAT912_ALERT_4_G Missing # of FCF Reflections Above STh/L=  0.600          4 Note
PLAT969_ALERT_5_G The 'Henn et al.' R-Factor-gap value ..........      6.214 Note
              Predicted wR2: Based on SigI**2  1.93 or SHELX Weight 11.09
PLAT978_ALERT_2_G Number C-C Bonds with Positive Residual Density.         18 Info
```

0 **ALERT level A** = Most likely a serious problem - resolve or explain
0 **ALERT level B** = A potentially serious problem, consider carefully
3 **ALERT level C** = Check. Ensure it is not caused by an omission or oversight
4 **ALERT level G** = General information/check it is not something unexpected

0 ALERT type 1 CIF construction/syntax error, inconsistent or missing data
2 ALERT type 2 Indicator that the structure model may be wrong or deficient
3 ALERT type 3 Indicator that the structure quality may be low
1 ALERT type 4 Improvement, methodology, query or suggestion
1 ALERT type 5 Informative message, check

It is advisable to attempt to resolve as many as possible of the alerts in all categories. Often the minor alerts point to easily fixed oversights, errors and omissions in your CIF or refinement strategy, so attention to these fine details can be worthwhile. In order to resolve some of the more serious problems it may be necessary to carry out additional measurements or structure refinements. However, the purpose of your study may justify the reported deviations and the more serious of these should normally be commented upon in the discussion or experimental section of a paper or in the "special_details" fields of the CIF. checkCIF was carefully designed to identify outliers and unusual parameters, but every test has its limitations and alerts that are not important in a particular case may appear. Conversely, the absence of alerts does not guarantee there are no aspects of the results needing attention. It is up to the individual to critically assess their own results and, if necessary, seek expert advice.

Publication of your CIF in IUCr journals

A basic structural check has been run on your CIF. These basic checks will be run on all CIFs submitted for publication in IUCr journals (*Acta Crystallographica*, *Journal of Applied Crystallography*, *Journal of Synchrotron Radiation*); however, if you intend to submit to *Acta Crystallographica Section C* or *E* or *IUCrData*, you should make sure that full publication checks are run on the final version of your CIF prior to submission.

Publication of your CIF in other journals

Please refer to the *Notes for Authors* of the relevant journal for any special instructions relating to CIF submission.

PLATON version of 02/02/2025; check.def file version of 02/02/2025

Datablock exo_1 - ellipsoid plot

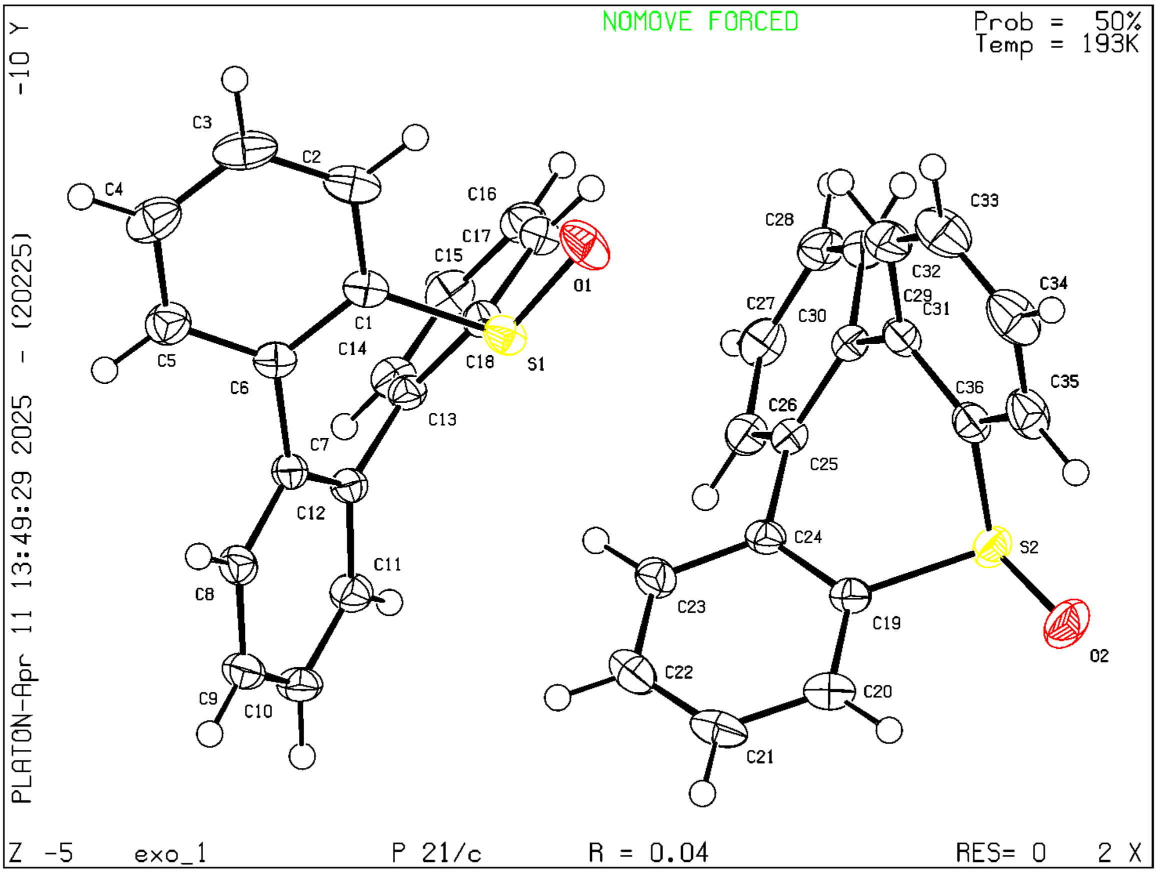

Datablock endo_1 - ellipsoid plot

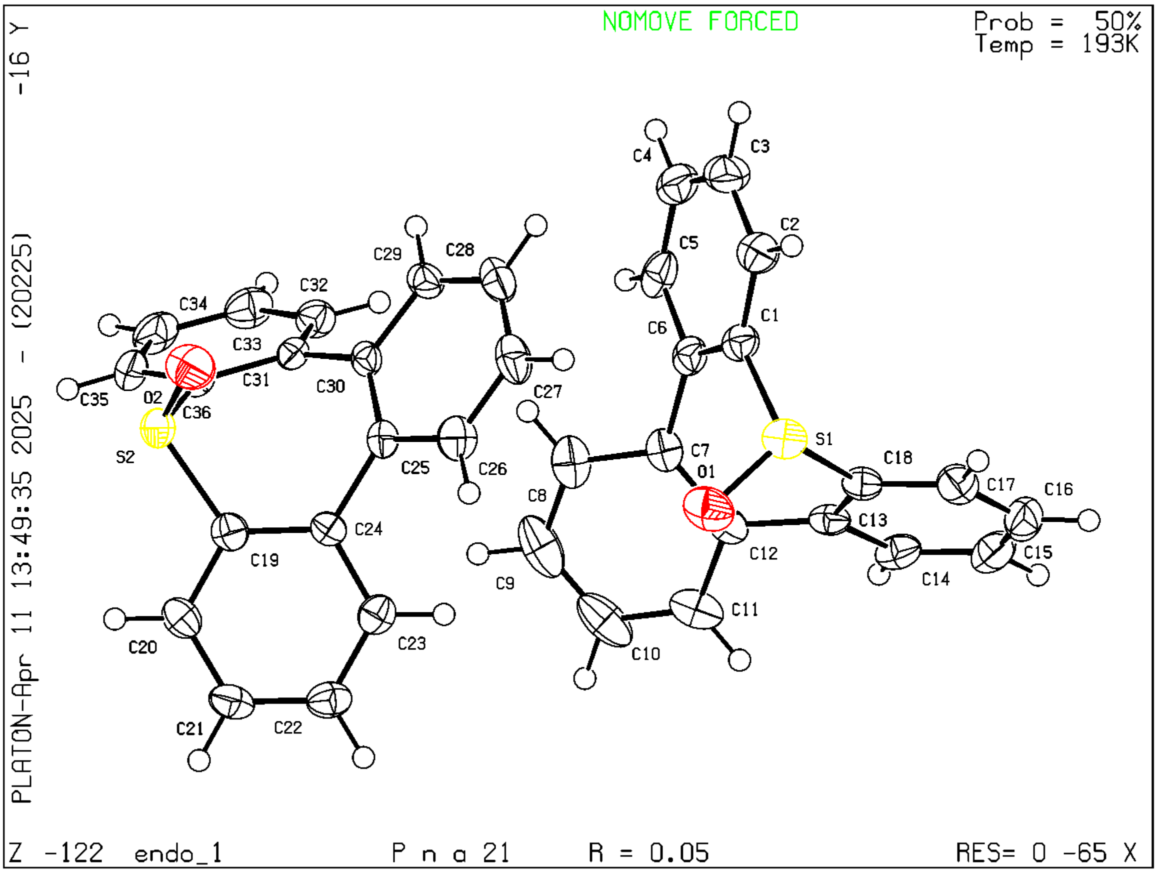

Datablock 5 - ellipsoid plot

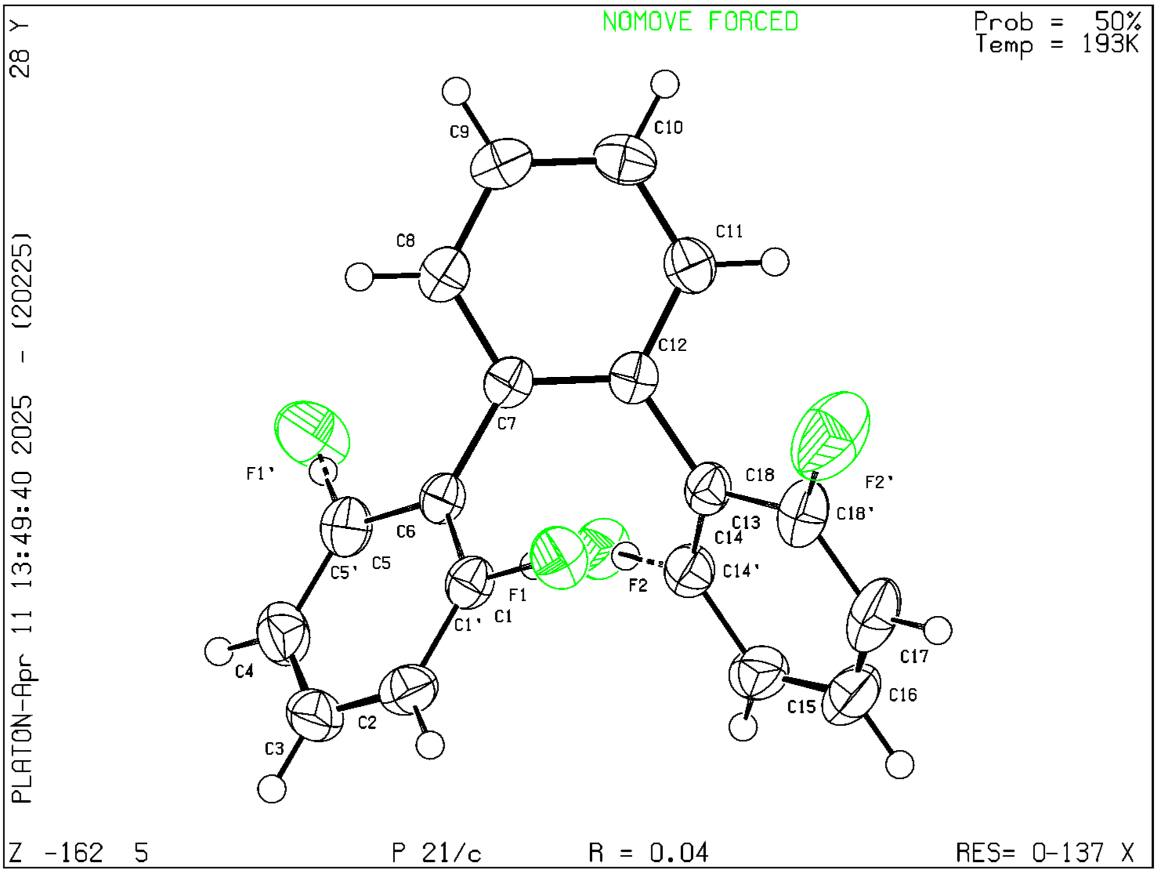

Datablock 9 - ellipsoid plot

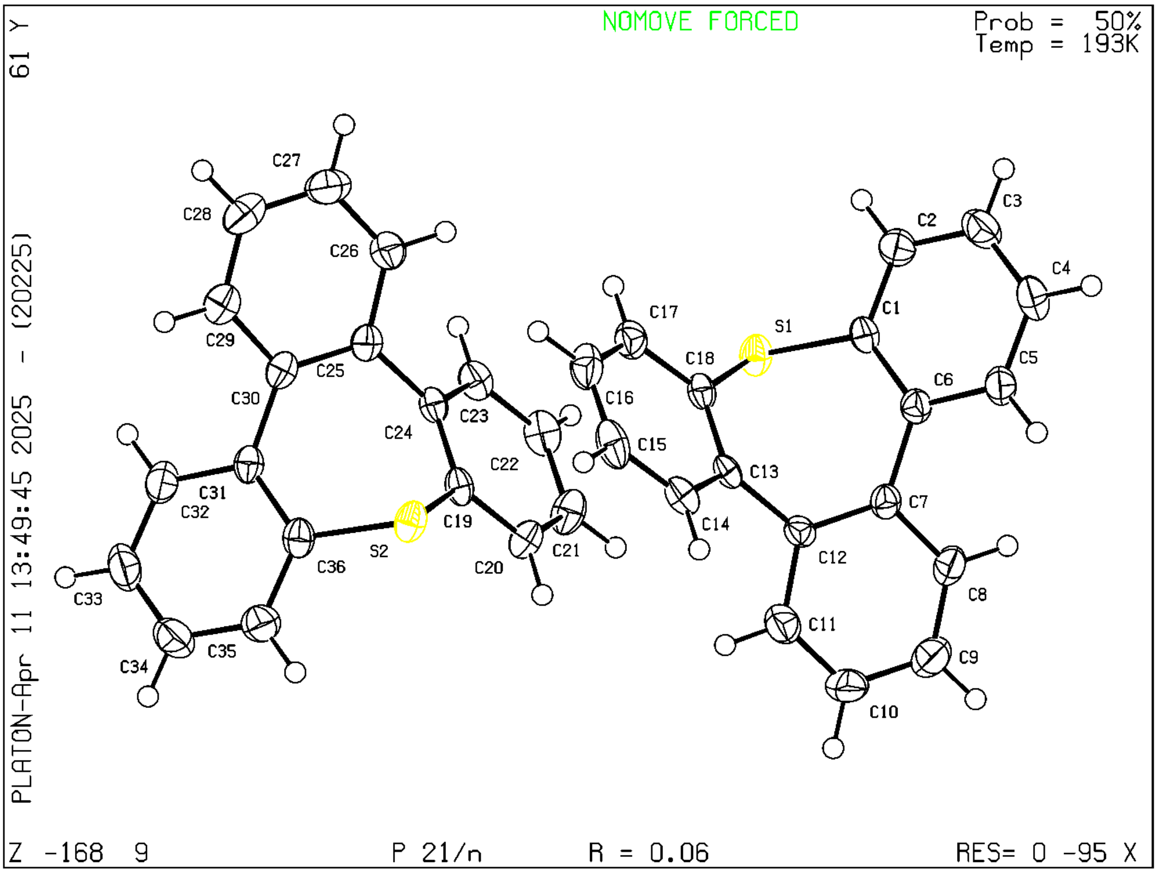

Datablock 10 - ellipsoid plot

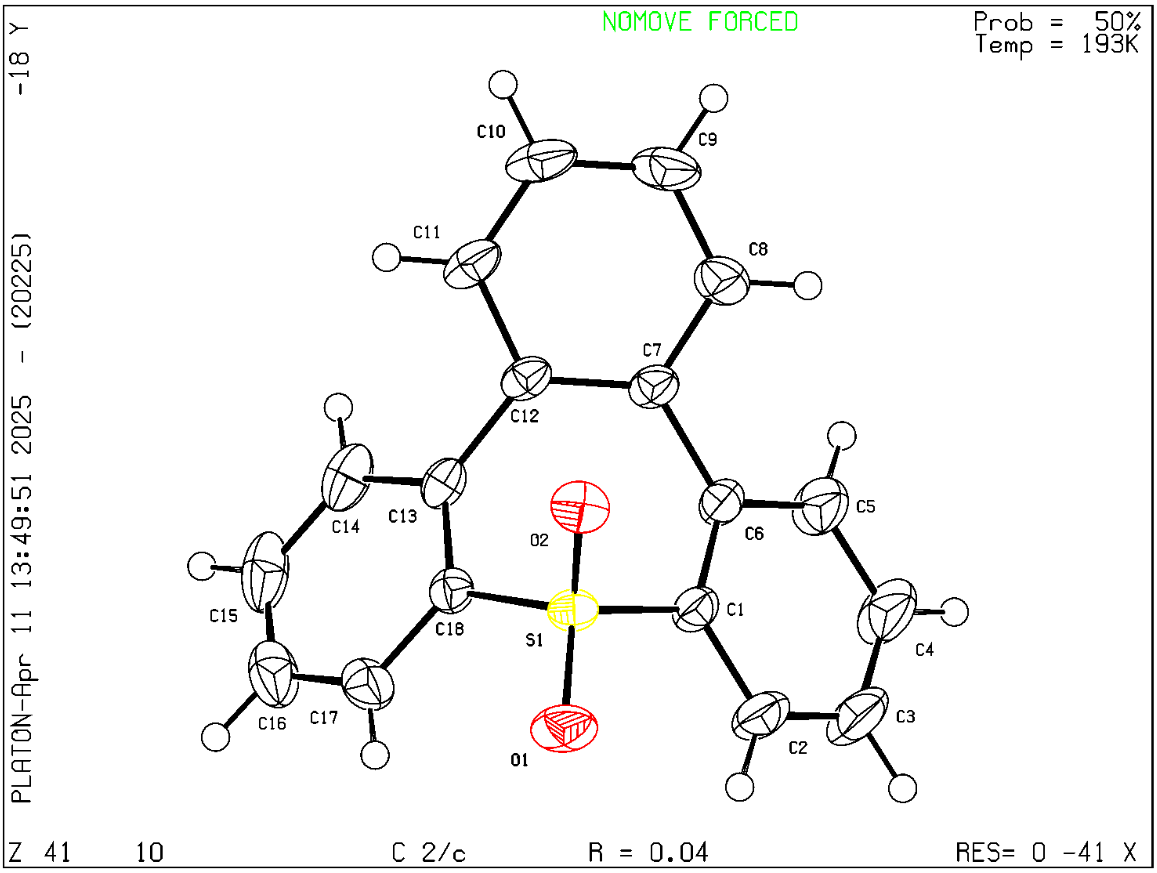

Supplement: Supplementary file 3 — Supporting Information [file CHEM-31-e02655-s001.pdf]
